# The mechanism of MinD stability modulation by MinE in Min protein dynamics

**DOI:** 10.1371/journal.pcbi.1011615

**Published:** 2023-11-17

**Authors:** William C. Carlquist, Eric N. Cytrynbaum

**Affiliations:** Department of Mathematics, University of British Columbia, Vancouver, BC, Canada; Clemson University, UNITED STATES

## Abstract

The patterns formed both *in vivo* and *in vitro* by the Min protein system have attracted much interest because of the complexity of their dynamic interactions given the apparent simplicity of the component parts. Despite both the experimental and theoretical attention paid to this system, the details of the biochemical interactions of MinD and MinE, the proteins responsible for the patterning, are still unclear. For example, no model consistent with the known biochemistry has yet accounted for the observed dual role of MinE in the membrane stability of MinD. Until now, a statistical comparison of models to the time course of Min protein concentrations on the membrane has not been carried out. Such an approach is a powerful way to test existing and novel models that are difficult to test using a purely experimental approach. Here, we extract time series from previously published fluorescence microscopy time lapse images of *in vitro* experiments and fit two previously described and one novel mathematical model to the data. We find that the novel model, which we call the Asymmetric Activation with Bridged Stability Model, fits the time-course data best. It is also consistent with known biochemistry and explains the dual MinE role via MinE-dependent membrane stability that transitions under the influence of rising MinE to membrane instability with positive feedback. Our results reveal a more complex network of interactions between MinD and MinE underlying Min-system dynamics than previously considered.

## Author summary

The Min protein system in *E. coli* is important both for its functional role in cell division and as a relatively simple biochemical network that demonstrates remarkable and diverse spatiotemporal patterning both *in vivo* and *in vitro*. There is a rich history of mathematical modeling in the field but the focus has been largely qualitative, and some experimental observations have remained beyond the explanatory scope of proposed mechanisms. Here we fit models to quantitative time-course data from *in vitro* experiments to test various hypothesis that would be difficult to test experimentally. We propose a new model that explains the data best, is consistent with experimental observations, and points to a two-phase stability-switching process underlying patterning.

## Introduction

The Min system of *Escherichia coli* is one of the simplest known biological systems that demonstrates diverse complex spatiotemporal behavior. It consists of three proteins, MinC, MinD, and MinE, which together dynamically regulate the position of the cell division site. Interactions of MinD and MinE on the cellular membrane drive coupled oscillations of the three Min proteins from cell pole to cell pole [1–6], while MinC locally inhibits the formation of the Z-ring, the contractile ring that divides the cell in two [4, 7–11]. The character of Min-protein oscillations differs with cell length and shape. In rod-shaped cells, coupled densities of MinD and MinE stochastically switch from cell pole to cell pole in short cells [12], oscillate regularly from cell pole to cell pole in mid-sized cells [1, 12, 13], and oscillate regularly from cell pole to midcell in long mutant cells [1, 13]. In round mutant cells, densities of MinD and MinE oscillate antipodally [14, 15], and in branched mutant cells, densities of MinD and MinE oscillate from branch to branch to branch [16]. MinD and MinE also form dynamic patterns *in vitro*, including some *in vivo* behaviors reconstituted in microdroplets [17] and larger membrane-clad compartments [18, 19]. Perhaps most strikingly, on supported lipid bilayers, densities of MinD and MinE undergo spatially uniform oscillations in time [20] and form dynamic patterns such as traveling waves [20–24], spiral waves [20–24], amoeba-like shapes [20, 24], snake-like projections [20], mushroom-like shapes [24], and bursts [24] as well as an array of static patterns including spots, labyrinths, and meshes [25].

**Fig 1:**
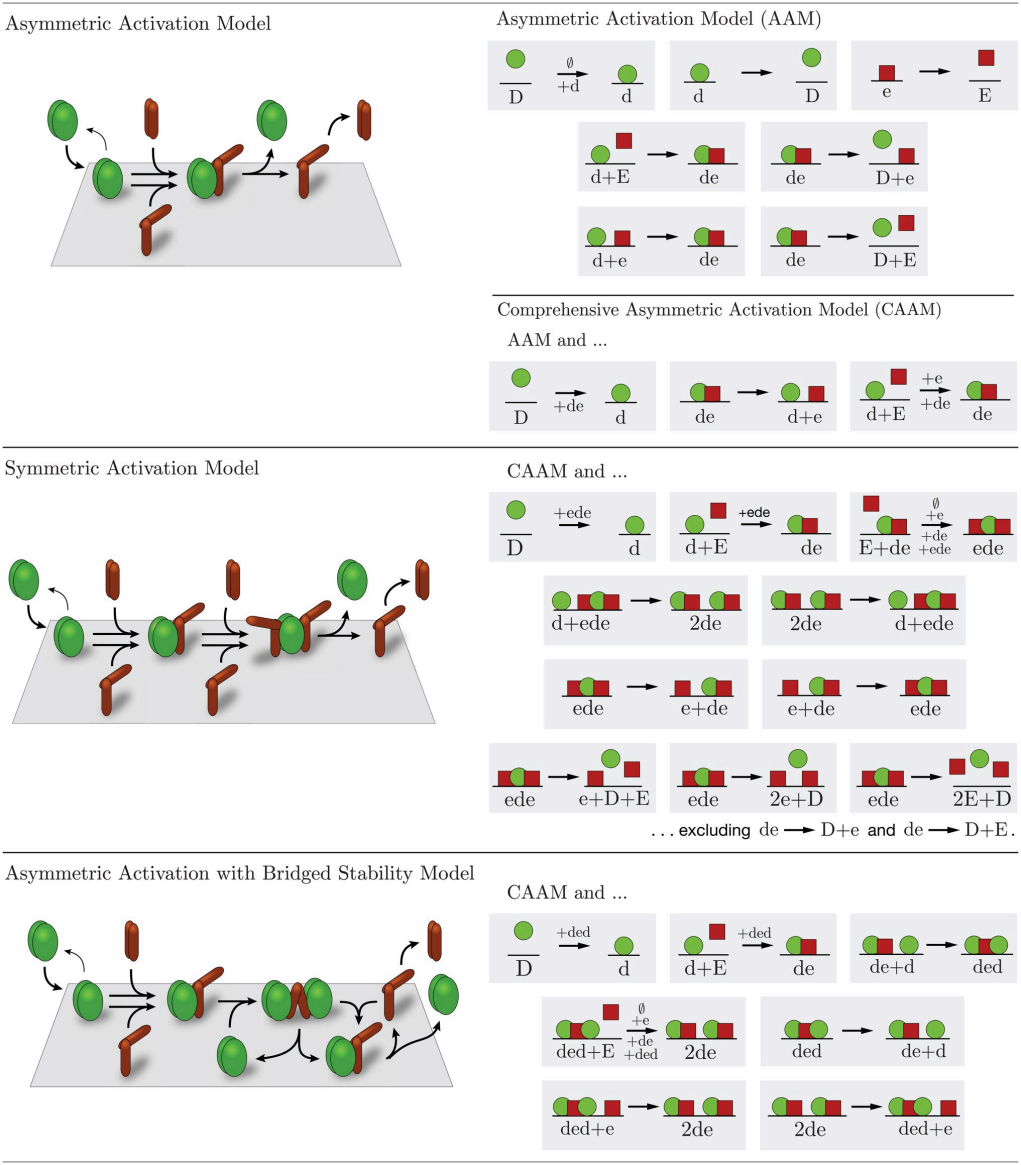
Illustration of the Min-system models considered. Conceptual representations of the models are on the left with several reactions omitted for clarity. A full accounting of reactions included in each model is on the right. A reaction arrow with a +x indicates that the reaction is facilitated by the density of species x. An unfacilitated basal rate is annotated with a ∅ when shown with a facilitated reaction. Membrane is denoted by a grey surface (left) and black line (right). Top panel: The AAM and CAAM—MinD dimers (green) bind to the membrane and recruit MinE dimers (red) which then induce ATP-hydrolysis in MinD dimers, causing MinD dimers, and subsequently MinE dimers, to dissociate from the membrane. Middle panel: The SAM—With a single MinE attached, ATP-hydrolysis in MinD dimers is not induced and MinD dimers are stabilized on the membrane. With two MinEs attached, hydrolysis is induced. Bottom panel: The AABSM—This model includes the CAAM at its core but adds the possibility of a second MinD dimer binding to MinE, stabilizing both MinD dimers on the membrane. We hypothesize that the strain imposed on a MinE dimer from bridging two MinD dimers alters MinE’s interaction with MinD, disrupting induction of ATP hydrolysis in either MinD dimer.

Biochemistry, crystallography, and mutant studies have together elucidated many details of the protein structures and reactions that underly the emergent macroscopic behavior of the MinDE system. They have found that cytosolic MinD monomers bind to ATP and form dimers [5, 26, 27], which bind to a phospholipid membrane [5, 6, 21, 26, 28, 29] and cooperatively recruit other cytosolic MinD dimers to bind to the membrane [6]. Additionally, cytosolic MinE dimers bind to MinD dimers on the membrane [22, 28], bind to the membrane [30], and stimulate ATPase activity in the bound MinD dimers, causing the MinD dimers to separate and dissociate from the membrane [5, 6, 28, 31] while the MinE dimers transiently remain bound to the membrane [24, 32, 33] before returning to the cytosol. This characterization of MinDE kinetics is depicted in the top panel of Fig 1 and referred to here as the Asymmetric Activation Model (AAM).

In bridging the gap between protein interactions and emergent patterning, mathematical modeling of the Min system has a long and rich history. Most mathematical models of MinDE dynamics are based on a subset of the reactions described above, and generally their aim has been to recapitulate behaviors of the Min system *in vivo*. They have successfully demonstrated oscillatory behaviors particular to short cells [12, 34], mid-sized cells [35–40], long cells [34, 36, 38, 40–42], dividing cells [40, 42–44], aberrant cellular geometries [16, 34, 45–48], and MinE mutants [49, 50]. Several mathematical models have recapitulated behaviors of the Min system *in vitro*, including traveling waves [21, 51] and spiral waves [21, 34] on supported lipid bilayers and patterning on geometrically confined membranes [52] and micropatterned substrates [53].

Various quantitative experimental measurements have been used to validate different mathematical models of the Min system: pole-to-pole oscillation period *in vivo* [34, 40–42, 47, 49, 50, 54], distributions of residence times during stochastic pole-to-pole switching [12, 34] and regular pole-to-pole oscillations [12] *in vivo*, and traveling wave velocity and wavelength *in vitro* [21]. However, no model has been quantitatively compared to time-course data. A quantitative comparison of how well models fit time-course data is the next logical step in model selection and validation.

**Fig 2:**
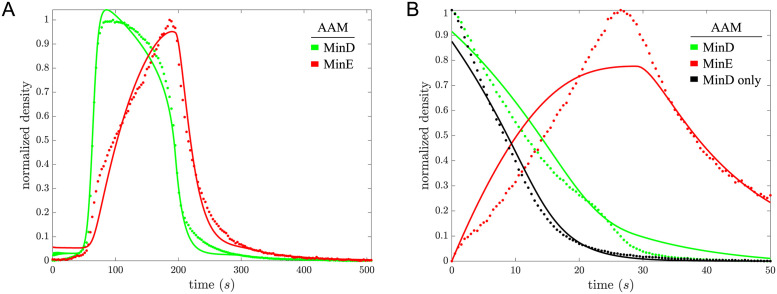
For the Asymmetric Activation Model (AAM), the fit to the oscillation data is shown in Panel A, and the fit to MinD dissociation data is shown in Panel B. Fits are solid, data are dotted. We omit SEMs in the data from the plot because they would be indistinguishable from the means shown by eye. The data has been scaled and shifted so as to span 0 to 1, and the model has been modified with the same transformation in this and the next figure to ensure fits to both MinD and MinE time courses are clearly visible. See S2 Fig for the data plotted in units of monomers per *μ*m^2^.

In Ivanov and Mizuuchi’s *in vitro* experiments [20], buffer was flowed atop a supported lipid bilayer to ensure spatially uniform concentrations of reaction components in the buffer. On the supported lipid bilayer, densities of MinD and MinE oscillated nearly homogeneously in space before forming into traveling waves. Because of constant bulk conditions from buffer flow in Ivanov and Mizuuchi’s experiments, the dynamics of the Min system they observed do not depend on bulk reactions, such as ATP binding and dimerization of MinD, and are not subject to effects from gradients of reaction components in the bulk, the focus of some recent studies [55–57]. This set-up greatly simplifies the modelling. More details about the experimental set-up and our model of it can be found in the first section of the SI Text. To further limit the dependence on space, we find regions on the membrane in which the oscillation data from Ivanov and Mizuuchi’s experiments have the least amount of spatial variation and extract one period of the nearly spatially-homogeneous oscillation time-course data from it, hereafter referred to as *oscillation data*. Under spatially uniform conditions, systems of partial differential equations (PDEs) that model how membrane-bound Min protein concentrations evolve in space and time reduce to systems of ordinary differential equations (ODEs) that describe just the local reactions. Fitting ODE models to the oscillation data allows us to compare different reaction-mechanism models of MinD and MinE on the supported lipid bilayer without complications from spatial effects but still giving insight into patterning. The oscillation data are shown as dots in Panel A of Fig 2, and details of data extraction are discussed in the Materials and Methods.

Vecchiarelli *et al*. showed that, in addition to acting as an inhibitor of MinD membrane binding, at times MinE can stabilize MinD on the membrane [24]. In *in vitro* experiments similar to those of Ivanov and Mizuuchi, they flowed buffer with and without MinE atop a layer of MinD bound to a supported lipid bilayer. Initially, MinD dissociated more slowly from the membrane with MinE in the flowed buffer than it did without MinE in the flowed buffer. Later, the concentration of membrane-bound MinE increased and MinD dissociated more rapidly from the membrane than it did without MinE in the flowed buffer. Fitting ODE models to Vecchiarelli *et al*.’s MinD dissociation data with and without MinE in the flowed buffer allows us to compare how well different reaction-mechanism models recapitulate MinE’s dual role in MinD membrane binding. The MinD dissociation data with and without MinE in the flowed buffer, hereafter referred to as *MinD dissociation data*, are shown as dots in Panel B of Fig 2. We refer to the oscillation data and the MinD dissociation data together as the *time-course data*.

The mechanism underlying MinE’s dual role in MinD membrane binding is unclear. No mathematical model has accounted for it and its biological implications remain unknown. The only current hypothesis proposes that one bound MinE dimer anchors a MinD dimer to the membrane, whereas two bound MinE dimers stimulate ATPase activity in a MinD dimer, causing it to separate and dissociate from the membrane [24]. We formulate this mechanism into a mathematical model that we call the Symmetric Activation Model (SAM). Mutational studies have shown, however, that a single MinE dimer bound to a MinD dimer is sufficient to stimulate ATPase activity in the MinD dimer [58]. Based on this finding, we propose a new model, the Asymmetric Activation with Bridged Stability Model (AABSM), that accounts for MinE’s dual role in MinD membrane binding and requires only one bound MinE dimer to stimulate ATPase activity in a MinD dimer. Both these models are illustrated in Fig 1 with details given in the Materials and Methods and in the Supporting Information text (S1 Text).

We fit the SAM and the AABSM to the oscillation data and the MinD dissociation data using the Homotopy-Minimization Method for parameter estimation in differential equations, a method with accuracy gauges shown to find optimal-data-fitting numerical solutions more robustly and more efficiently than a direct numerical-integration approach [59]. For comparison, we also fit a mathematical version of the AAM to the time-course data as well as a slightly modified version of the AAM referred to as the Comprehensive Asymmetric Activation Model (CAAM) and a generic excitability model (the FitzHugh-Nagumo model). Examining how models fit the time-course data allows us to distinguish between biochemical assumptions in the various models. Our model fitting also provides us with time courses for hidden protein states that would be difficult or impossible to measure experimentally.

We also use the optimal parameters for each of the models to test out the models’ behavior in a spatial context, to verify that the models support the *in vitro* spatiotemporal patterns observed under the same experimental conditions as the fitted data (travelling and spiral waves).

Ultimately, our analysis supports the AABSM and suggests that, through a more complex web of interactions, activations, and inhibitions between MinD and MinE than previously considered, Min-system pattern formation is driven by a MinE-dependent reinforced stability of MinD on the membrane followed by a MinE-dictated switch to membrane instability with positive feedback.

## Results

### Fitting the Asymmetric Activation Model

Amongst previously published mathematical models, the model of Bonny *et al*. [34] (henceforth the Bonny Model) demonstrates the most diverse array of behaviors that are qualitatively similar to experimental observations of the Min system *in vivo* and *in vitro*, including stochastic pole-to-pole switching in short cells, regular pole-to-pole oscillations in mid-sized cells, oscillation splitting in growing cells, regular pole-to-midcell oscillations in long cells, end-to-end oscillations in thick cells, and spiral waves on a supported lipid bilayer. In the Bonny Model, bulk MinD dimers bind to the membrane (**D** → **d**), and membrane-bound MinD dimers cooperatively recruit more bulk MinD dimers to bind to the membrane ($D\overset{+d}{\to }d$) or bind to bulk MinE dimers forming membrane-bound MinD-MinE complexes (**d,E** → **de**). Then, MinE dimers in the **de** complex stimulate ATPase activity in bound MinD dimers, causing the MinD dimers to dissociate from the membrane while the MinE dimers either return to the bulk (**de** → **D,E**) or temporarily remain on the membrane (**de** → **D,e**). Finally, membrane-bound MinE dimers either dissociate from the membrane (**e** → **E**) or bind to membrane-bound MinD dimers, reforming membrane-bound MinD-MinE complexes (**d,e** → **de**). Beyond the aforementioned reactions, bulk species diffuse in the bulk and membrane-bound species diffuse on the membrane in the Bonny Model. Given the Bonny Model’s ability to qualitatively recapitulate Min-system patterning, we wanted to see how well it could describe the time-course data.

We modified the Bonny Model to be consistent with experimental conditions and outcomes underlying the time-course data to get the AAM, so named to emphasize that a single MinE dimer is sufficient to activate hydrolysis in the MinD dimer. Details of the modifications are discussed in the Materials and Methods. All reactions included in the AAM are shown in Fig 1 and the corresponding system of ODEs is given by Eq. S3.

Fitting the AAM to the time-course data, as described in the Material and Methods, we found reasonably good qualitative agreement, but certain details differed as can be seen in Fig 2. For the oscillation data, after peaking too high, the fit to the MinD density drops too quickly at first, has a similar maximal rate of decrease compared to the time-course data, and then drops too quickly again in the tail of the pulse. Additionally, the AAM also fails to recapitulate the near linear rise in MinE density from roughly 70 s until 190 s. As with MinD, the final decay of MinE back to steady state is also too rapid. For the MinD dissociation data with MinE in the flowed buffer, as was expected given that the AAM treats MinE solely as an inhibitor of MinD membrane binding, the AAM fails to recapitulate the knee in MinD density around 25 s, when MinE shifts from stabilizing to destabilizing MinD on the membrane. Additionally, the AAM fails to recapitulate the increasing rate of net MinE attachment from roughly 5 s until 20 s. Instead, the MinE density rises quickly at first and then tapers off, seemingly, as the amount of MinD available for it to bind to on the membrane drops off. Furthermore, we note that although the AAM appears to qualitatively describe the time-course data fairly well, a variant of the FitzHugh-Nagumo Model (FHNM), a simplified model of neuron firing, qualitatively describes the time-course data almost as well as the AAM, as can be seen in Table 1 and S5 Fig. As such, we caution that what appears to be a reasonably good qualitative agreement with data does not necessarily qualify as a good biochemical model. In Table 1, we show the relative *χ*^2^ statistic and Akaike Information Criterion score (AIC) for all models considered. These quantities clarify that neither the FHNM nor the AAM do particularly well in explaining the time-course data, but that the AAM does better than the FHNM.

**Table 1:** Model Comparison. *χ*^2^ is the the weighted sum of squared residuals, AIC is the Akaike Information Criterion score, $\chi_{\text{min}}^{2}$ is the minimum *χ*^2^ value amongst the models, AIC_min_ is the minimum AIC value amongst the models, and *n* is the number of fitted model parameters. The AIC is an estimate of the information lost by using the model to represent the data and accounts for the fitting benefit of having more parameters; it decreases as the fit to data improves and increases with the number of parameters in the model. Here, effectively, AIC = *N* ln(*χ*^2^) + 2*m*, where *N* is the number of data values and *m* is the number of non-zero model parameters plus the number of fitted initial conditions [60]. As a rule of thumb, a difference in AIC of more than 10 between two models is strong evidence in favor of the model with the lower AIC score [61]. By both *χ*^2^ and AIC measures, the AABSM clearly describes both sets of time-course data best.

	Oscillation Data			MinD Dissociation Data		
	$\chi^{2}/\chi_{\text{min}}^{2}$	AIC − AIC_min_	*n*	$\chi^{2}/\chi_{\text{min}}^{2}$	AIC − AIC_min_	*n*
FHNM	90	1500	9	260	1600	10
AAM	52	1300	14	150	1500	13
CAAM	14	880	18	15	800	16
SAM	1.8	200	29	3.2	360	26
AABSM	1	0	28	1	0	25

### Fitting the Comprehensive Asymmetric Activation Model

Based on experimental observations since the publication of the Bonny Model, we extended the AAM to include additional reactions (see the S1 Text for details), then fit the resulting Comprehensive Asymmetric Activation Model (CAAM) to the time-course data to see if the inclusion of the new reactions markedly improves data fitting. The reactions included in the CAAM are shown in the top panel of Fig 1, and the CAAM is written as a system of ODEs in Eq. S4.

We found that the CAAM fits the time-course data substantially better than the AAM, as can qualitatively be seen in S3 Fig and is quantitatively shown by *χ*^2^ statistics and AIC scores in Table 1.

The reaction $D\overset{+de}{\to }d$ allows recruitment of MinD to the membrane throughout the pulse, and its inclusion in the CAAM appears to have alleviated a peak overshoot followed by an accelerated drop-off of MinD in the fit to the oscillation data. The inclusion of facilitated MinE recruitment (reactions $E+d\overset{+e,de}{\to }de$) in the CAAM appears to have allowed for a better match to the observed constant rate of net MinE-membrane attachment between 70 s and 190 s in the oscillation data and the increasing rate of net MinE-membrane attachment from roughly 5 s until 20 s in the MinD dissociation data with MinE in the flowed buffer, presumably by increasing facilitation even as the bindable amount of MinD on the membrane decreases. Ultimately, the CAAM appears to fit the oscillation data fairly well. However, like the AAM, the CAAM is unable to demonstrate MinE’s dual role as both a stabilizer and a destabilizer of MinD membrane binding.

### Fitting the Symmetric Activation Model

To account for MinE’s dual role as both a stabilizer and a destabilizer of MinD membrane binding, Vecchiarelli *et al*. hypothesized that the binding of a single MinE dimer to a membrane-bound MinD dimer is not sufficient to stimulate ATPase activity in the MinD dimer [24]. Rather, they proposed that the binding of a single MinE dimer to a membrane-bound MinD dimer stabilizes the MinD dimer on the membrane through MinE’s interaction with the membrane, and the subsequent binding of a second MinE dimer to the membrane-bound MinD dimer stimulates ATPase activity in the MinD dimer, causing it to dissociate from the membrane. A simplified version of this biochemical arrangement was also proposed previously in a modeling study with different motivation before it was known that MinE played multiple roles in MinD’s interaction with the membrane [51]. No other hypothesis has been proposed to explain MinE’s dual role in MinD-membrane binding.

We formulated Vecchiarelli *et al*.’s hypothesis in a mathematical model that we call the Symmetric Activation Model (SAM) because it requires two MinE dimers, one attached to each MinD-dimer subunit, to induce removal of a MinD dimer from the membrane. In doing so, we included a wide range of possible reactions in the SAM because we had no experimental basis for the full set of reactions that should be included in it. Details are given in the Materials and Methods. The SAM reactions are shown in the middle panel of Fig 1, and the model is written as a system of ODEs in Eq. S5.

We found that the optimal data-fitting parameter choice for the SAM provides a better fit to both sets of time course data than the CAAM (see S4 Fig and Table 1). Most notably, for the MinD dissociation data with MinE in the flowed buffer, the SAM partially recapitulates the knee in MinD density around 25 s, when MinE shifts from stabilizing to destabilizing MinD-membrane binding, whereas the CAAM does not. However, the SAM demonstrates a less dynamic shift than is visible in the data.

### Fitting the Asymmetric Activation with Bridged Stability Model

Contradicting the underlying assumption of the SAM, experiments have shown that a single MinE dimer bound to one subunit of a MinD dimer stimulates ATPase activity in both subunits of the MinD dimer [58], supporting the theory that MinE dimers stimulate ATPase activity in MinD dimers asymmetrically rather than symmetrically. Recent mass-sensitive particle tracking analysis has shown that MinD and MinE form very large heteromeric complexes and that larger MinD-MinE heteromers have longer membrane residence times than smaller MinD-MinE heteromers and MinD homomers with the same number of MinD subunits [62]. Apart from this stabilization-by-aggregation characterization, in which MinE seemingly acts only as a stabilizer and not a destabilizer of MinD membrane binding, there is no direct evidence for the structure of a membrane-stable MinD-MinE complex. One crystal structure shows a MinE dimer bridging two MinD dimers, but in it one MinD dimer is rotated 90° with respect to the other MinD dimer [30], which has led to the structure being considered an experimental artifact rather than a biologically relevant state. Despite this, we propose that a MinE dimer bridging two MinD dimers (**ded**) may be the membrane-stable MinD-MinE complex. We hypothesize that the strain associated with the 90° rotation required to ensure both MinD dimers can bind to the membrane inhibits ATPase activity in both MinD dimers to a level below the activity in a **de** complex, by perturbing both MinD-MinE interfaces from that in the **de** complex. Furthermore, we assume that the additional membrane anchors in the **ded** complex stabilize the complex as compared to the **d**-dimer. This latter assumption provides an explanation for the slower observed detachment of MinD in the presence of MinE as compared to in its absence. These assumptions guided our formulation of the AABSM.

Like in the SAM, we included a wide range of possible reactions in the AABSM, shown in the bottom panel of Fig 1. The model is written as a system of ODEs in Eq. S6.

**Fig 3:**
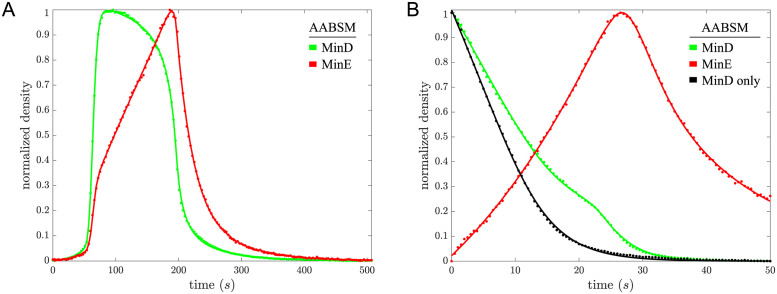
For the Asymmetric Activation with Bridged Stability Model (AABSM), the fit to the oscillation data is shown in Panel A, and the fit to MinD dissociation data is shown in Panel B. Data is shown as dots with colors for MinD and MinE matching the model colors. As in Fig 2, we omit SEMs in the data because they would be indistinguishable from the means shown by eye.

We found that the AABSM fits both time-course data sets better than the SAM (see Fig 3 and Table 1) and could describe all features of the time-course data, including the knee in MinD density at 25 s in the MinD dissociation data with MinE in the flowed buffer when MinE shifts from MinD-stabilizing to MinD-destabilizing, a feature that was not well captured by any of the other three models.

**Fig 4:**
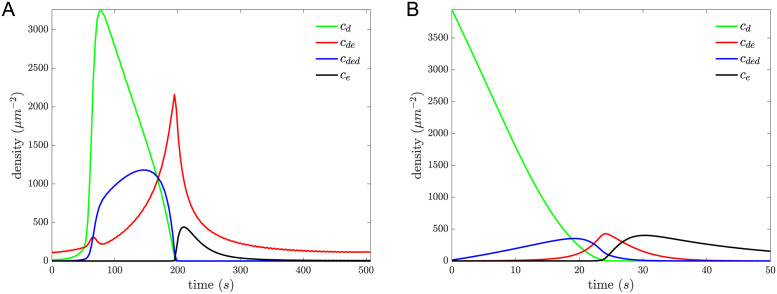
For the AABSM, state values from the fit to the oscillation data are shown in Panel A, and state values from the fit to MinD dissociation data with MinE in the flowed buffer are shown in Panel B. *c*_*d*_ (green) is the concentration of membrane-bound MinD dimers (**d**), the membrane-semistable MinD state; *c*_*de*_ (red) is the concentration of MinE dimers bound to one MinD dimer (**de**), the membrane-unstable MinD state; *c*_*ded*_ (blue) is the concentration of MinE dimers bridging two MinD dimers (**ded**), the membrane-stable MinD state; and *c*_*e*_ (black) is the concentration of membrane-bound MinE dimers (**e**). We note that the color scheme in this figure differs from the previous ones. So, for example, because the concentration of membrane-stable MinD dimers is twice the value of *c*_*ded*_, the total membrane MinD monomer concentration shown in Fig 3 is 2(*c*_*d*_ + *c*_*de*_ + 2*c*_*ded*_). One important feature seen in both plots is the progression from green to blue to red to black, which emphasizes the ordered dynamics.

State values from the fits of the AABSM to the time-course data are shown in Fig 4. When concentrations of membrane-bound MinD dimers (green curve) are high, MinE is predominantly bound by two MinD dimers (blue curve) and MinD is stable on the membrane. As the concentration of membrane-bound MinD dimers decreases, the predominant form of MinE is bound to a single MinD dimer (red curve) and hence MinD is unstable on the membrane. Additionally, only when concentrations of membrane-bound MinD dimers become negligible (at about 200 s in the oscillation data and about 24 s in the MinD dissociation data with MinE in the flowed buffer) do the concentrations of membrane-bound MinE dimers (black curve) become non-negligible and persist. This progression of membrane-bound states is described in more detail in the Discussion.

### Removed-reaction fits of the AABSM

Without direct experimental evidence for the set of reactions that should comprise the AABSM, we included a large range of possible reactions in it. To clarify which reactions are important for describing the time-course data, we individually removed each non-necessary reaction from the AABSM and determined how well the resulting model could fit the time-course data. From a large decrease in the quality of fit we infer that the removed reaction is important, whereas a negligible decrease suggests that the removed reaction is either unimportant in describing the time-course data or its effect can be compensated for by altering other parameters in the model. See Table 2 for the influence of each removed reaction on the AABSM’s *χ*^2^ value and AIC score.

**Table 2:** A single-reaction knock-out study for the AABSM. We removed each listed reaction in turn from the AABSM and re-optimized the parameter values to get the best fit to the time-course data. For each resulting model, *χ*^2^ is the weighted sum of squared residuals, and AIC is the Akaike Information Criterion. $\chi_{\emptyset }^{2}$ and AIC_∅_ are the weighted sum of squared residuals and the AIC value for the AABSM with all reactions included. Greater values of $\chi^{2}/\chi_{\emptyset }^{2}$ and AIC − AIC_∅_ correspond to a larger decrease in the quality of the fit. The reactions are grouped with related reactions and ordered by their AIC difference from the complete AABSM for the oscillation data. See the caption of Table 1 for a discussion of AIC scale.

	Oscillation Data		MinD Dissociation Data	
Removed reaction	$\chi^{2}/\chi_{\emptyset }^{2}$	AIC − AIC_∅_	$\chi^{2}/\chi_{\emptyset }^{2}$	AIC − AIC_∅_
**ded+e** → **de+de**	4.8	510	1.0	−1.5
**de** → **D+E**	4.2	480	1.5	130
**de** → **d+e**	1.0	4.6	1.0	−2.0
**de** → **D+e**	1.0	−6.0	1.0	8.0
**d+de** → **ded**	4.0	460	1.2	64
$D\overset{+de}{\to }d$	3.5	410	NA	NA
$D\overset{+ded}{\to }d$	2.0	240	NA	NA
$D\overset{+d}{\to }d$	1.8	200	NA	NA
$E+d\overset{+e}{\to }de$	2.7	330	1.0	9.8
$E+d\overset{+ded}{\to }de$	1.0	3.1	1.0	−0.21
$E+d\overset{+de}{\to }de$	1.0	0.23	1.0	−2.2
**de+de** → **ded+e**	1.5	140	1.1	33
**d+e** → **de**	1.3	75	1.1	32
$E+ded\overset{+e}{\to }de+de$	1.2	49	1.1	15
$E+ded\overset{+ded}{\to }de+de$	1.2	48	1.1	18
**E+ded** → **de+de**	1.0	−1.2	1.0	1.7
$E+ded\overset{+de}{\to }de+de$	1.0	−0.17	1.1	23
**ded** → **d+de**	1.0	−1.3	1.1	19

As seen in Table 2, the reactions **ded+e** → **de+de** and $E+d\overset{+e}{\to }de$ are critical for the AABSM to recapitulate the oscillation data well. Although the table indicates other important reactions, we highlight these two because they suggest previously unknown functions of MinE dimers and for their role in the fundamental mechanism elaborated in the discussion section.

Additionally, we note that overall the AABSM is far more sensitive to reaction removal with the oscillation data than with the MinD dissociation data.

### Simulations of spatiotemporal patterning with the AABSM

**Fig 5:**
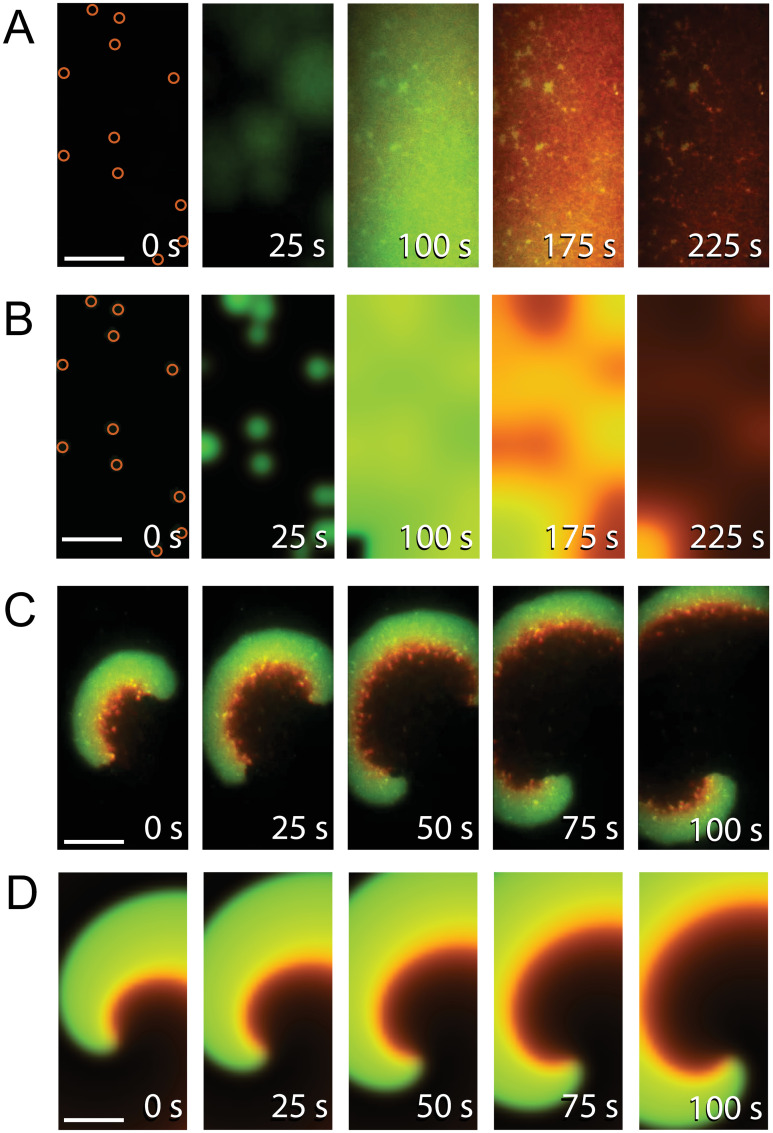
Simulations showing that the RD-AABSM recapitulates the nearly spatially-homogeneous oscillations and spiral waves seen in experiments using reaction parameters from the fit of the AABSM to the oscillation data (see S6 Table) and diffusion coefficients selected from a range of experimentally measured values (see Section 7 of the S1 Text). A: Frames from Ivanov and Mizuuchi’s Movie S2 [20] showing eleven initiation zones (marked with orange circles in the first panel) quickly filling the membrane with MinD, leading to nearly spatially-homogeneous oscillations; only the first cycle is shown. B: A simulation of the RD-AABSM starting with the same number and locations of initiation zones as identified in Panel A. Fewer initiation zones result in spatially inhomogeneous dynamics (see S14 Fig). C: Frames from later in Ivanov and Mizuuchi’s Movie S2 showing a double spiral wave. D: A simulation of the RD-AABSM showing a single spiral wave of similar form. Time stamps in each panel are elapsed time from the first frame. The scale bar shown in the first frame of each panel is 20 *μ*m. Implementation of the simulation in B is identical to that of those in S14 Fig except that the number and locations of initiation zones differ and the width is halved to match the experimental images. Images in D are cropped from the simulation shown in S16 Fig to match the dimensions of the experimental images.

In Ivanov and Mizuuchi’s experiments, small initiation zones of MinD arise seemingly randomly in space, grow outwardly, fuse, become almost spatially homogeneous (see Fig 5A here and Fig 1A of [20]), and finally wane under the influence of MinE. This repeats, generating oscillations. The number of initiation zones at the start of each oscillation decreases with time, and eventually the nearly spatially-homogeneous oscillations give way to traveling waves (see Movie S1 of [20]). We extended the AABSM, with reaction parameters from its fit to the oscillation data, into a reaction-diffusion system (RD-AABSM) with diffusion coefficients selected from a range of experimentally measured values. We also measured a number of initiation zones in Ivanov and Mizuuchi’s data to estimate the mean size of an initiation zone and the mean density of MinD in them. Using these estimates, we simulated the RD-AABSM with several different initial conditions varying in the number of small discs containing an elevated concentration of MinD that mimic the appearance of observed initiation zones. We found that, as observed in Ivanov and Mizuuchi’s experiments, nearly spatially-homogenous oscillations formed with a larger number of initiation zones. Furthermore, spatial inhomogeneity in the oscillations increased as the number of initiation zones decreased. For an even smaller number of initiation zones, traveling waves emerged. We interpret this result as an explanation for the temporal progression in the experiments from oscillations to travelling waves with successive Min-attachment cycles where the implied assumption is that the number of initiation zones decreases with each cycle. A comparison of an experimentally observed nearly spatially-homogeneous oscillation with a simulation starting from an initial condition with the same number and locations of initiation zones is shown in Fig 5A and 5B. A more complete analysis is presented in S14 Fig.

Ivanov and Mizuuchi also observed spiral waves in their experiments (see Fig 1B and Movie S2 of [20]), which presumably arose from spontaneous random breaks in symmetry in a traveling wave. We found in simulations of the RD-AABSM that artificially breaking symmetry in a traveling wave can lead to the formation of spiral waves (see Fig 5D for an example and more in S16 Fig). (To break symmetry, we set the state in half of the domain to the “resting state” while leaving a fully developed traveling-wave solution on the other half.)

Oscillations transforming into traveling waves and spiral waves were the only patterns observed in Ivanov and Mizuuchi’s experiments using the same membrane composition as in the experiments of the oscillation data. All other patterns were found using different membrane compositions. For this reason, we do not attempt to simulate those patterns, nor other types of patterns from other experiments with different experimental conditions, with the parameters estimated from the oscillation data. Details of the RD-AABSM and its simulations are described in Section 7 of the S1 Text.

Recapitulating the spatiotemporal patterning seen in Ivanov and Mizuuchi’s experiments is not unique to the AABSM. In simulations similar to those of the RD-AABSM, we find that a reaction-diffusion form of the AAM, the RD-AAM, also exhibits the transition from nearly spatially-homogeneous oscillations to a traveling wave and the formation of a spiral wave, as shown in S11 and S15 Figs. Simulations of the RD-AABSM bear a somewhat greater resemblance to experimental observations than do simulations of the RD-AAM: with the same number and location of initiation zones, RD-AABSM oscillations are more spatially homogeneous than RD-AAM oscillations (compare S14 and S11 Figs with Fig 5A here and Fig 1A and Movie S1 of [20]); RD-AABSM traveling-wave and spiral-wave speeds of roughly 0.34 *μm* *s*^−1^ and 0.29 *μm* *s*^−1^ are slightly more consistent with the wave speeds of 0.4 − 0.7 *μm* *s*^−1^ measured by Ivanov and Mizuuchi than RD-AAM traveling-wave and spiral-wave speeds of roughly 0.17 *μm* *s*^−1^ and 0.30 *μm* *s*^−1^; and the RD-AABSM spiral wave is more diffuse than the relatively tight RD-AAM spiral wave (compare S16 Fig and S15 Fig with Fig 5C here and Fig 1B and Movie S2 of [20]). These differences, however, should not be taken as strong evidence in favor of the AABSM over the AAM. In fact, for the reaction-diffusion versions of the CAAM (RD-CAAM) and the SAM (RD-SAM), we were incapable of triggering traveling waves and thus spiral waves when using the optimal reaction parameters in simulations like those described above for the RD-AABSM and the RD-AAM. Furthermore, the SAM did not produce the nearly spatially-homogeneous oscillations despite fitting the oscillation data fairly well. See S12 Fig, S13 Fig and Section 7 of the S1 Text for details. We expect that the presence or absence of waves for any model and the details of wave speed and wavelength are sensitive to parameter variation. For example, the CAAM reduces to the AAM when certain parameters are set to zero, so some parameterizations of the CAAM clearly support waves. In fact, this shortcoming of qualitative comparison of models to data that has been common in the Min modelling literature was precisely the motivation for the more quantitative analysis presented in the previous sections. A more intensive study of the parameter sensitivity (e.g. a numerical bifurcation analysis) of the RD models would be necessary to draw any firm conclusions about the suitability of the spatial models.

## Discussion

Over the last thirteen years, the success of reconstituting the Min protein interactions on supported membranes has allowed for new insights into the behavior of the system [20–25, 56, 57, 63–65]. Some of these experimental studies have been accompanied by mathematical modelling work, but none of these modelling studies nor others have attempted validation against time courses of protein concentration, instead focusing on qualitative features (e.g. pattern type, bifurcations, and transitions from stochastic to deterministic dynamics) and macroscopic quantification (e.g. oscillation period, wavelength, and wave velocity) [12, 21, 34, 51]. We formulated a biochemically novel model and used model selection, taking advantage of recent high quality quantitative time-course data [20, 24], to evaluate it against other recent models. Our model performed significantly better than the others, providing excellent fits. It is also consistent with other recent biochemical observations, sheds lights on the functional roles of MinE domains, and provides insight into factors that control the underlying dynamics of Min-system patterning, as discussed below.

### A free-MinD controlled stability-switching dynamic underlying patterning

Throughout the description below, our claims are based on a combination of the model comparisons, the time-course of state values in the AABSM shown in Fig 4, and the removed-reaction results in Table 2.

The biochemistry of the AABSM has the membrane stability of three forms of the MinD-membrane bond at its core. The least membrane-stable form is the MinD-MinE (**de**) complex. The most stable is the MinD-MinE-MinD (**ded**) complex. Intermediate to these two is the MinD dimer on its own (**d**).

**Fig 6:**
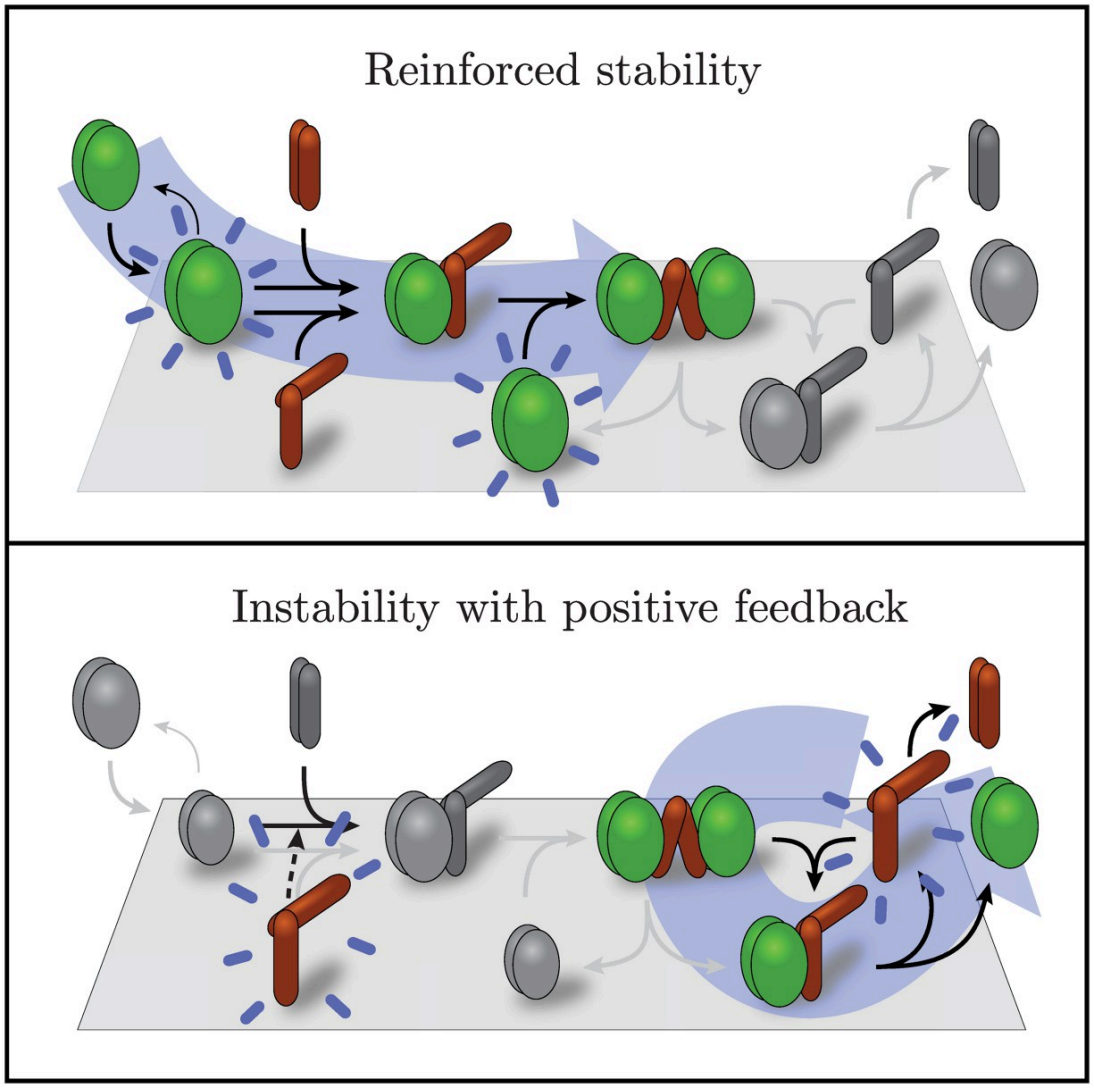
The membrane cycling of the Min proteins can be separated into two phases. In the first phase (reinforced stability), MinD attaches in greater numbers than MinE, and all forms of MinE are shuttled into the membrane-stable **ded** form (blue arrow in the top panel). The stability is reinforced by the resultant depletion of **e**, preventing the destabilizing feedback that happens in the second phase (instability with positive feedback). In the second phase, membrane saturation prevents further MinD attachment, allowing MinE recruitment to catch up. Two forms of positive feedback accelerate the removal of MinD—**e** breaks the membrane-stable **ded** into two membrane-unstable **de**’s which subsequently leave more **e** on the membrane as MinD is removed (blue arrow in bottom panel), and **e** recruits additional MinE from the bulk to bind to **d** forming **de** (dashed line) which, again, thereafter deposits more **e** on the membrane as MinD dissociates. In both panels, the state that drives the phase’s behavior is highlighted with a blue aura, and the relative concentration of **d**, which dictates overall stability, is shown by size.

The dynamics of the model start with a wave of MinD dimer attachment to the membrane ($D\overset{+\emptyset ,d,de,ded}{\to }d$ [see reactions in Table 2], green upstroke [see colored curves in Fig 4a]). This precedes and coincides with MinE recruitment and **de** complex formation (**d+E** → **de**, the first small upstroke in red). While the MinD dimer concentration is high, **de** complexes are quickly sequestered into the stable **ded** complex (**d+de** → **ded**, the drop in red accompanied by the upstroke and subsequent plateau in blue). This **ded**-complex plateau is much like a short-lived quasi-stable state seen in many excitable media. During this quasi-stable period, the flow of the **ded** complex into the **de** state, which is important later in the cycle, is suppressed by the initial sequestration of MinE in the **ded** state (**2d+e → d+de** → **ded**). This MinE-reinforced stability is illustrated in the top panel of Fig 6.

The next important transition occurs when new **d** recruitment is prevented by membrane saturation and MinE recruitment catches up. This change in the overall MinD-to-MinE ratio tips the balance from sequestration (**d+de** → **ded**) to MinD removal (**ded → d+de** → **d+D+e**), leaving some of the MinE dimer behind. The additional **e** from the removal step accelerates the previously suppressed desequestration (**ded+e** → **de+de**) to a flood (precipitous drop in blue as red spikes). This combined with the reaction $d+E\overset{+e}{\to }de$ pushes the membrane-stable MinD states into the **de** state, with subsequent MinD removal. The flood of **de** complexes is only possible once the MinD dimer supply is low and hydrolysis provides a supply of membrane-bound MinE. Thus the MinD-to-MinE ratio acts as a control parameter that dictates whether the membrane-stable **ded** complex or the membrane-unstable **de** complex is the dominant form of MinD. This positive feedback of MinE destabilizing MinD is illustrated in the bottom panel of Fig 6.

In contrast with previous models, here we have delved further into the unknown dynamics of the experimentally hidden states and used model selection to sort out the most likely progression through those states. The idea that MinD moves into a meta-stable state (**ded**), reinforced by inhibition of the backward reaction (sequestering), followed by destabilization under positive feedback is a refinement of the simple “D attaches, E removes” narrative that makes strong falsifiable predictions about the underlying biochemistry.

### Biochemical observations supporting the AABSM over the SAM

Evidence to support the AABSM’s membrane-stability assumptions comes from a few sources. It has been long known that MinE can induce hydrolysis of ATP by the MinD dimer [28]. The AABSM adheres to the conventional modelling interpretation that it is the MinD-MinE (**de**) complex that is responsible for the induction of hydrolysis, given that a single MinE dimer bound to a MinD dimer is capable of inducing hydrolysis in both subunits of the MinD dimer [58]. This latter observation is at odds with the membrane-stability assumptions of the SAM which has the **de** complex as stable on the membrane.

The assumption of reduced hydrolysis in the MinD-MinE-MinD (**ded**) complex is more speculative but is nonetheless supported by experiments. Vecchiarelli *et al*. showed that the MinD ATPase rate as a function of MinE concentration is sigmoidal and that the deletion of the dimerization domain of MinE removes the sigmoidal nature of the curve [24]. The sigmoidal MinE dependence is consistent with both the SAM and the AABSM. The loss of that sigmoidal dependence with the deletion is what the AABSM would predict, but the SAM would require some additional assumptions about the role of the second MinE subunit to be consistent with the loss.

In the SAM and the AABSM, a high concentration of MinE under mass action would leave negligible MinD on the membrane in the unbound dimer form, **d**, and would push all MinD into the model’s respective membrane-unstable state, **ede** for the SAM or **de** for the AABSM. Apart from forming complexes with MinD on the membrane, some MinE may also be present in the **e** state in both models. As such, a high concentration of MinE should push the ratio of MinD to MinE on the membrane to be no greater than 1/2 for the SAM and no greater than 1 for the AABSM. In a Min-system assay using a non-hydrolyzable ATP analogue testing precisely this, the MinD-to-MinE ratio on bicelles was found to be 1 [5], more in line with the prediction of the AABSM than the SAM.

### MinE membrane-binding and dimerization

Our results shed light on the critical but unknown role of MinE-membrane binding. One interpretation is that MinE-membrane binding allows MinE to remain on the membrane more persistently, permitting a MinE dimer to stimulate ATPase activity in multiple MinD dimers before dissociating from the membrane [30, 58]. The AABSM and removed-reaction fits suggest additional roles via the membrane-bound MinE state (**e**) in the reactions $E+d\overset{+e}{\to }de$ and **e+ded** → **2de**. On a supported-lipid-bilayer *in vitro*, the MinE-membrane-binding-deficient mutant MinE C1 [32] together with MinD formed a stationary structure in contrast to the traveling wave seen with wildtype MinE [22]. The traveling and stationary wave forms are similar except that the MinE C1 profile is rounded in comparison to the sharp peak in wildtype MinE [22]. Drawing on our findings, the lack of a sharp peak in the MinE C1 profile could follow from the inability of MinE C1 to recruit bulk MinE C1 to bind to membrane-bound MinD (the first reaction above), and the formation of a stable, stationary MinD/MinE C1 structure on the supported lipid bilayer could follow from the absence of the destabilizing second reaction which would be disabled by the MinE C1 mutant.

MinE dimerization is required for patterning both *in vivo* [66] and *in vitro* [24] but its role is not well understood. In the AABSM, MinE dimerization is critical for the sequestration of MinD in the stable **ded** state. Without it, a (strained) MinE dimer at the center of the complex would not be able to bridge two MinD dimers. Given that the **ded** state lies at the core of the AABSM, its removal through the elimination of MinE dimerization would presumably distrupt patterning both *in vivo* and *in vitro*, although the details of exactly how it would be disturbed are not clear. More concretely, a knock-out of the **ded** state provides an explanation for the observed loss of MinD stabilization in MinD-dissociation experiments when a dimerization-deficient MinE mutant is substituted for wildtype MinE (compare [24] Fig 6B first and third panels).

MinE membrane-binding and dimerization are integral to opposing processes in our supported model, the former required for destabilizing MinD and the latter required for stabilizing it. As such, through quantitative time-series model fitting, we have elucidated critical and complementary roles of MinE’s membrane-binding and dimerization domains.

### Model selection and redundant parameters

The AABSM has more parameters than other recently proposed models (e.g. [34, 51]), and so an improved fit over these is not surprising. In fact, as demonstrated by the removed-reaction study, some parameters are clearly redundant. We included them nonetheless to allow for an analysis that highlights which reactions are most important, and used the AIC score to account for this complexity advantage. We note that the SAM has even more parameters than the AABSM but did not fit the data as well, as measured by the *χ*^2^ value. This suggests to us that the AABSM is not just an improvement over earlier models by virtue of additional parameters but because some of those parameters represent reactions that better reflect the underlying biochemistry.

Both the SAM and the AABSM include many parameters and these show some redundancy in fitting the data, suggesting that we can dispense of some reactions. However, we nonetheless included this wide range of possible reactions in both models to give them both a fair chance at fitting the data—in a sense the models are maximally parameterized. We are interested in understanding the appropriateness of each proposed reaction mechanism (SAM vs AABSM) broadly defined and wanted to ensure that neither underperformed their potential by an inadvertent omission of potentially important reactions. In other words, we wanted to compare the two conceptual frameworks for understanding the Min system rather than just a particular manifestation of each one.

### Robustness of results and modelling uncertainty

Density calibration of the time-course data is subject to inherent experimental and methodological error. To ensure that our results supporting the AABSM over the SAM are not just a byproduct of our specific estimates, we perturbed our estimates over a reasonable range of values (±0, 10, 20%) and fit the SAM and the AABSM to the perturbed time-course data. We found that the AABSM outperformed the SAM in fitting all the perturbed data sets, demonstrating the robustness of our results. Details are shown in S2 Table.

The oscillation-data and the MinD-dissociation-data experiments were carried out with different membrane compositions and buffer flow rates. We expected parameter values to vary somewhat between the two data sets, so we estimated parameters for each experiment independently. As shown in S10 Fig, when we instead fit the two data sets simultaneously, constraining the parameters used for each experiment to be within roughly an order of magnitude of each other, we find that the AABSM can still fit both sets of time-course data nearly as well. The parameter values from this approach are shown in S8 Table. We also find that the AABSM outperforms the SAM in fitting the two sets of time-course data simultaneously under all tested constraints between parameters in the two experiments, as shown in S10 Fig.

In the removed-reaction study, we found that the removal of some parameters did not impact the quality of the fit significantly after re-optimization of the remaining parameters. In all such cases, when the AIC score dropped for one of the data sets, it rose in the other, indicating that the AABSM fitting has some degrees of freedom that might be eliminated when both data sets are fit simultaneously with the same parameter values for the two experiments. Instead of imposing this strict constraint, which would likely be incorrect given the variability in the experimental conditions, we point out that S10 Fig shows fits for a continuum of progressively looser constraints between the parameters in the two experiments.

Variability in optimal parameter values both across fits of the AABSM model and its removed-reaction simplifications and across the data sets serves as a warning about the biological accuracy of our parameter values. This combined with the degrees of freedom in the AABSM fitting made it difficult to meaningfully test some of the hypotheses described above through simulation, including the roles of membrane binding and dimerization.

Ultimately, simplifying the AABSM and accurately estimating its parameters is beyond the scope of what we can do with the time-course data we have and will require the incorporation of more data, experimental evidence, and additional theoretical considerations. Fitting the AABSM to a larger data set of spatially homogeneous dynamics, over a broad range of constant bulk MinD and MinE concentrations, could allow relevant reactions in the model to be more readily identified and parameters in the model to be estimated with greater accuracy. Additionally, the incorporation of data from FRET experiments which are able to distinguish hidden states in the system would provide refinement in the fitting beyond that of just the total MinD and total MinE near the membrane, allowing for greater precision in hypothesis testing and parameter estimation. Once the AABSM is well characterized, analysis and simulation of it may reveal details of the mechanism driving spatiotemporal patterning in the Min system and a means to predict conditions under which novel patterns may emerge.

## Methods

### Data

To fit the models, we use *in vitro* data, kindly provided by Ivanov and Mizuuchi [20] (oscillation data) and Vecchiarelli *et al*. [24] (MinD dissociation data). The geometry, space scale, and more deterministic behavior of the *in vitro* assays allows improved opportunities for analysis in comparison with *in vivo* data. Due to these advantages in combination with buffer flow in the experiments that ensures a constant concentration of components in the bulk and the near spatial-homogeneity of the data, the data fitting problem was reduced from one of fitting each of the PDE models to one of fitting the corresponding ODEs, each with three fewer states (eliminating dynamic variables for concentrations of bulk MinD monomers, bulk MinD dimers, and bulk MinE dimers). In other words, we focused on fitting the reaction kinetics without the complications of spatial dynamics and variable bulk states. To be certain that our assumption of constant bulk states is a reasonable approximation, beyond the arguments for this made by Ivanov and Mizuuchi, we simulated dynamics in the flow cell during their experiments to assess their claim in more quantitative detail. We found that bulk protein concentrations vary by only a small amount (<4%) throughout the experiment, supporting our model reduction of bulk states to constants. Details are shown in Section 1 of the S1 Text.

Some preprocessing of Ivanov and Mizuuchi’s data was required. Fluorescence intensities of fluorescently labeled MinD and MinE were not spatially aligned, so we used cross-correlation to align the images, taking advantage of similarly shaped structures in the two signals. To remove background noise from the aligned data, we used images from before any protein reached the imaging region of the flow cell to calculate an average noise intensity over space and time and subtracted that from all subsequent images. Then we flattened the shifted aligned data to correct for illumination vignetting by multiplying by the reciprocal of an optimally chosen Guassian that we normalized to have a maximum value of 1. The conversion factor from MinE fluorescence intensity to MinE density was not experimentally determined. Using flattened MinD and MinE fluorescent intensities before a significant amount of either protein bound to the supported lipid bilayer (with a slight correction for a small but increasing amount of bound MinD), known buffer concentrations of MinD and MinE, and the depth of the evanescent waves in TIRF microscopy, we calculated conversions from flattened MinD and MinE fluorescent intensities to MinD and MinE molecule densities. We found our conversion for MinD to be in fairly good agreement with Ivanov and Mizuuchi’s estimate.

To extract a time series from Ivanov and Mizuuchi’s data that best represents reaction kinetics only, we looked for a region in the images that was as close to spatially homogeneous as possible, avoiding, for example, regions and time intervals with traveling wave fronts moving through them. To do so, we searched over all disks of 1000 pixels in the preprocessed data for the disk with the least spatial variation in MinD and MinE densities during a single period of the oscillation. To reduce pixel-to-pixel noise in measuring this spatial variation, we smoothed the preprocessed data using values from local, radially-symmetric 2-D linear regressions over disks of 100 pixels. S1 Movie illustrates our approach, and for comparison S2 Movie shows the same analysis applied to data when traveling waves move through the frame. Ultimately, for the model fitting, we used mean values of non-smoothed MinD and MinE densities at each time within the chosen disk as the time series for model fitting. The resulting oscillation data is shown in the top panel of S2 Fig and in normalized form in Panel A of both Figs 2 and 3 (dotted). SEMs were omitted because they were indistinguishable by eye from the means.

Vecchiarelli *et al*.’s data consisted of MinD and MinE time series, both normalized by the concentration of MinD at the beginning of dissociation, and raw MinD time-series data. To calibrate the time-series data, we first aligned normalized MinD data and raw MinD data under best-fitting affine transformations. Once aligned, scalings in each of the best-fitting affine transformations provided us with conversions from normalized units to the arbitrary units (AU) used in the experiments. We calibrated normalized MinD and MinE time-series data for model fitting using the conversion from AU to dimers/*μm*^2^ reported by Vecchiarelli *et al*. The resulting MinD dissociation data is shown in the bottom panel of S2 Fig and in normalized form in Panel B of both Figs 2 and 3 (dotted). We note that, unlike the oscillation data which we calibrated directly from data earlier in the same experiment, Vecchiarelli *et al*. calibrated MinD dissociation data using data from a different experiment of their own with a similar setup, so MinD dissociation data may contain more calibration error than the oscillation data.

### Models

The reactions included in each of the models are displayed in Fig 1. Below, we describe the modifications made to the Bonny Model [34] as well as the relationships between each of the tested models and briefly discuss reasons for including or omitting some reactions in the models.

For consistency with buffer-flow experimental conditions, we fixed the bulk concentrations of MinD and MinE (see Section 1 of the S1 Text and the Supporting Information Materials and Methods from [20] for justification). The original Bonny Model has no spontaneous MinD dissociation which is critical for fitting Vecchiarelli’s MinD dissociation data with MinE absent, so we included reaction **d** → **D** in the Asymmetric Activation Model. The form of the term we used for this reaction (not mass action) is described and justified in the S1 Text.

Because the SAM and AABSM are more complicated models than the AAM, aiming to explain the more recent experimental results that the AAM cannot account for, we also included the CAAM in our model selection process. The CAAM essentially includes the intersection of all reaction terms found in the SAM and AABSM, giving it more scope to account for some of those more recent observations (see the S1 Text for more discussion). This additional scope does not allow it to account for the dual role of MinE which is why we tested the SAM and AABSM.

We included a wide range of possible reactions in the SAM and the AABSM. We added all backward reactions except those where MinE dimers spontaneously dissociate from complexes with MinD dimers on the membrane and return to the bulk (we added these to the CAAM as well). Those excluded backward reactions were omitted because residence times of MinE dimers are at least 1.3 times as long as residence times of MinD dimers in all portions of Min-protein traveling waves on a supported lipid bilayer *in vitro* [22]. We also excluded reactions where MinD dimers in **de** complexes return to the bulk in the SAM and where MinD dimers in **ded** complexes return to the bulk in the AABSM, to enforce stability in membrane-stable MinD states. As shown in S3 Table, including MinD-membrane dissociation reactions for membrane-stable MinD states in the SAM and AABSM does not change our model selection results.

MinE undergoes a conformational change from a latent 6 *β*- stranded form to a active 4 *β*- stranded form before it can bind to MinD [30, 67]. This conformational change is thought to be MinD dependent [30, 67], so we pack it into reactions where bulk MinE binds to membrane-bound MinD, rather than modeling it explicitly at the cost of requiring more states and parameters in the models. It has been proposed that after stimulating ATPase activity in MinD and releasing from the membrane, MinE retains its active form long enough for it to bind to membrane-bound MinD before reverting to its latent form, and in this way, it forms a thin layer near the membrane that repeatedly binds to and stimulates ATPase activity in MinD [56]. However, the rate of cytosolic MinE reversion from an active 4 *β*- stranded form to an inactive 6 *β*- stranded form has been measured to be very fast, over 1000 *s*^−1^, for MinE from *N. gonorrhoea* [68]. Additionally, if a layer of active MinE were having an effect on MinD-MinE dynamics during the oscillations in Ivanov and Mizuuchi’s experiments, then following local MinE accumulation and MinD depletion on the membrane, fast buffer flow would cause greater MinE accumulation and MinD depletion in the direction downstream of the flow than in the directions upstream of it and perpendicular to it. This behavior is not observed, so we do not include this type of reaction in the models of their data.

### Parameter fitting

To find optimal data-fitting numerical solutions for our models, we fit them to the oscillation data and the MinD dissociation data using the homotopy-minimization method for parameter estimation in differential equations [59]. Apart from the constrained optimizations shown in S8 Table, we fit models to the two sets of time-course data independently. Details of data fitting are described in the S1 Text.

The oscillation data, dissociation data, and matlab scripts that simulate the AABSM in both contexts with optimal parameters are available at https://github.com/ecytryn/Min-biochemistry.

## Supporting information

S1 Text Supporting Information text with more details on the methods, models, and results. (PDF)

S1 FigThe simulated spatial concentrations of MinD and MinE.The simulated concentrations of MinD and MinE (*c*_*D*_ and *c*_*E*_) inside a flow cell with in-phase oscillation of MinD and MinE on both the top and bottom of the flow cell, with a temporal offset in space in the oscillations as in the data from which the oscillation data was generated. The numerical solutions to Eq. S1 are shown in (a) and (b) long after having converged to a propagating wave solution that is periodic on the domain shown and moves to the right, at 507 simulated seconds, the time passed during the oscillation data. The vertical scale (*y*) is expanded relative to the horizontal scale by a factor of ∼20 for the sake of legibility. Yellow shows where the Min proteins are slightly depleted from the bulk at the leading edge of the wave and darker green/red shows slight enrichment in its wake. *c*_*D*_ and *c*_*E*_ do not vary much from $\bar{c_{D}}=638.3\;{\mu m}^{-3}$ and $\bar{c_{E}}=819.0\;{\mu m}^{-3}$ because the rapid rate of flow inside the flow cell acts to homogenize the concentrations of MinD and MinE. This is in sharp contrast to *c*_*D*_ and *c*_*E*_ shown in (c) and (d) from an identical simulation except without flow, $\bar{v}=0\;\mu m\;s^{-1}$. Note the dramatic difference in the color scales between (a)/(b) and (c)/(d).(PNG)

S2 Fig Oscillation and MinD dissociation data. The oscillation data is shown in Panel A, and MinD dissociation data is shown in Panel B. Densities shown are the total concentrations of MinD and MinE, monomers *μm*^−2^. SEMs in the data are omitted because they would be indistinguishable from the means shown by eye.
(PDF)

S3 Fig The CAAM fit to the data. For the CAAM, the fit to the oscillation data is shown in Panel A, and the fit to the MinD dissociation data is shown in Panel B. Fits are solid, and the data are dotted.
(PDF)

S4 Fig The SAM fit to the data. For the SAM, the fit to the oscillation data is shown in Panel A, and the fit to the MinD dissociation data is shown in Panel B. Fits are solid, and the data are dotted.
(PDF)

S5 Fig The FHNM fit to the data. For the FHNM, the fit to the oscillation data is shown in Panel A, and the fit to the MinD dissociation data is shown in Panel B. Fits are solid, and the data are dotted.
(PDF)

S6 Fig State values for the AAM fits. For the AAM, state values from the fit to the oscillation data are shown in (a), and state values from the fit to the MinD dissociation data with MinE in the flowed buffer are shown in (b).
(PNG)

S7 Fig State values for the CAAM fits. For the CAAM, state values from the fit to the oscillation data are shown in (a), and state values from the fit to the MinD dissociation data with MinE in the flowed buffer are shown in (b).
(PNG)

S8 Fig State values for the SAM fits. For the SAM, state values from the fit to the oscillation data are shown in (a), and state values from the fit to the MinD dissociation data with MinE in the flowed buffer are shown in (b).
(PNG)

S9 Fig State values for the FHNM fits. For the FHNM, state values from the fit to the oscillation data are shown in (a), and state values from the fit to the MinD dissociation data with MinE in the flowed buffer are shown in (b).
(PNG)

S10 FigThe simultaneous fitting of both data sets with parameter constraints.For each parameter *p* that is nontrivial in both the model of the oscillation data and the model of the MinD dissociation data with MinE in the flowed buffer, except for constant-concentration parameters *C*_*d*_, *C*_*e*_, and $c_{\bar{d}}$, we constrain *p* in the fit of the oscillation data to be less than or equal to *γp* in the fit of the MinD dissociation data, and we constrain *p* in the fit of the MinD dissociation data to be less than or equal to *γp* in the fit of the oscillation data, for *γ* = 1, 2, 4, 8, 16. The resulting *χ*^2^ values from the fits, the weighted sums of squared residuals, are shown as dots. The *χ*^2^ values from unconstrained optimizations, in which *γ* = ∞, are shown as flat dashed lines. When parameters across the data sets are constrained to be within roughly an order of magnitude of each other, the models, most notably the AABSM, can still recapitulate both data sets almost as well as without constraints. In fitting, we take into account differences in buffer MinE concentrations—1.36*μM* for the oscillation data and 2.5*μM* for the MinD dissociation data with MinE in the flowed buffer—by multiplying rate parameters of bulk MinE binding reactions, $\omega_{E,d\to de}^{z}$ and $\omega_{E,de\to ede}^{z}$ for *z* ∈ {∅, *de*, *ede*, *e*} in the SAM and $\omega_{E,d\to de}^{z}$ and $\omega_{E,ded\to de,de}^{z}$ for *z* ∈ {∅, *de*, *ded*, *e*} in the AABSM, which otherwise have a multiplicative factor of *c*_*E*_ built into them, by 1.36*μM* or 2.5*μM*. This removes the multiplicative factor of *c*_*E*_ from the rate parameters of bulk MinE binding reactions and instead incorporates a conversion factor from bulk MinE monomers to dimers in them, under the assumption that all bulk MinE is stable in the dimer state. Parameter estimates for the AABSM with *γ* = 16 are shown in S8 Table.(PDF)

S11 FigThe transition from spatially near-homogenous oscillations to traveling waves with a decreasing number of initiation zones in simulations of the RD-AAM.Solutions to the RD-AAM with *N*_*i*_ = 1000, 100, 10, and 1 initiation zones at time *t* = 0 *s* are shown in Panels A, B, C, and D at the displayed times. The solution to the RD-AAM with *N*_*i*_ = 1000 at *t* = 0 *s*, *N*_*i*_ = 100 at *t* = 500 *s*, *N*_*i*_ = 10 at *t* = 1000 *s*, and *N*_*i*_ = 1 at *t* = 1500 *s* is essentially the same as the concatenation of Panels A-D through time but differs from that shown in Panel D in that the traveling wave from the final frame of Panel C continues to propagate (overriding the initiation zone in the first frame of Panel D). MinD and MinE are shown in green and red on the same scale in all frames except for the first frame of each panel. The first frame of each panel is shown with green and red intensity scales that are amplified by a factor of three so that initiation zones are visible. Each square shown is $85\frac{1}{3}\;\mu m\times 85\frac{1}{3}\;\mu m$, with a height of the microscopy images in Ivanov and Mizuuchi’s experiments.(PDF)

S12 Fig Spatially near-homogeneous oscillations in simulations of the RD-CAAM. Solutions to the RD-CAAM with *N*_*i*_ = 1000, 100, 10, and 1 initiation zones at time *t* = 0 *s* are shown in Panels A, B, C, and D at the displayed times. Between the second and third frames of Panels C and D, innate oscillations cause excitation outside of expanding initiation zones, suppressing the formation of outward growing traveling waves. Color and spatial scales are as described in S11 Fig.
(PDF)

S13 Fig MinD depletion in initiation zones followed by low-amplitude spatially-near-homogeneous oscillations in simulations of the RD-SAM. Solutions to the RD-SAM with *N*_*i*_ = 1000, 100, 10, and 1 initiation zones at time *t* = 0 *s* are shown in Panels A, B, C, and D at the displayed times. Initiation zones as implemented do not drive spatially near-homogeneous oscillations nor traveling-wave formation. Although it is hardly perceptible in Panel B, a low-amplitude oscillation occurs, with a peak around 700 *s*. Color and spatial scales are as described in S11 Fig.
(PDF)

S14 Fig The transition from spatially near-homogenous oscillations to traveling waves with a decreasing number of initiation zones in simulations of the RD-AABSM. Solutions to the RD-AABSM with *N*_*i*_ = 1000, 100, 10, and 1 initiation zones at time *t* = 0 *s* are shown in Panels A, B, C, and D at the displayed times. The solution to the RD-AABSM with *N*_*i*_ = 1000 at *t* = 0 *s*, *N*_*i*_ = 100 at *t* = 500 *s*, *N*_*i*_ = 10 at *t* = 1000 *s*, and *N*_*i*_ = 1 at *t* = 1500 *s* is essentially the same as shown in Panels A, B, C, and D. Color and spatial scales are as described in S11 Fig.
(PDF)

S15 Fig A spiral wave emerges from a break in symmetry in a traveling wave in a simulation of the RD-AAM. Starting from a single initiation point at (*x*, *y*) = (0, 0) *μm* at time *t* = 0 *s* with a radius of 2 ⋅ 2.40 *μm*, a traveling wave emerges (Panel A). Breaking the symmetry in the traveling wave (Panel B), a spiral wave emerges and persists, as shown in Panel C and roughly half a rotation later in Panel D. Simulation times are displayed, MinD and MinE are shown in green and red on the scale of MinD and MinE in the oscillation data, and the height of each square shown is twice that of the microscopy images in Ivanov and Mizuuchi’s experiments.
(PDF)

S16 Fig A spiral wave emerges from a break in symmetry in a traveling wave in a simulation of the RD-AABSM. Starting from a single initiation point at (*x*, *y*) = (0, 0) *μm* at time *t* = 0 *s* with a radius of 2.40 *μm*, a traveling wave emerges (Panel A). As in S15 Fig, breaking the symmetry in the traveling wave (Panel B), a spiral wave emerges and persists, as shown in Panel C and roughly half a rotation later in Panel D. Simulation times are displayed, and the color and spatial scales are as described in S15 Fig.
(PDF)

S1 TableModel Comparison with and without MinD-membrane-dissociation reactions of membrane-stable MinD states.An asterisk (*) indicates that the model includes MinD-membrane-dissociation reactions of the membrane-stable MinD state, the reactions de → D+E and de → D+e for the SAM and the reactions ded → D+E+D, ded → D+E+d, ded → D+de, ded → D+e+D, and ded → D+e+d for the AABSM. *χ*^2^ is the weighted sum of squared residuals, and AIC is the Akaike information criterion. $\chi_{\text{min}}^{2}$ is the minimum *χ*^2^ value amongst the models, and AIC_min_ is the minimum AIC value amongst the models. The AABSM without the inclusion of MinD-membrane-dissociation reactions of the membrane-stable MinD state fits the oscillation data and the MinD dissociation data better than the SAM, even with the inclusion of MinD-membrane-dissociation reactions of the membrane-stable MinD state in the SAM. Including MinD-membrane-dissociation reactions of the membrane-stable MinD state in the AABSM does not appreciably improve its fit to the oscillation data and does not improve its fit to the MinD dissociation data. We include this table as justification for the omission of the membrane-stable MinD dissociation reactions from the SAM and AABSM as mentioned in the Materials and Methods.(PDF)

S2 TableComparing the fits of the SAM and the AABSM to the time course data with perturbations.The time course data is subject to inherent experimental and methodological error in density calibration. As such, we perturb the time course data by a range of scaling factors and fit the SAM and the AABSM to the perturbed data sets to compare how well the models can fit the time course data over a range of density calibration values. When and only when MinD and MinE are rescaled in tandem, both the SAM and the AABSM (as well as the nested CAAM and AAM) can be rescaled to accommodate the change without affecting the quality of the fits to the data. I.e., the quality of the fits of the SAM and the AABSM to the time course data depend only on the relative density calibration of MinD and MinE, not the density calibration value of each. In accordance, we scale MinE time course data by *ϵ* = 0.8, 0.9, 1, 1.1, 1, 2, leave MinD time course data unchanged, and fit the SAM and the AABSM to the perturbed time course data—a fit of the models to the time course data with ±0, 10, 20% MinE-to-MinD relative density calibration error of that measured experimentally. Results are shown above, with *χ*^2^ and $\chi_{\text{min}}^{2}$ as defined in S1 Table. For both the oscillation data and the MinD dissociation data, the AABSM outperforms the SAM in fitting (as measured by *χ*^2^) for all density calibration perturbations (values of *ϵ*). A larger perturbation in density calibration from that measured experimentally (a larger deviation from *ϵ* = 1) results in a decrease in the quality of the fit of the AABSM to the time course data (a larger value of *χ*^2^), apart from a minor exception at *ϵ* = 1.2 for the MinD dissociation data.(PDF)

S3 Table Parameters from the fits of the AAM to the oscillation data and the MinD dissociation data. (PDF)

S4 Table Parameters from the fits of the CAAM to the oscillation data and the MinD dissociation data. (PDF)

S5 Table Parameters from the fits of the SAM to the oscillation data and the MinD dissociation data. (PDF)

S6 Table Parameters from the fits of the AABSM to the oscillation data and the MinD dissociation data. (PDF)

S7 Table Parameters from the fits of the FHNM to the oscillation data and the MinD dissociation data. (PDF)

S8 TableParameters from the simultaneous fitting of the AABSM to the oscillation data and the MinD dissociation data with parameter constraints.*γ* = 16 in the constrained optimization. We presume that these are the most biologically relevant parameter estimates we have. See the caption of S10 Fig for details of the constrained fit, notably regarding changes to the AABSM involving rate parameters $\omega_{E,d\to de}^{z}$ and $\omega_{E,ded\to de,de}^{z}$ for *z* ∈ {∅, *de*, *ded*, *e*}.(PDF)

S1 Movie A spatiotemporal analysis of the full MinD and MinE density profile from which the oscillation data was derived. Calibrated experimental data is shown on top of smoothed data in the top plots, and smoothed data is shown on top of the mean data in the disk in the bottom plots. Smoothing the data reveals the collective dynamics of the system beneath experimental noise. The mean value in the disk, which we used for the oscillation data, varies little from the smoothed data. It is difficult to see this in the video because they are so similar. Watching a case in which they differ more, like S2 Movie, makes it easier to see how close they are in this case. Details of selecting the data from which the oscillation data was derived and data smoothing are described in the Data section of the Methods.
(MP4)

S2 Movie A spatiotemporal analysis of the full MinD and MinE density profile showing a traveling wave. Calibrated experimental data is shown on top of smoothed data in the top plots, and smoothed data is shown on top of the mean data in the disk in the bottom plots. The mean value in the disk showed too much deviation from the smoothed data and hence was not selected for model fitting. The mean value in the disk differs most dramatically from the smoothed data when a traveling wave propagates through the disk.
(MP4)
