# Supplementary material for: The mechanism of MinD stability modulation by MinE in Min protein dynamics
Source: PLoS Comput Biol. 2023 Nov 17;19(11):e1011615. doi: 10.1371/journal.pcbi.1011615 (PMC10691731; doi:10.1371/journal.pcbi.1011615)
Supplement: S1 Text — (PDF) [file pcbi.1011615.s001.pdf]

Supporting Information for
The mechanism of MinD stability modulation by MinE in Min protein dynamics

William C Carlquist and Eric N Cytrynbaum

October 10, 2023

1 Simulating MinD and MinE Concentrations in the Flow Cell

Ivanov and Mizuuchi established that the concentrations of reaction components in their flow-cell experiments were roughly constant in space and time, via replenishment from solution that flows much faster than the speed of a traveling wave and Taylor dispersion that acts to mix the bulk on a much faster time scale than a traveling wave period [4]. Here, we simulate MinD and MinE concentrations in a flow cell to assess their claims in more quantitative detail. To do so, we use the oscillation data to define an in-phase oscillation on both the top and bottom of the flow cell with a temporal offset in space in the oscillations, τ, and simulate the behavior of bulk concentrations of MinD and MinE in the flow cell throughout. For the data from which we generated the oscillation data, the difference in the time between the initiation of oscillations on the left and right side of the microscopy image was no greater than about 42 s, over a distance of about 41 μm. Thus, for our simulation we use a value of $\tau = 1\ s\ \mu m^{-1}$. To simulate concentrations throughout the flow cell without a very large computational grid, we impose periodicity on our simulation domain. Altogether, this is equivalent to simulating bulk concentrations of MinD (c_D) and MinE (c_E) in the flow cell using the oscillation data to define the local dynamics of a periodic propagating wave that moves in parallel on both the top and bottom surfaces of a flow cell with velocity $w = \tau^{-1}$:

$$\frac{\partial c_D}{\partial t} = D_D \left(\frac{\partial^2 c_D}{\partial x^2} + \frac{\partial^2 c_D}{\partial y^2} \right) - v(y) \frac{\partial c_D}{\partial x}, \tag{S1a}$$

$$\frac{\partial c_E}{\partial t} = D_E \left(\frac{\partial^2 c_E}{\partial x^2} + \frac{\partial^2 c_E}{\partial y^2} \right) - v(y) \frac{\partial c_E}{\partial x}, \tag{S1b}$$

$$v(y) = \frac{3\bar{v}}{2} \left(1 - \left(\frac{2y - h}{h} \right)^2 \right), \tag{S1c}$$

with boundary and initial conditions

$$\begin{aligned} c_D(0,y,t) = c_D(L,y,t),\ D_D \frac{\partial c_D(x,0,t)}{\partial y} &= f_D' \left(t - \frac{x}{w} \right), \\ -D_D \frac{\partial c_D(x,h,t)}{\partial y} &= f_D' \left(t - \frac{x}{w} \right),\ c_D(x,y,0) = \overline{c_D}, \end{aligned} \tag{S1d}$$

$$\begin{aligned} c_E(0,y,t) = c_E(L,y,t),\ D_E \frac{\partial c_E(x,0,t)}{\partial y} &= f_E' \left(t - \frac{x}{w} \right), \\ -D_E \frac{\partial c_E(x,h,t)}{\partial y} &= f_E' \left(t - \frac{x}{w} \right),\ c_E(x,y,0) = \overline{c_E}, \end{aligned} \tag{S1e}$$

where t is the time; x and y are the distances along the flow cell length and height; D_D and D_E are the diffusion coefficients of c_D and c_E; $v(y)$ is the flow velocity (planar Poiseuille flow), a quadratic in y with a value of 0 at the bottom and top of the flow cell, where $y = 0$ and $y = h$, and an average value of $\bar{v}$; L is the wavelength of the periodic propagating wave, $w \times$ the time passed during the oscillation data; $f'_D(t)$ and $f'_E(t)$ are the periodic derivatives of MinD and MinE oscillation data, calculated numerically using centered differences at grid point with linear interpolation in between; and $\overline{c_D}$ and $\overline{c_E}$ are the concentrations of MinD and MinE in the solution when infused into the flow cell. To be consistent with Ivanov and Mizuuchi's experiment, $h = 25\ \mu m$, $\bar{v} = 500\ \mu m\ s^{-1}$, $\overline{c_D} = 638.3\ \mu m^{-3}$, and $\overline{c_E} = 819.0\ \mu m^{-3}$. Additionally, $D_D = D_E = 60\ \mu m^2\ s^{-1}$, roughly that as measured in [6] with a similar experimental setup to that of Ivanov and Mizuuchi. We solve Eq. S1 numerically by discretizing first and second order partial derivatives in space using first and second order central finite differences and calculating temporal updates using the implicit trapezoidal method, with grid points 0.5 μm apart in space and 0.5 s apart in time.

We solve Eq. S1 numerically and find that the solution converges to periodic propagating wave, as shown in Fig. S1, with $c_D/\overline{c_D} \in [0.961, 1.023]$ and $c_E/\overline{c_E} \in [0.995, 1.010]$. Reversing the direction of the offset, $\tau = -1\ s\ \mu m^{-1}$ ($w = -1\ \mu m\ s^{-1}$), does not appreciable change the ranges of c_D and c_E upon converging to a propagating wave solution. These results demonstrate that the concentrations of MinD and MinE do not vary much inside the flow cell under the simulated conditions. As can be seen by comparing simulations with and without flow in Fig. S1, this occurs because the rapid flow rate acts to homogenize concentrations in the bulk more quickly than the concentrations are augmented by reactions on the flow cell surfaces. Of all the patterns observed in Ivanov and Mizuuchi's experiments, spatially near-homogeneous oscillations are the most spatially synchronized and exhibit the largest changes in protein density. As such, homogenization effects should be greater and bulk variation should be less than those of spatially near-homogeneous oscillations for other patterns observed in Ivanov and Mizuuchi's experiments.

About 30 s after being injected into the flow cell, solution containing reaction components reaches the middle of the flow cell, the site of data acquisition. This rapid rate of replenishment compared to an oscillation period of over 500 s ensures that reaction components in the flow cell are not depleted in time. A strong homogenizing effect in the bulk of the flow cell from rapid flow, as the results from our simulations show, in conjunction with a relatively rapid renewal of reaction components from inflow, supports the claim that bulk components in the flow cell are roughly constant in space and time in Ivanov and Mizuuchi's experiments. As such, in our models of their experiment, we approximate concentrations of bulk reaction components as constants.

2 ODE Models

2.1 Model Notation

- c_d is the concentration of membrane-bound MinD dimers.
- c_{de} is the concentration of MinD-MinE hetero-tetramer complexes on the membrane.
- c_{ede} is the concentration of MinE-MinD-MinE hetro-hexamer complexes on the membrane.
- c_{ded} is the concentration of MinD-MinE-MinD hetero-hexamer complexes on the membrane.
- c_e is the concentration of membrane-bound MinE dimers.
- c_D is the constant concentration of bulk MinD dimers.
- c_E is the constant concentration of bulk MinE dimers.
- $\omega_{u,v\to x,y}$ denotes the reaction rate of c_u and c_v converting into c_x and c_y for $u, v, x, y \in \{\emptyset, D, E, d, de, ede, ede, e\}$.

- $\omega^z_{u,v\to x,y}$ denotes the reaction rate of c_u and c_v converting into c_x and c_y with facilitation by c_z for $u, v, x, y, z \in \{\emptyset, D, E, d, de, ede, ede, e\}$.

- $c_{\max}$ is the saturation concentration of MinD dimers on the membrane.

- c_s is the half-max concentration constant in the Hill equation modeling the rate of spontaneous MinD-membrane dissociation.

- n_s is the Hill coefficient in the Hill equation modeling the rate of spontaneous MinD-membrane dissociation.

- $c_{\bar{d}}$ is the constant concentration of MinD dimers that are persistently bound to the membrane. This accounts for the effect of MinD dimers that remain permanently bound to the membrane, as observed in experiments [4], on spontaneous MinD-membrane dissociation.

- C_d is the constant concentration of MinD monomers that are not accounted for in ODE models, the planar-density of constant bulk MinD monomers illuminated within the depth of the evanescence wave in TIRF microscopy and the concentration of MinD monomers that are persistently bound to the membrane.

- C_e is the constant concentration of MinE monomers that are not accounted for in ODE models, the planar-density of constant bulk MinE monomers illuminated within the depth of the evanescence wave in TIRF microscopy and the concentration of MinE monomers that are persistently bound to the membrane.

2.2 Modeling Spontaneous MinD Dissociation from the Membane

Without the inclusion of spontaneous MinD dissociation from the membrane in models, fitting MinD dissociation data with MinE absent would be meaningless. The residence time of MinD on the supported lipid bilayer increases from 11 s to at least 40.71 s as the concentration of MinD increases from 0.275 μM to 1.1 μM in the absence of MinE [5]. This concentration-dependent stabilization presumably occurs through the formation of some higher order MinD structure [7, 3] held together by relatively weak interactions between the MinD dimers in it [2] but is not well characterized. As such, we phenomenologically model the rate of spontaneous MinD-membrane dissociation by a reverse hill function,

$$\omega_{d\to D}\frac{c_s^{n_s}}{c_s^{n_s}+c_{\text{all }d}^{n_s}}c_d, \tag{S2}$$

where $\omega_{d\to D}$ is the maximum value of the off rate, c_s is the MinD dimer concentration on the membrane at the half-max value of the off rate, $c_{\text{all }d}$ is the concentration of all MinD dimers (in all complexes) on the membrane (see model-specific forms in the differential-equation models below), c_d is the concentration of (MinE-free) membrane-bound MinD dimers, and n_s is the Hill coefficient. Apart from this reaction, $\mathbf{d} \to \mathbf{D}$, all reactions in all models follow the law of mass action.

2.3 Asymmetric Activation Model (AAM)

We define the AAM such that

$$\begin{aligned}\frac{dc_d}{dt} &= (\omega_{D\to d}+\omega^d_{D\to d}c_d)(c_{\max}-c_d-c_{de})/c_{\max}-\omega_{E,d\to de}c_d-\omega_{d,e\to de}c_dc_e\\ &\quad -\omega_{d\to D}c_s^{n_s}c_d/(c_s^{n_s}+(c_{\bar{d}}+c_d+c_{de})^{n_s}), \end{aligned}\tag{S3a}$$

$$\frac{dc_{de}}{dt} = \omega_{E,d\to de}c_d+\omega_{d,e\to de}c_dc_e-\omega_{de\to D,E}c_{de}-\omega_{de\to D,e}c_{de}, \tag{S3b}$$

$$\frac{dc_e}{dt} = -\omega_{d,e\to de}c_dc_e+\omega_{de\to D,e}c_{de}-\omega_{e\to E}c_e. \tag{S3c}$$

Its MinD monomer density $= 2(c_d + c_{de}) + C_d$, and its MinE monomer density $= 2(c_{de} + c_e) + C_e$. For fitting to the MinD dissociation data with MinE in the flowed buffer, we set $\omega^x_{D\to d} = 0$ for $x \in \{\emptyset, d\}$. For fitting to the MinD dissociation data with MinE absent, we set $c_{de} = 0$, $c_e = 0$, and all nontrivial rate parameters except $\omega_{d\to D}$ equal to 0. We note that $\omega_{D\to d}$ and $\omega^d_{D\to d}$ have a multiplicative factor of c_D built into them and $\omega_{E,d\to de}$ has a multiplicative factor of c_E built into it.

2.4 Comprehensive Asymmetric Activation Model (CAAM)

The AAM assumes that membrane-bound MinD dimers, but not membrane-bound MinD-MinE tetramers, recruit bulk MinD dimers to bind to the membrane. In MinD and MinE bursts on a supported lipid bilayer *in vitro*, however, increasing the buffer concentration of MinE increases both the maximal net membrane-attachment rate of MinD and the peak membrane density of MinD [8]. Because a presumed increase of MinE binding to MinD on the membrane does not seem to suppress MinD's ability to recruit bulk MinD to bind to the membrane, we allow membrane-bound MinD-MinE tetramers to recruit bulk MinD dimers to bind to the membrane ($\mathbf{D} \xrightarrow{+de} \mathbf{d}$) in the CAAM. We know of no model that incorporates membrane-bound MinE facilitated recruitment of bulk MinE to bind to MinE-free MinD on the membrane. Without such, an increasing net membrane-attachment rate of MinE from roughly 5 s until 20 s in the MinD dissociation data would not likely be possible as the density of MinE-free MinD on the membrane, bulk MinE's substrate, presumably decreases during that time period. Thus, we allow membrane-bound MinD-MinE tetramers and membrane-bound MinE dimers to recruit bulk MinE dimers to bind to membrane-bound MinD dimers ($\mathbf{d}+\mathbf{E} \xrightarrow{+de} \mathbf{de}$ and $\mathbf{d}+\mathbf{E} \xrightarrow{+e} \mathbf{de}$) in the CAAM. Additionally, we allow the possibility of some backward reactions in the CAAM, as discussed in the Materials and Methods. Including the aforementioned reactions into the AAM, we define the CAAM such that

$$\begin{aligned}\frac{dc_d}{dt} &= (\omega_{D\to d} + \omega^d_{D\to d} c_d + \omega^{de}_{D\to d} c_{de})(c_{\max} - c_{\bar{d}} - c_d - c_{de})/c_{\max} \\ &\quad - (\omega_{E,d\to de} + \omega^{de}_{E,d\to de} c_{de} + \omega^e_{E,d\to de} c_e) c_d - \omega_{d,e\to de} c_d c_e + \omega_{de\to d,e} c_{de} \\ &\quad - \omega_{d\to D} c_s^{n_s} c_d / (c_s^{n_s} + (c_{\bar{d}} + c_d + c_{de})^{n_s}), \end{aligned} \tag{S4a}$$

$$\begin{aligned}\frac{dc_{de}}{dt} &= (\omega_{E,d\to de} + \omega^{de}_{E,d\to de} c_{de} + \omega^e_{E,d\to de} c_e) c_d + \omega_{d,e\to de} c_d c_e \\ &\quad - \omega_{de\to D,E} c_{de} - \omega_{de\to D,e} c_{de} - \omega_{de\to d,e} c_{de}, \end{aligned} \tag{S4b}$$

$$\frac{dc_e}{dt} = -\omega_{d,e\to de} c_d c_e + \omega_{de\to d,e} c_{de} + \omega_{de\to D,e} c_{de} - \omega_{e\to E} c_e. \tag{S4c}$$

Like the AAM, its MinD monomer density $= 2(c_d + c_{de}) + C_d$, and its MinE monomer density $= 2(c_{de} + c_e) + C_e$. For fitting to the MinD dissociation data with MinE in the flowed buffer, we set $\omega^x_{D\to d} = 0$ for $x \in \{\emptyset, d, de\}$. For fitting to the MinD dissociation data with MinE absent, like with the AAM, we set $c_{de} = 0$, $c_e = 0$, and all nontrivial rate parameters except $\omega_{d\to D}$ equal to 0. We note that $\omega^z_{D\to d}$ has a multiplicative factor of c_D built into it for $z \in \{\emptyset, d, de\}$ and $\omega^z_{E,d\to de}$ has a multiplicative factor of c_E built into it for $z \in \{\emptyset, de, e\}$.

2.5 Symmetric Activation Model (SAM)

We define the SAM such that

$$
\begin{aligned}
\frac{dc_d}{dt} &= (\omega_{D\to d} + \omega_{D\to d}^{d} c_d + \omega_{D\to d}^{de} c_{de} + \omega_{D\to d}^{ede} c_{ede})(c_{\max} - c_{\bar{d}} - c_d - c_{de} - c_{ede})/c_{\max} \\
&\quad - (\omega_{E,d\to de} + \omega_{E,d\to de}^{de} c_{de} + \omega_{E,d\to de}^{ede} c_{ede} + \omega_{E,d\to de}^{e} c_e) c_d \\
&\quad - \omega_{d,e\to de} c_d c_e + \omega_{de\to d,e} c_{de} - \omega_{d,ede\to de,de} c_d c_{ede} + \omega_{de,de\to d,ede} c_{de}^2 \\
&\quad - \omega_{d\to D} c_s^{n_s} c_d / (c_s^{n_s} + (c_{\bar{d}} + c_d + c_{de} + c_{ede})^{n_s}), && \text{(S5a)} \\
\frac{dc_{de}}{dt} &= (\omega_{E,d\to de} + \omega_{E,d\to de}^{de} c_{de} + \omega_{E,d\to de}^{ede} c_{ede} + \omega_{E,d\to de}^{e} c_e) c_d \\
&\quad - (\omega_{E,de\to ede} + \omega_{E,de\to ede}^{de} c_{de} + \omega_{E,de\to ede}^{ede} c_{ede} + \omega_{E,de\to ede}^{e} c_e) c_{de} \\
&\quad + \omega_{d,e\to de} c_d c_e - \omega_{de\to d,e} c_{de} + 2\omega_{d,ede\to de,de} c_d c_{ede} - 2\omega_{de,de\to d,ede} c_{de}^2 \\
&\quad - \omega_{de,e\to ede} c_{de} c_e + \omega_{ede\to de,e} c_{ede}, && \text{(S5b)} \\
\frac{dc_{ede}}{dt} &= (\omega_{E,de\to ede} + \omega_{E,de\to ede}^{de} c_{de} + \omega_{E,de\to ede}^{ede} c_{ede} + \omega_{E,de\to ede}^{e} c_e) c_{de} \\
&\quad - \omega_{d,ede\to de,de} c_d c_{ede} + \omega_{de,de\to d,ede} c_{de}^2 + \omega_{de,e\to ede} c_{de} c_e - \omega_{ede\to de,e} c_{ede} \\
&\quad - \omega_{ede\to D,e,e} c_{ede} - \omega_{ede\to E,D,e} c_{ede} - \omega_{ede\to E,D,E} c_{ede}, && \text{(S5c)} \\
\frac{dc_e}{dt} &= \omega_{de\to d,e} c_{de} - \omega_{d,e\to de} c_d c_e - \omega_{de,e\to ede} c_{de} c_e \\
&\quad + \omega_{ede\to de,e} c_{ede} + \omega_{ede\to E,D,e} c_{ede} + 2\omega_{ede\to D,e,e} c_{ede} - \omega_{e\to E} c_e. && \text{(S5d)}
\end{aligned}
$$

Its MinD monomer density $= 2(c_d + c_{de} + c_{ede}) + C_d$, and its MinE monomer density $= 2(c_{de} + 2c_{ede} + c_e) + C_e$. For fitting to the MinD dissociation data with MinE in the flowed buffer, we set $\omega_{D\to d}^{x} = 0$ for $x \in \{\emptyset, d, de, ede\}$. For fitting to the MinD dissociation data with MinE absent, we set $c_{de} = 0$, $c_{ede} = 0$, $c_e = 0$, and all nontrivial rate parameters except $\omega_{d\to D}$ equal to 0. We note that $\omega_{D\to d}^{z}$ has a multiplicative factor of c_D built into it for $z \in \{\emptyset, d, de, ede\}$ and $\omega_{E,d\to de}^{z}$ and $\omega_{E,de\to ede}^{z}$ have a multiplicative factor of c_E built into them for $z \in \{\emptyset, de, ede, e\}$.

2.6 Asymmetric Activation with Bridged Stability Model (AABSM)

We define the AABSM such that

$$
\begin{aligned}
\frac{dc_d}{dt} &= (\omega_{D\to d} + \omega_{D\to d}^{d} c_d + \omega_{D\to d}^{de} c_{de} + \omega_{D\to d}^{ded} c_{ded})(c_{\max} - c_{\bar{d}} - c_d - c_{de} - 2c_{ded})/c_{\max} \\
&\quad - (\omega_{E,d\to de} + \omega_{E,d\to de}^{de} c_{de} + \omega_{E,d\to de}^{ded} c_{ded} + \omega_{E,d\to de}^{e} c_e) c_d \\
&\quad - \omega_{d,de\to ded} c_d c_{de} + \omega_{ded\to d,de} c_{ded} - \omega_{d,e\to de} c_d c_e + \omega_{de\to d,e} c_{de} \\
&\quad - \omega_{d\to D} c_s^{n_s} c_d / (c_s^{n_s} + (c_{\bar{d}} + c_d + c_{de} + 2c_{ded})^{n_s}), && \text{(S6a)} \\
\frac{dc_{de}}{dt} &= (\omega_{E,d\to de} + \omega_{E,d\to de}^{de} c_{de} + \omega_{E,d\to de}^{ded} c_{ded} + \omega_{E,d\to de}^{e} c_e) c_d \\
&\quad + 2(\omega_{E,ded\to de,de} + \omega_{E,ded\to de,de}^{de} c_{de} + \omega_{E,ded\to de,de}^{ded} c_{ded} + \omega_{E,ded\to de,de}^{e} c_e) c_{ded} \\
&\quad - \omega_{d,de\to ded} c_d c_{de} + \omega_{ded\to d,de} c_{ded} + \omega_{d,e\to de} c_d c_e - \omega_{de\to d,e} c_{de} \\
&\quad - 2\omega_{de,de\to ded,e} c_{de}^2 + 2\omega_{ded,e\to de,de} c_{ded} c_e - \omega_{de\to D,E} c_{de} - \omega_{de\to D,e} c_{de}, && \text{(S6b)} \\
\frac{dc_{ded}}{dt} &= -(\omega_{E,ded\to de,de} + \omega_{E,ded\to de,de}^{de} c_{de} + \omega_{E,ded\to de,de}^{ded} c_{ded} + \omega_{E,ded\to de,de}^{e} c_e) c_{ded} \\
&\quad + \omega_{d,de\to ded} c_d c_{de} - \omega_{ded\to d,de} c_{ded} + \omega_{de,de\to ded,e} c_{de}^2 - \omega_{ded,e\to de,de} c_{ded} c_e, && \text{(S6c)} \\
\frac{dc_e}{dt} &= \omega_{de\to d,e} c_{de} - \omega_{d,e\to de} c_d c_e + \omega_{de,de\to ded,e} c_{de}^2 - \omega_{ded,e\to de,de} c_{ded} c_e \\
&\quad + \omega_{de\to D,e} c_{de} - \omega_{e\to E} c_e. && \text{(S6d)}
\end{aligned}
$$

Its MinD monomer density $= 2(c_d + c_{de} + 2c_{ded}) + C_d$, and its MinE monomer density $= 2(c_{de} + c_{ded} + c_e) + C_e$. For fitting to the MinD dissociation data with MinE in the flowed buffer, we set $\omega^x_{D\to d} = 0$ for $x \in \{\emptyset, d, de, ded\}$. For fitting to the MinD dissociation data with MinE absent, we set $c_{de} = 0$, $c_{ded} = 0$, $c_e = 0$, and all nontrivial rate parameters except $\omega_{d\to D}$ equal to 0. We note that $\omega^z_{D\to d}$ has a multiplicative factor of c_D built into it for $z \in \{\emptyset, d, de, ded\}$ and $\omega^z_{E,d\to de}$ and $\omega^z_{E,ded\to de,de}$ have a multiplicative factor of c_E built into them for $z \in \{\emptyset, de, ded, e\}$.

2.7 The FitzHugh Nagumo Model (FHNM)

This FHNM is simplified model of neuron firing, not the Min system, that we use to test how well an arbitrary model can describe the time-course data. The parameters in the model are a, b, c, d, I, v_l, and v_u. The FHNM model is defined such that

$$\frac{dv}{dt} = av(v - v_l)(v_u - v) - bw + I, \tag{S7a}$$

$$\frac{dw}{dt} = cv - dw. \tag{S7b}$$

We define its MinD monomer density as $v + w + C_d$ and its MinE monomer density as $w + C_e$. For fitting to the MinD dissociation data with MinE in the flowed buffer, we include all states and all parameters in the model. For fitting to the MinD dissociation data with MinE absent, we set $w = 0$.

3 Implementation of the Homotopy-Minimization Method for Data Fitting

We employ the Homotopy-Minimization Method for Parameter Estimation in Differential Equations [1] to fit our models to the time-course data. In this section, we first define our statistical model for use in the method, then we describe details of method implementation for the AAM, the CAAM, the SAM, and the AABSM. We omit details of the FHNM for brevity, but note that they are similar in nature to those discussed.

3.1 Statistical Model

For the jth (of n_y) data value d_{jk} measured at time t_k for $k \in \{1, 2 \ldots, n_t(j)\}$, corresponding observable model value y_{jk}, and error ε_{jk},

$$d_{jk} = y_{jk} + \varepsilon_{jk}. \tag{S8}$$

As examples, for just the oscillation data and the AAM, $n_y = 2$, d_{1k} is the measured density of MinD monomers at time t_k, d_{2k} is the density of MinE monomers at time t_k, $y_{1k} = 2(c_d + c_{de}) + C_d$ at time t_k, and $y_{2k} = 2(c_{de} + c_e) + C_e$ at time t_k. Errors, ε_{jk}, consist of modeling errors and data errors. We find that standard errors of the mean (SEMs) in the oscillation data range between roughly 5 μm^{-2} and 30 μm^{-2} while the data ranges on the scale of $5 \cdot 10^3$ μm^{-2}. We did not extract MinD dissociation data from raw data, so we do not know its SEMs, but we assume that they are also small compared to data ranges. As such, we expect modeling errors to be significantly larger than data errors, and thus expect errors, ε_{jk}, to consist primarily of modeling errors. Modeling errors are inherently not independent nor identically distributed, but without a better a priori distribution, we assume that ε_{jk} are independent and identically distributed from a normal distribution with a mean of 0, for $j = 1, \ldots, n_y$ and $k = 1, 2 \ldots, n_t(j)$. Ranges of protein densities vary in our time-course data. Thus, to remove bias in fitting from differences in scale, we assume that ε_{jk} are proportional to $\bar{y}_j$, the range of d_{jk} for $k = 1, 2 \ldots, n_t(j)$, for $j = 1, \ldots, n_y$ and $k = 1, 2 \ldots, n_t(j)$. Therefore, collectively, we assume that

$$d_{jk} = y_{jk} + \bar{y}_j \bar{\varepsilon}_{jk}, \tag{S9}$$

where $\bar{\varepsilon}_{jk}$ are independent and identically distributed from a normal distribution with a mean of 0 and a variance of $\bar{\sigma}^2$, $N(0, \bar{\sigma}^2)$, for $j = 1, \ldots, n_y$ and $k = 1, 2 \ldots, n_t(j)$. Thus, the likelihood of y_{jk} given d_{jk} and $\bar{\sigma}^2$ for $j = 1, \ldots, n_y$ and $k = 1, 2 \ldots, n_t(j)$ is given by

$$\mathcal{L}(y_{jk}|d_{jk}, \bar{\sigma}^2 : j = 1, \ldots, n_y \text{ and } k = 1, 2 \ldots, n_t(j)) =$$
$$\prod_{j=1}^{n_y} \prod_{k=1}^{n_t(j)} \frac{1}{\sqrt{2\pi \bar{\mathrm{y}}_j^2 \bar{\sigma}^2}} \exp\left(-\frac{(d_{jk} - y_{jk})^2}{\bar{\mathrm{y}}_j^2 \bar{\sigma}^2}\right) = \bar{C} \exp\left(\frac{1}{\bar{\sigma}^2} \sum_{j=1}^{n_y} \sum_{k=1}^{n_t(j)} -\frac{(d_{jk} - y_{jk})^2}{\bar{\mathrm{y}}_j^2}\right), \tag{S10}$$

for constant $\bar{C} > 0$. The values of y_{jk} for $j = 1, \ldots, n_y$ and $k = 1, 2 \ldots, n_t(j)$ that maximize the likelihood are those that minimize

$$\sum_{j=1}^{n_y} \sum_{k=1}^{n_t(j)} \frac{(d_{jk} - y_{jk})^2}{\bar{\mathrm{y}}_j^2}. \tag{S11}$$

Thus, we measure the difference in observable model values from time-course data by the sum of weighted squared residuals in Equation S11.

3.2 Defining Functionals, $r_{\mathrm{y}}(\mathbf{p}, \mathbf{x})$ and $r_{\Delta x}(\mathbf{p}, \mathbf{x})$, for the Homotopy-Minimization Method

As described above, we measure the difference in observable model values from time-course data by the sum of weighted squared residuals in Equation S11. Thus, we define $r_{\mathrm{y}}(\mathbf{p}, \mathbf{x})$, the measure of data fitting as defined in [1], such that

$$r_{\mathrm{y}}(\mathbf{p}, \mathbf{x}) = \frac{1}{\sum_{j=1}^{n_y} \sum_{k=1}^{n_t(j)} \bar{\mathrm{y}}_j^{-2} d_{jk}^2} \sum_{j=1}^{n_y} \sum_{k=1}^{n_t(j)} \bar{\mathrm{y}}_j^{-2} \left(d_{jk} - y_{jk}\right)^2, \tag{S12}$$

where we normalize by $\sum_{j=1}^{n_y} \sum_{k=1}^{n_t(j)} \bar{\mathrm{y}}_j^{-2} d_{jk}^2$ to match the scale of $r_{\mathrm{y}}(\mathbf{p}, \mathbf{x})$ in Equation 2.6 of [1]. We do not penalize deviations from interpolated data in our fitting, so we define $r_{\hat{\mathrm{y}}}(\mathbf{p}, \mathbf{x}) = 0$, for $r_{\hat{\mathrm{y}}}(\mathbf{p}, \mathbf{x})$ as described in Section 2.6 of [1].

For consistency with the notation in [1], we define $x_1 = c_d$, $x_2 = c_{de}$, and $x_3 = c_e$ ($n_x = 3$) for the AAM and the CAAM; $x_1 = c_d$, $x_2 = c_{de}$, $x_3 = c_{ede}$, and $x_4 = c_e$ ($n_x = 4$) for the SAM; and $x_1 = c_d$, $x_2 = c_{de}$, $x_3 = c_{ded}$, and $x_4 = c_e$ ($n_x = 4$) for the AABSM. We define $r_{\Delta x}(\mathbf{p}, \mathbf{x})$, the measure of satisfying a numerical solution to a differential-equation model as defined in [1], as in Equation 2.13a of [1] and discretize models using a Simpson's method finite difference, a finite difference with fourth order accuracy. Thus, in $r_{\Delta x}(\mathbf{p}, \mathbf{x})$,

$$\Delta x_{ik} = \begin{cases} 0 & \text{if } k \in \{1, n_t(i)\} \\ \dfrac{x_{i,k_+} - x_{i,k_-}}{2\Delta t} & \text{if } k \in \mathcal{I}_\Delta(i) \setminus \{1, n_t(i)\}, \end{cases}$$
$$F_{ik}(\mathbf{t}, \mathbf{p}, \mathbf{x}) = \begin{cases} 0 & \text{if } k \in \{1, n_t(i)\} \\ \displaystyle\sum_{m=-1}^{1} b_m \bar{F}_i\left(\mathbf{p}, x_{1,k+m}, x_{2,k+m}, \ldots, x_{n_x,k+m}\right) & \text{if } k \in \mathcal{I}_\Delta(i) \setminus \{1, n_t(i)\}, \end{cases} \tag{S13}$$

where k_+ is the index above k in $\mathcal{I}_\Delta(i)$ (the index set of the numerical discretization), k_- is the index below k in $\mathcal{I}_\Delta(i)$, Δt is the grid spacing in $\{t_k : k \in \mathcal{I}_\Delta(i)\}$, $b_{-1} = 1/6$, $b_0 = 4/6$, $b_1 = 1/6$, and $\bar{F}_i$ is the right-hand side of the ODE for x_i, for $i = 1, \ldots, n_x$. For smoothing penalties in $r_{\Delta x}(\mathbf{p}, \mathbf{x})$, $s_i(\mathbf{x})$ as defined in [1], we set $\alpha_i = 1$, $\beta_i = 10^2$, and $\gamma_i = 2$, for $i = 1, \ldots, n_x$, as implemented in [1] to insignificantly modify $r_{\Delta x}(\mathbf{p}, \mathbf{x})$ with a smooth set of state values and to strongly penalize $r_{\Delta x}(\mathbf{p}, \mathbf{x})$ with a jagged set of state values.

3.3 Domain Restrictions on States and Parameters

We restrict parameters and state values in models to be consistent with biological assumptions and certain experimental measurements. Rate parameters, $\omega^z_{u,v\to x,y}$ for $u, v, x, y, z \in \{\emptyset, D, E, d, de, ede, ded, e\}$, are only biologically relevant if nonnegative, and we assume that reactions are not overly fast, with rate parameters exceeding 10 units. Thus, we restrict rate parameters such that

$$0\ u_p \leq p \leq 10\ u_p \text{ for all relevant } p \in \left\{\omega^z_{u,v\to x,y} : u, v, x, y, z \in \{\emptyset, D, E, d, de, ede, ded, e\}\right\}, \quad \text{(S14)}$$

where u_p is the units of parameter p. Additionally, for the SAM and the AABSM including MinD-membrane-dissociation reactions of membrane-stable MinD states, as discussed in Table S1, to impose each model's core assumption, we restrict the rate of MinD dimer-membrane dissociation to be no faster in the membrane-stable MinD state than the membrane-unstable MinD state:

$$\omega_{de\to D,E} + \omega_{de\to D,e} \leq \omega_{ede\to D,e,e} + \omega_{ede\to E,D,e} + \omega_{ede\to E,D,E}, \quad \text{(S15a)}$$

for the SAM and

$$2\omega_{ded\to D,E,D} + \omega_{ded\to D,E,d} + \omega_{ded\to D,de} + 2\omega_{ded\to D,e,D} + \omega_{ded\to D,e,d} \leq \omega_{de\to D,E} + \omega_{de\to D,e}. \quad \text{(S15b)}$$

for the AABSM.

The parameter $c_{\max}$, which is only pertinent for fitting the oscillation data, dictates the maximum concentration of membrane-bound MinD dimers and is necessarily no less than half the range of the MinD monomer density in the time course, $\bar{D}/2$. Additionally, we assume that $c_{\max}$ is on the scale of $\bar{D}/2$, so we bound $c_{\max}$ above by $100 \cdot \bar{D}/2$. Thus, we restrict $c_{\max}$ such that

$$\bar{D}/2 \leq c_{\max} \leq 100 \cdot \bar{D}/2. \quad \text{(S16)}$$

We also assume that c_s, the half-max concentration constant in the Hill equation modeling the rate of spontaneous MinD-membrane dissociation, is on the scale of $\bar{D}/2$, so we bound c_s above by $100 \cdot \bar{D}/2$. The Hill equation modeling the rate of spontaneous MinD-membrane dissociation can be undefined if $c_s = 0$, so we bound c_s below by $1\ \mu m^{-2}$. Thus, we restrict c_s such that

$$1\ \mu m^{-2} \leq c_s \leq 100 \cdot \bar{D}/2. \quad \text{(S17)}$$

The Hill coefficient in the Hill equation modeling the rate of spontaneous MinD-membrane dissociation, n_s, is necessarily nonnegative, and we assume that it does not exceed a (biologically very large) value of 10, so we restrict n_s such that

$$0 \leq n_s \leq 10. \quad \text{(S18)}$$

The constant planar-density of bulk MinD monomers and the constant concentration of persistent membrane-bound MinD monomers, C_d, is necessarily nonnegative and no greater than the minimum MinD monomer density in each MinD time course, $D_{\min}$, so we restrict C_d such that

$$0\ \mu m^{-2} \leq C_d \leq D_{\min}. \quad \text{(S19)}$$

Similarly, for the oscillation data, C_e, the constant planar-density of bulk MinE monomers and the constant concentration of persistent membrane-bound MinE monomers, is necessarily nonnegative and no greater than the minimum MinE monomer density in the time course, $E_{\min}$. The density of MinE is $0\ \mu m^{-2}$ at the start of the MinD dissociation data with MinE in the flowed buffer, so $E_{\min}$ is not a meaningful upper bound of C_e for the time course. As such, we restrict C_e such that

$$0\ \mu m^{-2} \leq C_e \leq E_{\min} \quad \text{for the oscillation data,} \quad \text{(S20a)}$$

$$0\ \mu m^{-2} \leq C_e \quad \text{for the MinD dissociation data with MinE in the flowed buffer.} \quad \text{(S20b)}$$

The constant concentration of persistent membrane-bound MinD dimers, $c_{\bar{d}}$, is necessarily nonnegative and no greater than half the value of C_d, which includes the concentration of persistent membrane-bound MinD monomers in it, so we restrict $c_{\bar{d}}$ such that

$$0\ \mu m^{-2} \leq c_{\bar{d}} \leq C_d/2. \tag{S21}$$

Concentrations c_d, c_{de}, c_{ede}, c_{ded}, and c_e are only biologically relevant if nonnegative. Thus, we restrict c_d, c_{de}, c_{ede}, c_{ded}, and c_e to nonnegative values:

$$c_{i,k} \geq 0 \text{ for all } i \in \{d, de, ede, ded, e\} \text{ and } k \in \mathcal{I}_\Delta(i), \tag{S22}$$

where $c_{d,k}$, $c_{de,k}$, $c_{ede,k}$, $c_{ded,k}$, and $c_{e,k}$ are the values of c_d, c_{de}, c_{ede}, c_{ded}, and c_e at the kth index of the numerical discretization.

All aforementioned restrictions on parameters and state vales can be written as a collection of linear inequalities. Thus, during accelerated descent, as implemented in overlapping-niche descent of the Homotopy-Minimization Method, we employ projection using Dykstra's method as discussed in Section C.2.3 of [1]. In doing so, we choose a small relative termination tolerance, $\varepsilon_c = 10^{-6}$, and a smaller absolute termination tolerance, $\varepsilon_{\bar{c}} = 10^{-12}$. To avoid overly slowing accelerated descent from a large number of projections, we prematurely terminate accelerated descent if the number of iterations in Dykstra's method exceeds 10^4.

3.4 Generating Random Parameter and State Values

Following the procedure of overlapping-niche descent in the Homotopy-Minimization Method, as discussed in Section C.1 of [1], we randomly generate parameters and state values initially and in random offspring. Given no prior parameter-value estimates, we randomly generate rate parameters over a broad range of scales in accordance with their bounds, Equation S14:

$$p \sim 10^{U(-9,1)}\ u_p \text{ for all relevant } p \in \left\{\omega^z_{u,v\to x,y} : u, v, x, y, z \in \{\emptyset, D, E, d, de, ede, ded, e\}\right\}, \tag{S23}$$

where $U(a, b)$ is the uniform probability distribution over the interval (a, b) and u_p is the units of parameter p.

We expect $c_{\max}$, which is only pertinent for fitting the oscillation data, to be within one or two orders of magnitude of half the range of the MinD monomer density in the time course, $\bar{D}/2$. Thus, in accordance with the bounds on $c_{\max}$, Equation S16, we randomly generate $c_{\max}$ such that

$$c_{\max} \sim \bar{D}/2 \cdot 10^{U(0,2)}. \tag{S24}$$

Similarly, we expect c_s to be within one or two orders of magnitude of $\bar{D}/2$. Thus, in accordance with the bounds on c_s, Equation S17, we randomly generate c_s such that

$$c_s \sim \bar{D}/2 \cdot 10^{U(-2,2)}. \tag{S25}$$

We randomly generate n_s, C_d, C_e, and $c_{\bar{d}}$ uniformly over their respective bounds, Equations S18, S19, S20, and S21:

$$n_s \sim U(0, 10), \tag{S26}$$
$$C_d \sim U(0, D_{\min})\ \mu\text{m}^{-2}, \tag{S27}$$
$$C_e \sim U(0, E_{\min})\ \mu\text{m}^{-2}, \tag{S28}$$
$$c_{\bar{d}} \sim U(0, D_{\min}/2)\ \mu\text{m}^{-2}. \tag{S29}$$

We choose random state values to match time-course data exactly. Thus, for the oscillation data and the MinD dissociation data with MinE in the flowed buffer, MinD monomer density D, MinE

monomer density E, $D_2 = (D - C_d)/2$, and $E_2 = (E - C_e)/2$, we generate random state values for the AAM and the CAAM with c_e as a free state such that

$$\begin{aligned} c_e &\sim U(\max\{0, E_2 - D_2\}, E_2), \\ c_{de} &= E_2 - c_e, \\ c_d &= D_2 - E_2 + c_e. \end{aligned} \tag{S30}$$

Similarly, we generate random state values for the SAM with c_{ede} and c_e as free states such that

$$\begin{aligned} c_{ede}, c_e &\sim U\left(\{c_{ede} + c_e \geq E_2 - D_2, 2c_{ede} + c_e \leq E_2 : c_{ede} \geq 0, c_e \geq 0\}\right), \\ c_{de} &= E_2 - 2c_{ede} - c_e, \\ c_d &= D_2 - E_2 + c_{ede} + c_e, \end{aligned} \tag{S31}$$

and we generate random state values for the AABSM with c_{ded} and c_e as free states such that

$$\begin{aligned} c_{ded}, c_e &\sim U\left(\{c_e - c_{ede} \geq E_2 - D_2, c_{ede} + c_e \leq E_2 : c_{ded} \geq 0, c_e \geq 0\}\right), \\ c_{de} &= E_2 - c_{ded} - c_e, \\ c_d &= D_2 - E_2 - c_{ded} + c_e, \end{aligned} \tag{S32}$$

where $U(\{\cdot\})$ is the uniform probability distribution over the set $\{\cdot\}$. Additionally, for the MinD dissociation data with MinE absent, we generate random state values for the AAM, the CAAM, the SAM, and the AABSM with no free states such that

$$c_d = D_2. \tag{S33}$$

3.5 Random Perturbation and Selection

During the generation of sexual offspring in overlapping-niche descent of the Homotopy-Minimization Method, as described in Section C.1 of [1], for each sexual offspring, we define the standard deviation of perturbation, $\tilde{\sigma}$, such that

$$\tilde{\sigma} = \begin{cases} F_c & \text{with probability } 0.5 \\ U(0,1) & \text{with probability } 0.5, \end{cases} \tag{S34a}$$

where F_c is a measure of convergence defined below and $U(a, b)$ is the uniform probability distribution over the interval (a, b). Then, for parameter p in a sexual offspring inherited from individual $(\mathbf{p}_{g,i,j}, \mathbf{x}_{g,i,j})$, we perturb the value of the inherited parameter, $\hat{p}$, such that

$$F_c = \max\{\Delta r_{g,i,j}, 10^{-2}\}, \tag{S35a}$$

$$p \sim \begin{cases} \left(\hat{p} + \hat{p} \cdot N\left(0, \tilde{\sigma}^2\right) \;\middle|\; p_{\min} \leq p \leq p_{\max}\right) & \text{if } p \in \{n_s, C_d, C_e, c_{\bar{d}}\} \\ \left(\hat{p} \cdot 10^{N\left(0,\tilde{\sigma}^2\right)} \;\middle|\; p_{\min} \leq p \leq p_{\max}\right) & \text{otherwise,} \end{cases} \tag{S35b}$$

where $\Delta r_{g,i,j}$ is the measure of convergence in the jth parent space of the ith niche in generation g as defined in Equation C.1 of [1], $N(\mu, \sigma^2)$ is the normal distribution with mean μ and variance σ^2, and $p_{\min}$ and $p_{\max}$ are the restricted lower and upper bounds of parameter p as discussed in Section 3.3. In the standard deviation of perturbation, $\tilde{\sigma}$, $\max\{\Delta r_{g,i,j}, 10^{-2}\}$ ensures diminishing but significant perturbations in $\hat{p}$ as the algorithm converges, and $U(0, 1)$ allows for a wide range of perturbations in $\hat{p}$. Similarly, for state value x in a sexual offspring inherited from individual $(\mathbf{p}_{g,i,j}, \mathbf{x}_{g,i,j})$, we perturb the value of the inherited state value, $\hat{x}$, such that

$$F_c = \max\{\Delta r_{g,i,j}, 10^{-2}\}, \tag{S36a}$$

$$x \sim \left(\hat{x} + \hat{x} \cdot N\left(0, \tilde{\sigma}^2\right) \;\middle|\; x \geq 0\right). \tag{S36b}$$

During selection in overlapping-niche descent, as described in Section C.1 of [1], we choose the natural default value for the selection strength, $q_{\text{fit}} = 1$.

3.6 Remaining Details for Implementation of the Homotopy-Minimization Method

We choose values of λ to define niches in overlapping-niche descent of the Homotopy-Minimization Method as in Section 3.4.3 of [1]. We choose parents and offspring in overlapping-niche descent as in Section E.1.2 of [1]. For initial gradient scaling values, $s_{i,0}$ for i in the indexed set of all parameters and state values, as described in Section C.2.1 of [1], we choose $s_{i,0} = 10^{-12}$ for all i corresponding to parameters to ensure that projections onto restrictions involving multiple parameters are initially defined, and we choose $s_{i,0} = 0$ for all i corresponding to state values. We choose $n_{\max}$, ε_σ, ε_r, $n_{\max}$, $\breve{\sigma}$, m_{pro}, $\hat{n}_{\text{pro}}$, $\breve{n}_{\text{pro}}$, and $\varepsilon_{\Delta r}$ in overlapping-niche descent as in Section E.1.5 of [1]. Finally, we calculate confidence intervals for parameters using bootstrapping, as in Section 3.4.4 of [1].

We computed genetic-algorithm components of the Homotopy-Minimization Method using *MATLAB*, and we computed accelerated-descent components of the Homotopy-Minimization Method in parallel using *C++* on the Calcul Québec server Guillimin, the Compute Canada servers Cedar and Graham, and the WestGrid server Orcinus.

3.7 Numerical-Discretization Refinements

Initially, we fit each model to the time-course data using the data grid as the numerical-discretization grid. For some of the models, the numerical solution that fit the time-course data best would contain spurious oscillations. In such cases, we refined the numerical-discretization grid, increasing ρ, the ratio of the number of (evenly spaced) grid points in the numerical discretization to the number of data points in the time course, by whole numbers until the spurious oscillations disappeared. As such, for the AAM, $\rho = 3$ for the oscillation data, and $\rho = 2$ for the MinD dissociation data; for the CAAM, $\rho = 1$ for the oscillation data, and $\rho = 3$ for the MinD dissociation data; for the SAM, $\rho = 1$ for both sets of the time-course data; for the AABSM, $\rho = 1$ for both sets of the time-course data; and for the FHNM, $\rho = 3$ for both sets of the time-course data.

4 Data and Model Fits Not Shown in the Main Text

See Figs. S2, S3, S4, and S5.

5 State Values from Model Fits Not Shown in the Main Text

See Figs. S6, S7, S8, and S9.

6 Fittings of the SAM and the AABSM to the Time Course Data Under Alterations, Perturbations, and Constraints

See Tables S1 and S2, and Figs. S10.

7 Spatiotemporal Simulations of the Models

To simulate spatiotemporal patterning using the AAM, CAAM, SAM, and AABSM, we define reaction-diffusion forms the models, the RD-AAM, RD-CAAM, RD-SAM, and RD-AABSM: $\partial c_z/\partial t = R_z + D_z \Delta c_z$ for states $z \in \{d, de, ded, ede, e\}$ in the respective models, where R_z is the right-hand side of dc_z/dt in Eq. S3, S4, S5, and S6, D_z is the diffusion coefficient of c_z, and Δ is the 2-D Laplacian in x and y. In simulation of the the RD-AAM, RD-CAAM, RD-SAM, and RD-AABSM, we use reaction parameters from the fits of the AAM, CAAM, SAM, and AABSM to the oscillation data, as given

in Tables S3, S4, S5, and S6, and choose diffusion coefficients to be distributed throughout roughly the range of measured MinD and MinE diffusion coefficients in a traveling wave *in vitro*, $0.143 - 0.374\ \mu m^2\ s^{-1}$ [5], based on the relative sizes of the complexes in the states, $D_{ded}, D_{ede}, D_{de}, D_d, D_e = 0.1, 0.15, 0.2, 0.25, 0.35\ \mu m^2\ s^{-1}$.

In Ivanov and Mizuuchi's experiments, relatively small, roughly circular zones of higher MinD concentration than on surrounding areas of the membrane appear seemingly randomly in space, spread outwardly, and fuse. This progression initiates the onset of a period of a spatially near-homogeneous oscillation of MinD and MinE on the membrane (see Fig. 1A of [4]). As the number of initiation zones decreases in time, the formation of spatially near-homogeneous oscillations transitions into the formation of traveling waves of MinD and MinE on the membrane (see Movie S1 of [4]). The RD-AAM, RD-CAAM, RD-SAM, and RD-AABSM do not model stochastic events in space. As such, in our simulations of these models, we impose structure that mimics the appearance of initiation zones. To do so, we simply increase the value of c_d in our simulation by a fixed amount in N_i circular zones at certain times. We find a mean initiation-zone radius of 2.40 μm, with a standard deviation of 0.58 μm, and a mean change in mean MinD monomer density from the previous data frame of 596 μm^{-2}, with a standard deviation of 192 μm^{-2}, for 11 initiation zones appearing at the start of oscillations in Ivanov and Mizuuchi's experiments. So, when we simulate the appearance of an initiation zone, we increase the value of c_d by 596/2 μm^{-2} in a circle of radius 2.40 μm. If multiple initiation zones overlap when doing so, we increase the value of c_d by only 596/2 μm^{-2} in their intersection. For initial conditions in our simulations, we use the final values from our fits of the of AAM, CAAM, SAM, and AABSM to the oscillation data, $(c_d, c_{de}, c_e) = (5.88 \cdot 10^{-4}, 5.00, 3.39)\ \mu m^{-2}$, $(c_d, c_{de}, c_e) = (10.4, 50.7, 79.9)\ \mu m^{-2}$, $(c_d, c_{de}, c_{ede}, c_e) = (2.74, 1.05, 4.90, 1.92)\ \mu m^{-2}$, and $(c_d, c_{de}, c_{ded}, c_e) = (5.07, 116, 1.16, 8.14)\ \mu m^{-2}$, to simulate pattern formation at all oscillation onsets after the first oscillation, which starts with null concentrations. To simulate patterning in an open field, we employ zero-Dirichlet boundary conditions. We calculate our simulation results by numerically solving the RD-AAM, RD-CAAM, RD-SAM, and RD-AABSM using the the method of lines, discretizing the Laplacian using central-second-order finite differences and numerically solving the system in time using the MATLAB ODE solver ode15s.

We simulate the RD-AAM, RD-CAAM, RD-SAM, and RD-AABSM with $N_i = 1000, 100, 10,$ and 1 initiation zones randomly distributed in space with equal probability at time $t = 0\ s$, as described above. We do so on a square domain of $85\frac{1}{3}\ \mu m \times 85\frac{1}{3}\ \mu m$, with the height of that in Ivanov and Mizuuchi's microscopy images, using a numerical grid with uniform spacing of $85\frac{1}{3} \cdot 10^{-2}\ \mu m$. As shown in Fig. S11 and S14, we find that the RD-AAM and RD-AABSM exhibit the formation of spatially near-homogeneous oscillations with a larger number of initiation zones, oscillations with decreasing spatial homogeneity as the number of initiation zones decreases, and traveling waves with a smaller number of initiation zones. To show that this transition can occur continuously with periodicity similar to that observed experimentally, we simulate the RD-AAM and RD-AABSM with $N_i = 1000$ at $t = 0\ s$, $N_i = 100$ at $t = 500\ s$, $N_i = 10$ at $t = 1000\ s$, and $N_i = 1$ at $t = 1500\ s$, with initiation zones distributed as in the simulations with the same N_i but occurring only at $t = 0\ s$. The solutions follow essentially the same trajectory as a concatenation of the solutions with $N_i = 1000, 100, 10$ at $t = 0\ s$ for the RD-AAM and $N_i = 1000, 100, 10, 1$ at $t = 0\ s$ for the RD-AABSM (again, see Fig. S11 and S14). This shows that under initiation conditions similar to those seen in Ivanov and Mizuuchi's experiments, the RD-AAM and RD-AABSM can recapitulate the experimentally-observed transition from spatially near-homogeneous oscillations to traveling waves. In contrast, as shown in Fig. S12, the RD-CAAM only exhibits the formation of spatially near-homogeneous oscillations. This occurs because the fit of the CAAM to the oscillation data is a solution that oscillates later in time than the fitted data, so in simulations of the RD-CAAM with a small number of initiation zones, oscillations originate outside of spreading initiation zones and disrupt the formation of a traveling wave. Further from experimental observations, as shown in Fig. S13, the RD-SAM only shows MinD depletion in initiation zones followed by low-amplitude spatially-near-homogeneous oscillations later in time. This occurs because initiation zones as implemented fail to stimulate excitation in the RD-AAM and the fit of the AAM to the oscillation data is a solution with low-amplitude oscillations later in time than the fitted data.

Spiral waves, as observed in Ivanov and Mizuuchi's experiments, presumably arise from a break in symmetry in a traveling wave. The emergence of a traveling wave occurs in our simulations with a small number of initiation zones with some overlap for the RD-AAM and a single initiation zone for the RD-AABSM. As such, to simulate spiral waves, we numerically solve the RD-AAM and RD-AABSM starting with a single initiation zone at $(x, y) = (0, 0)$ μm at time $t = 0$ s, as described above, except that the radius of the initiation zone for the RD-AAM is twice as large as specified. Then, when a traveling wave forms, we break the symmetry in it by imposing the condition that $(c_d, c_{de}, c_e) = (5.88 \cdot 10^{-4}, 5.00, 3.39)$ μm^{-2} for the RD-AAM and $(c_d, c_{de}, c_{ded}, c_e) = (5.07, 116, 1.16, 8.14)$ μm^{-2} for the RD-AABSM, the values of initial conditions outside of initiation zones, for $y \geq x$. For these simulations, we numerically solve the RD-AAM and RD-AABSM on a square domain of $170\frac{2}{3}$ $\mu m \times 170\frac{2}{3}$ μm, with a height twice that of Ivanov and Mizuuchi's microscopy images for a somewhat more expansive view of the spiral wave's behavior, using a numerical grid with uniform spacing of $170\frac{2}{3} \cdot 10^{-2}$ μm, and impose the symmetry breaking condition at $t = 500$ s. As can be seen in Fig. S15 and S16, spiral waves emerge and persists in our simulations. Before breaking the symmetries in the traveling waves, the wave speeds were roughly 0.17 μm s^{-1} for the RD-AAM and 0.34 μm s^{-1} for the RD-AABSM. At the end of the simulations, the outward propagating speeds of the spiral waves were roughly 0.30 μm s^{-1} for the RD-AAM and 0.29 μm s^{-1} for the RD-AABSM. In comparison, Ivanov and Mizuuchi measured wave speeds of $0.4 - 0.7$ μm s^{-1} in their experiments [4].

8 Parameter Estimates from Fits

We provide parameter estimates from our fits of the models to the time-course data below. In them, if a parameter is blank, it was not included in the fitting. All parameters with non-blank and non-zero values were included in the parameter counts of AIC scores. Because the time-course data is not very noisy, fitting errors consist primarily of modeling errors, and modeling errors rather than independent and identically distributed Gaussian errors were redistributed during the calculation of confidence intervals by bootstrapping. As such, the confidence intervals shown are not very biologically informative and are often smaller for models that fit the time-course data better, but we provide them for reference anyway.

References

[1] William Christopher Carlquist. *A homotopy-minimization method for parameter estimation in differential equations and its application in unraveling the reaction mechanism of the Min system.* PhD thesis, University of British Columbia, 2019.

[2] Tamara Heermann, Beatrice Ramm, Samson Glaser, and Petra Schwille. Local self-enhancement of MinD membrane binding in Min protein pattern formation. *Journal of Molecular Biology*, 432(10):3191–3204, 2020.

[3] Tamara Heermann, Frederik Steiert, Beatrice Ramm, Nikolas Hundt, and Petra Schwille. Mass-sensitive particle tracking to elucidate the membrane-associated MinDE reaction cycle. *Nature methods*, 18(10):1239–1246, 2021.

[4] Vassili Ivanov and Kiyoshi Mizuuchi. Multiple modes of interconverting dynamic pattern formation by bacterial cell division proteins. *Proceedings of the National Academy of Sciences*, 107(18):8071–8078, 2010.

[5] Martin Loose, Elisabeth Fischer-Friedrich, Christoph Herold, Karsten Kruse, and Petra Schwille. Min protein patterns emerge from rapid rebinding and membrane interaction of MinE. *Nature structural & molecular biology*, 18(5):577–583, 2011.

[6] Martin Loose, Elisabeth Fischer-Friedrich, Jonas Ries, Karsten Kruse, and Petra Schwille. Spatial regulators for bacterial cell division self-organize into surface waves in vitro. *Science*, 320(5877):789–792, 2008.

[7] Atsushi Miyagi, Beatrice Ramm, Petra Schwille, and Simon Scheuring. High-speed atomic force microscopy reveals the inner workings of the MinDE protein oscillator. *Nano Letters*, 18(1):288–296, 2018.

[8] Anthony G Vecchiarelli, Min Li, Michiyo Mizuuchi, Ling Chin Hwang, Yeonee Seol, Keir C Neuman, and Kiyoshi Mizuuchi. Membrane-bound MinDE complex acts as a toggle switch that drives Min oscillation coupled to cytoplasmic depletion of MinD. *Proceedings of the National Academy of Sciences*, 113(11):E1479–E1488, 2016.
