# Supplementary figures and images for: The mechanism of MinD stability modulation by MinE in Min protein dynamics
Source: PLoS Comput Biol. 2023 Nov 17;19(11):e1011615. doi: 10.1371/journal.pcbi.1011615 (PMC10691731; doi:10.1371/journal.pcbi.1011615)

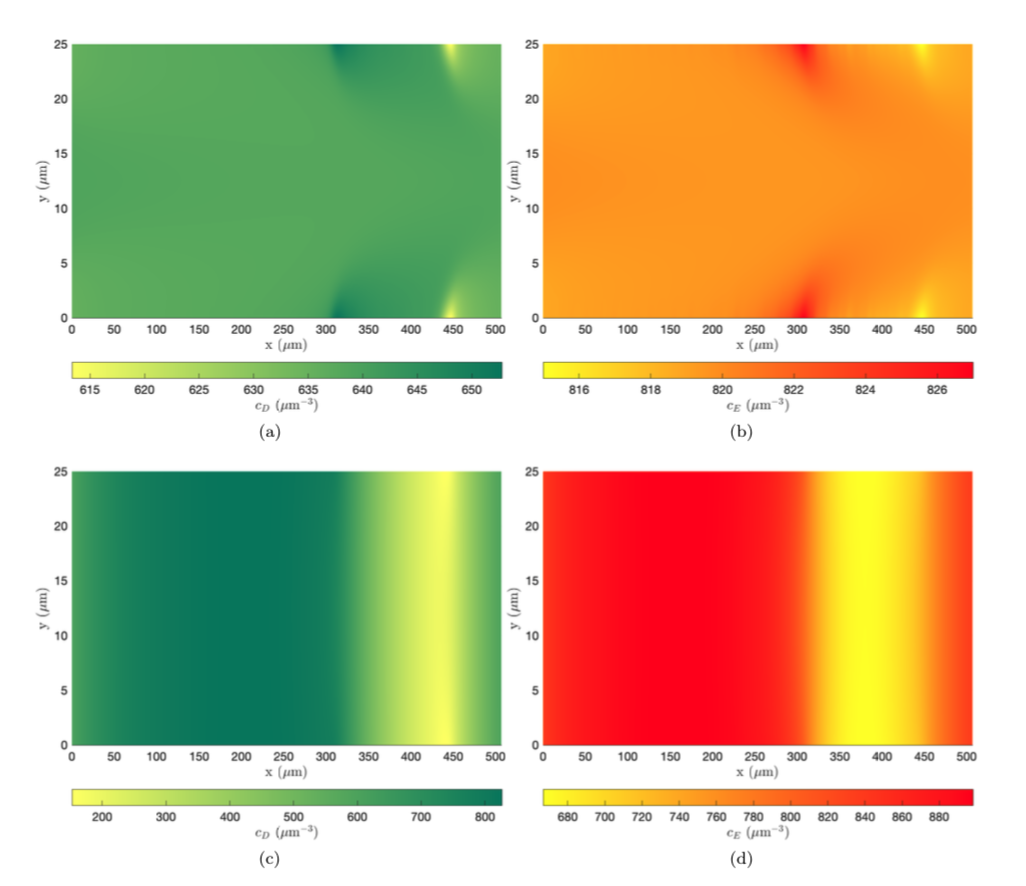

Supplement: S1 Fig — The simulated concentrations of MinD and MinE (cD and cE) inside a flow cell with in-phase oscillation of MinD and MinE on both the top and bottom of the flow cell, with a temporal offset in space in the oscillations as in the data from which the oscillation data was generated. The numerical solutions to Eq. S1 are shown in (a) and (b) long after having converged to a propagating wave solution that is periodic on the domain shown and moves to the right, at 507 simulated seconds, the time passed during the oscillation data. The vertical scale (y) is expanded relative to the horizontal scale by a factor of ∼20 for the sake of legibility. Yellow shows where the Min proteins are slightly depleted from the bulk at the leading edge of the wave and darker green/red shows slight enrichment in its wake. cD and cE do not vary much from cD¯=638.3μm-3 and cE¯=819.0μm-3 because the rapid rate of flow inside the flow cell acts to homogenize the concentrations of MinD and MinE. This is in sharp contrast to cD and cE shown in (c) and (d) from an identical simulation except without flow, v¯=0μms-1. Note the dramatic difference in the color scales between (a)/(b) and (c)/(d). (PNG) [file pcbi.1011615.s002.png]

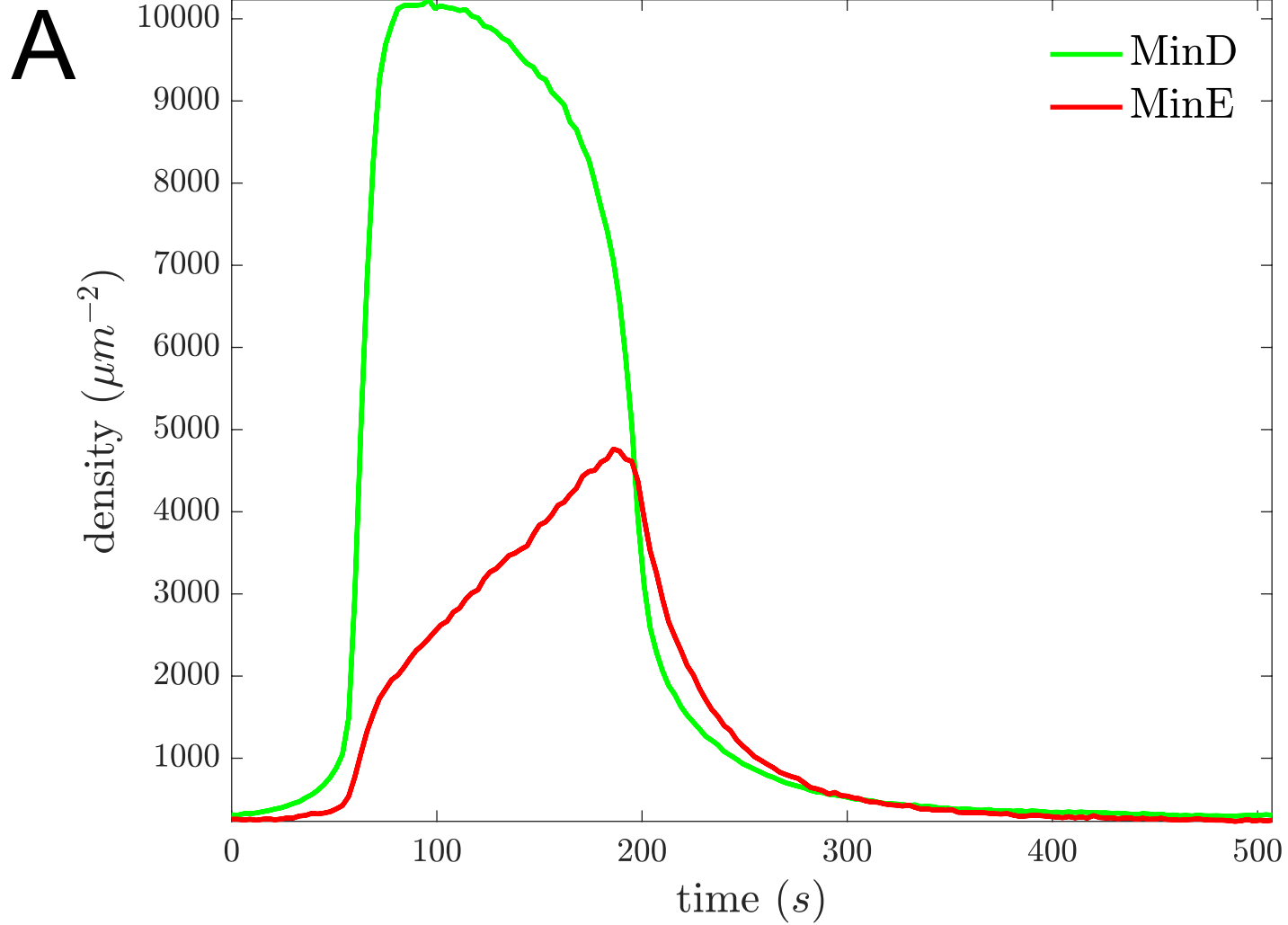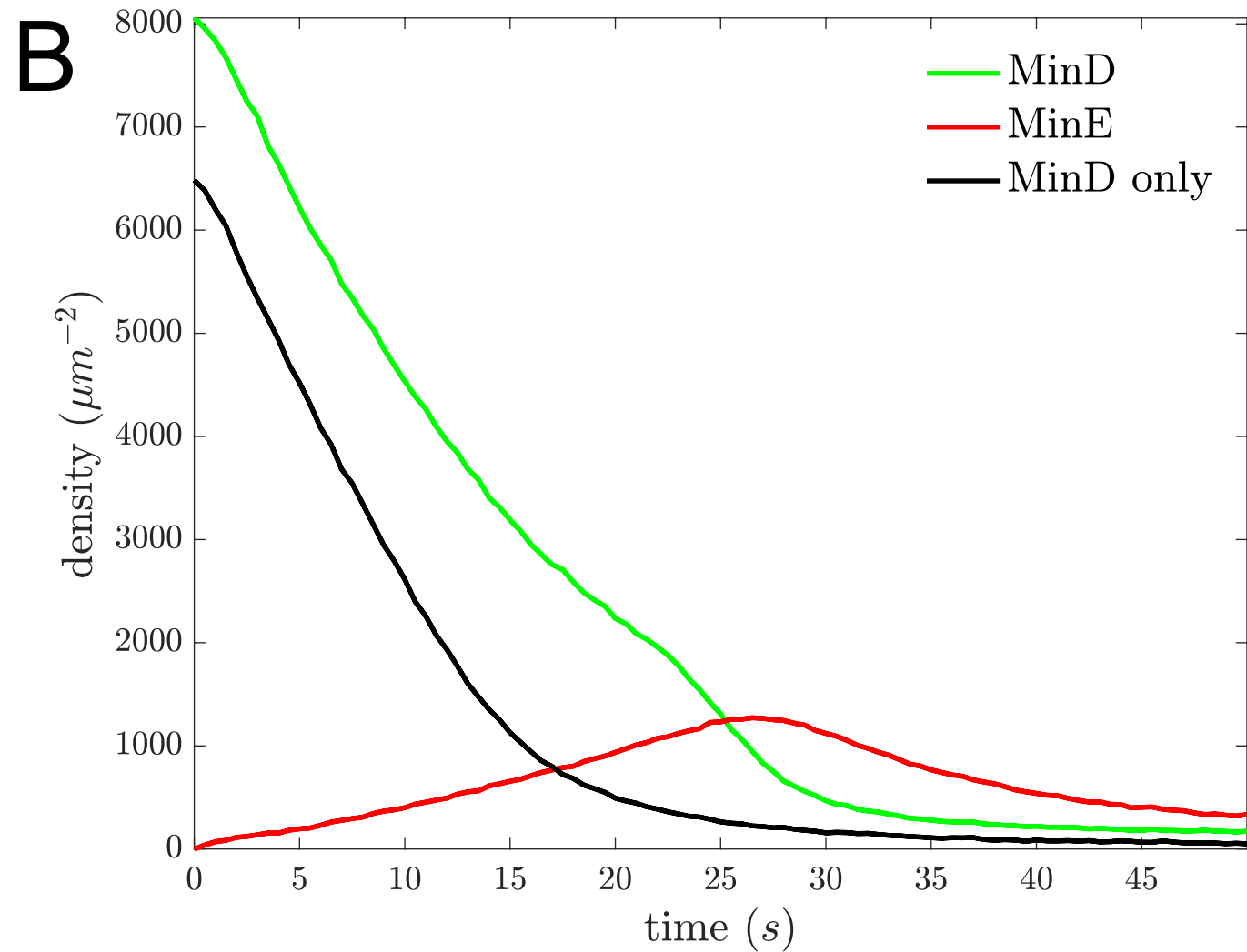

Supplement: S2 Fig — The oscillation data is shown in Panel A, and MinD dissociation data is shown in Panel B. Densities shown are the total concentrations of MinD and MinE, monomers μm−2. SEMs in the data are omitted because they would be indistinguishable from the means shown by eye. (PDF) [file pcbi.1011615.s003.pdf]

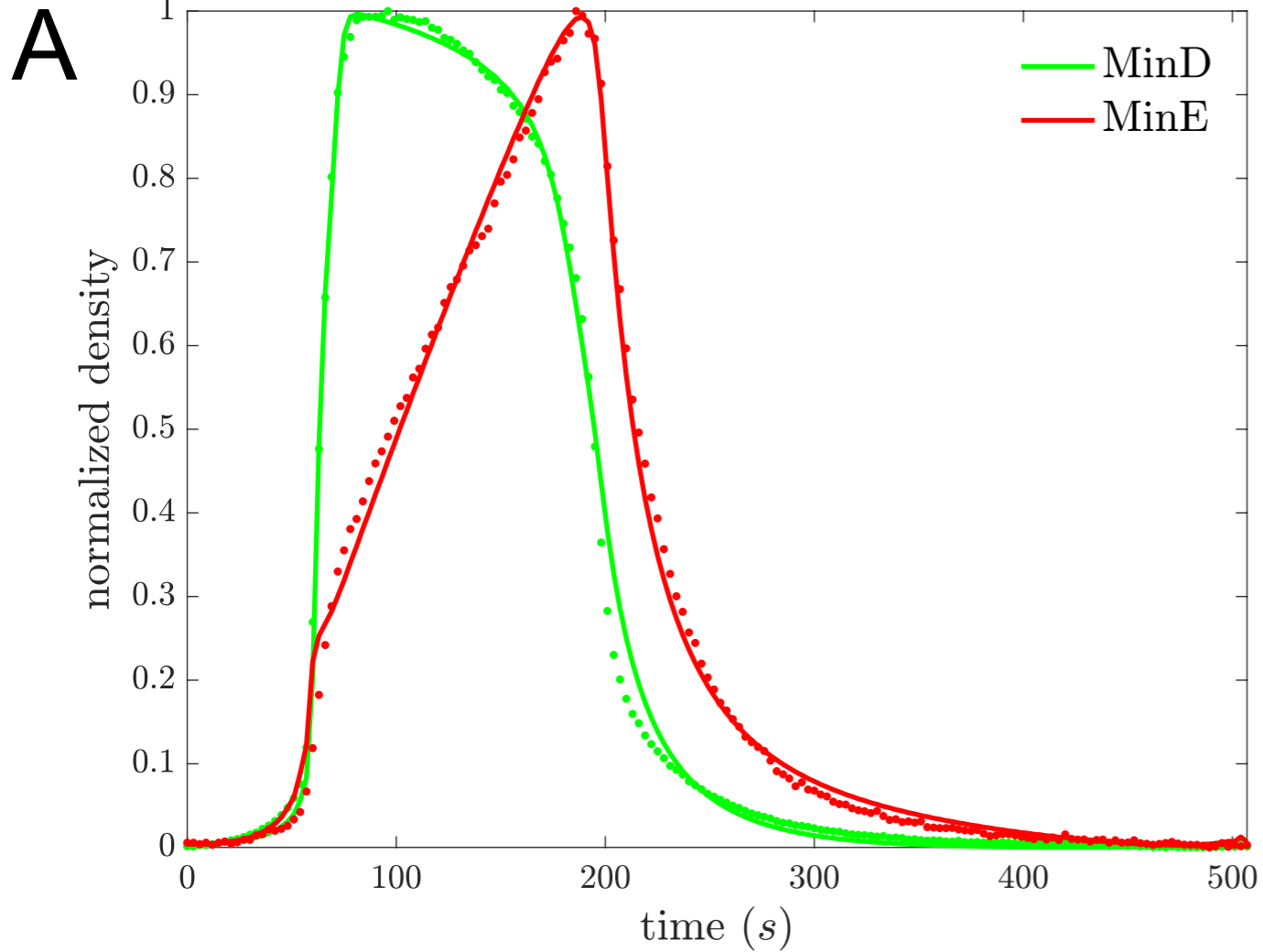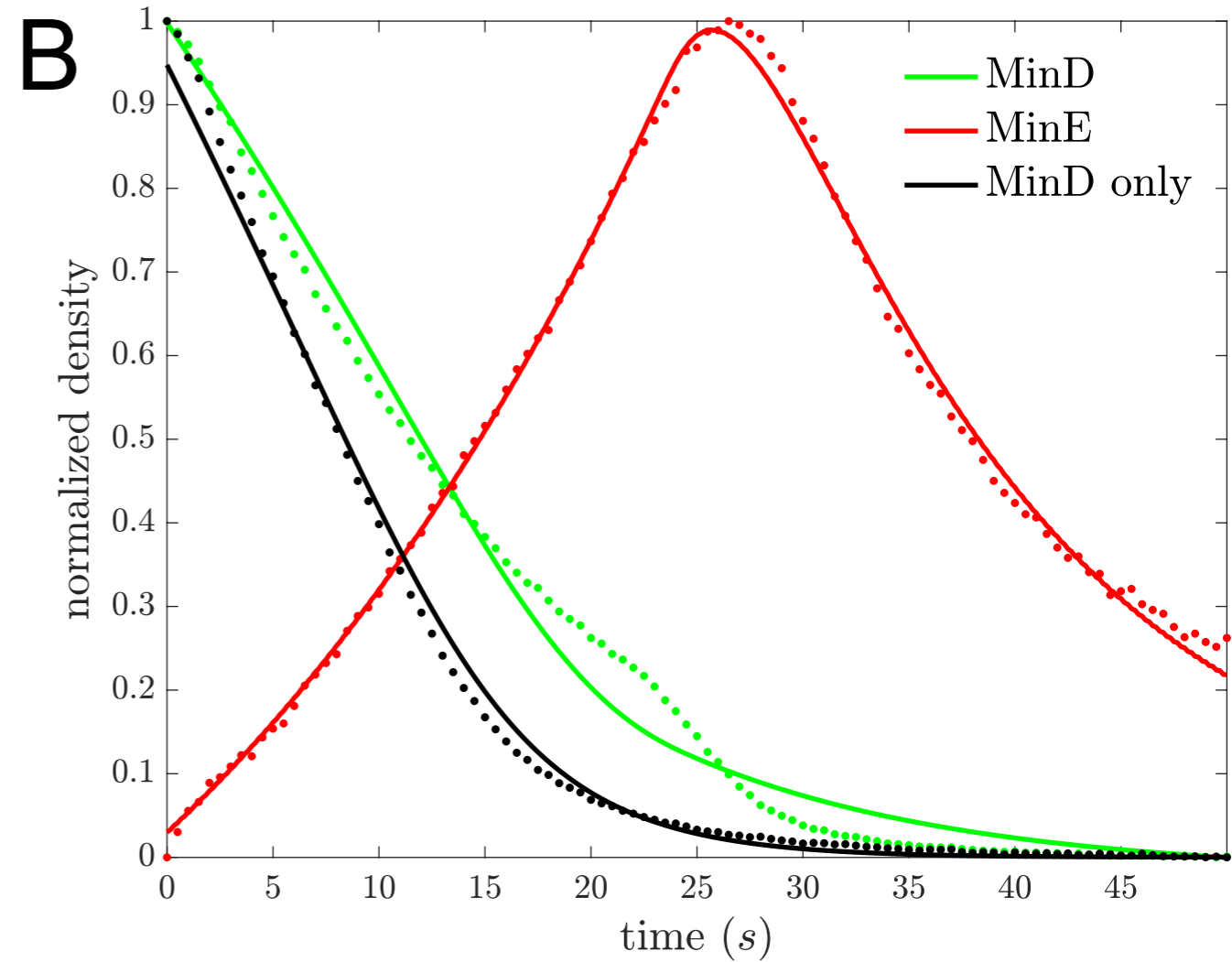

Supplement: S3 Fig — For the CAAM, the fit to the oscillation data is shown in Panel A, and the fit to the MinD dissociation data is shown in Panel B. Fits are solid, and the data are dotted. (PDF) [file pcbi.1011615.s004.pdf]

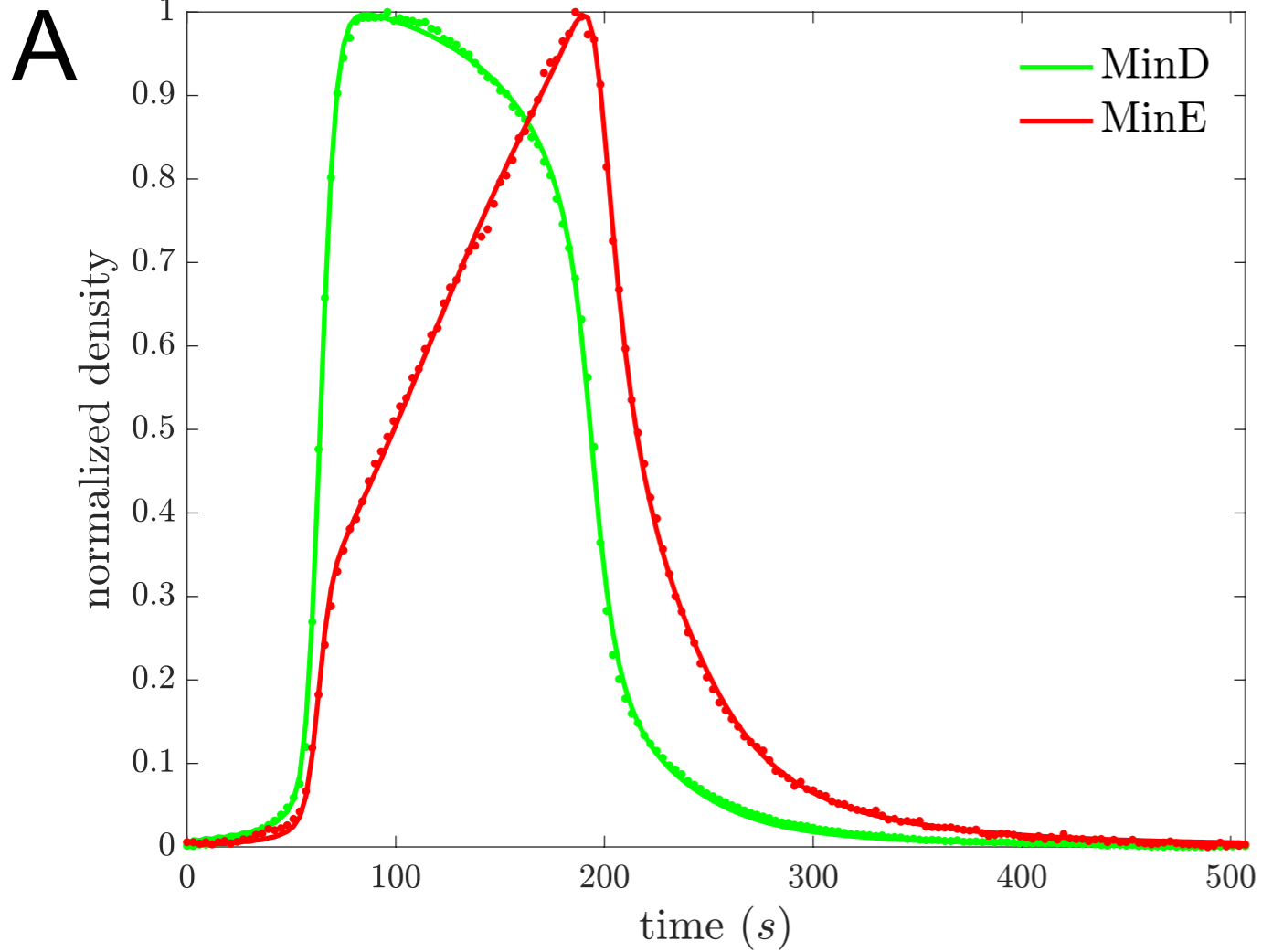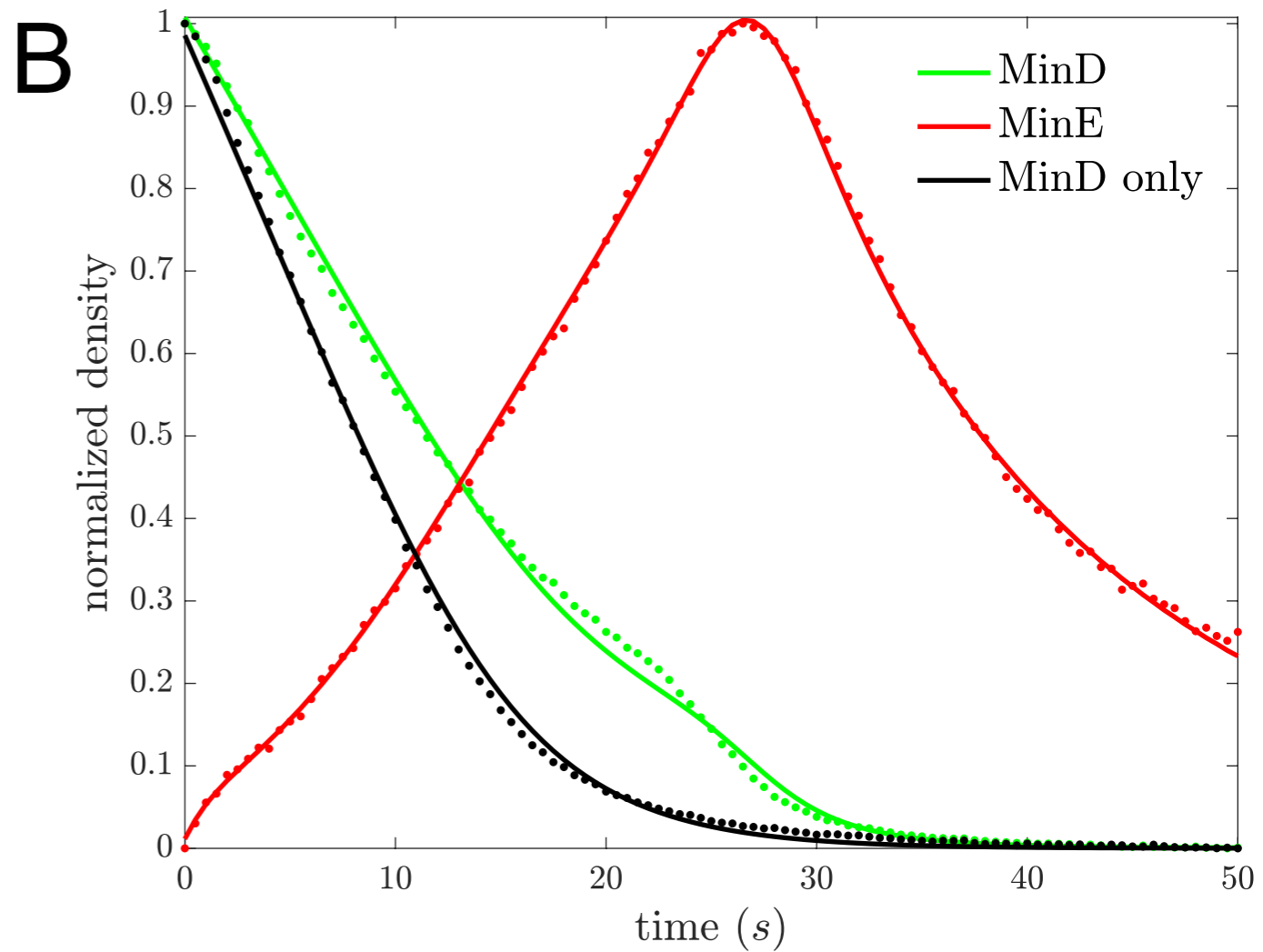

Supplement: S4 Fig — For the SAM, the fit to the oscillation data is shown in Panel A, and the fit to the MinD dissociation data is shown in Panel B. Fits are solid, and the data are dotted. (PDF) [file pcbi.1011615.s005.pdf]

**A**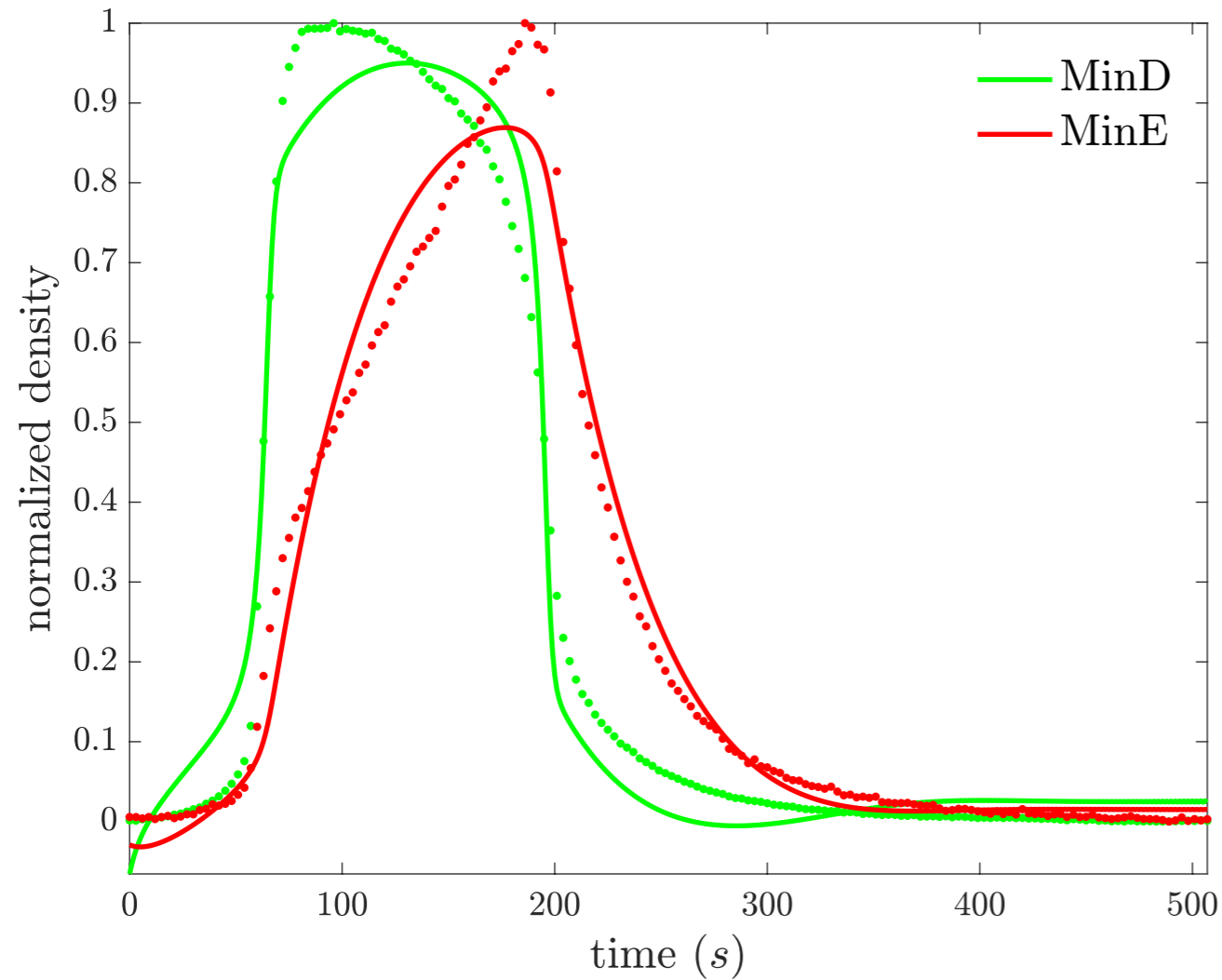**B**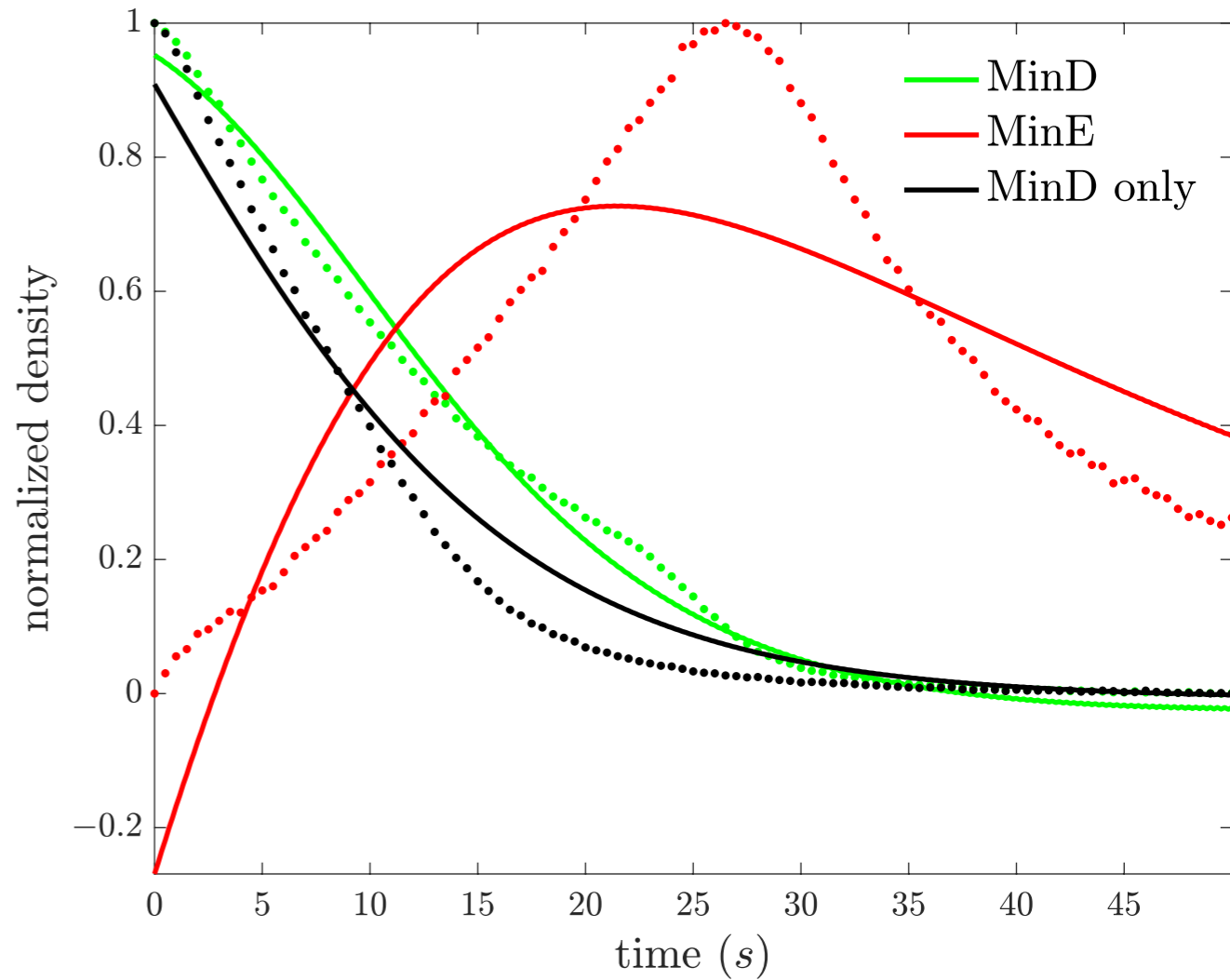

Supplement: S5 Fig — For the FHNM, the fit to the oscillation data is shown in Panel A, and the fit to the MinD dissociation data is shown in Panel B. Fits are solid, and the data are dotted. (PDF) [file pcbi.1011615.s006.pdf]

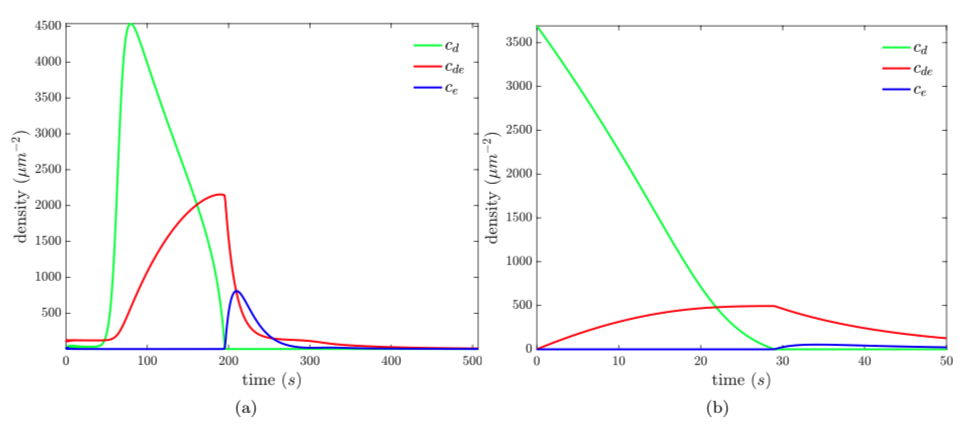

Supplement: S6 Fig — For the AAM, state values from the fit to the oscillation data are shown in (a), and state values from the fit to the MinD dissociation data with MinE in the flowed buffer are shown in (b). (PNG) [file pcbi.1011615.s007.png]

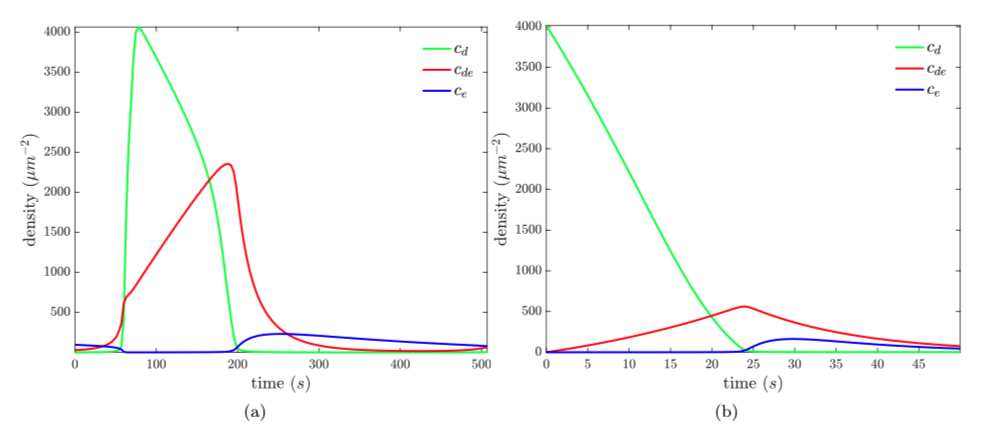

Supplement: S7 Fig — For the CAAM, state values from the fit to the oscillation data are shown in (a), and state values from the fit to the MinD dissociation data with MinE in the flowed buffer are shown in (b). (PNG) [file pcbi.1011615.s008.png]

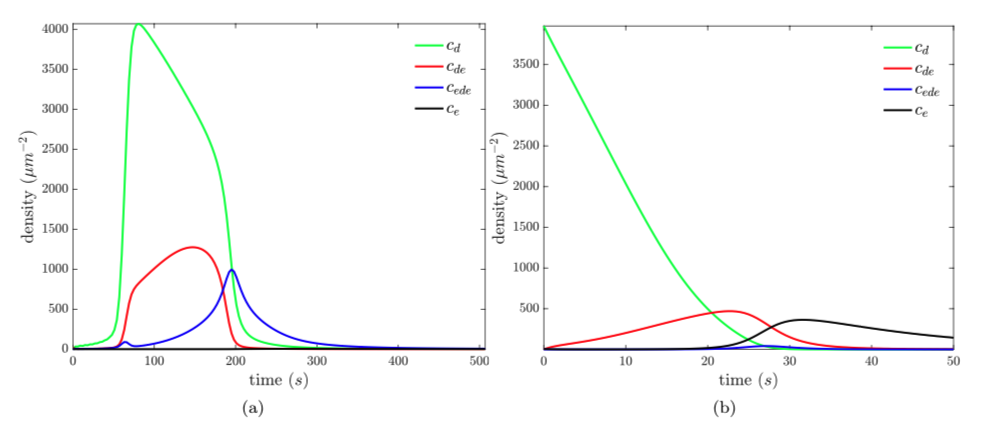

Supplement: S8 Fig — For the SAM, state values from the fit to the oscillation data are shown in (a), and state values from the fit to the MinD dissociation data with MinE in the flowed buffer are shown in (b). (PNG) [file pcbi.1011615.s009.png]

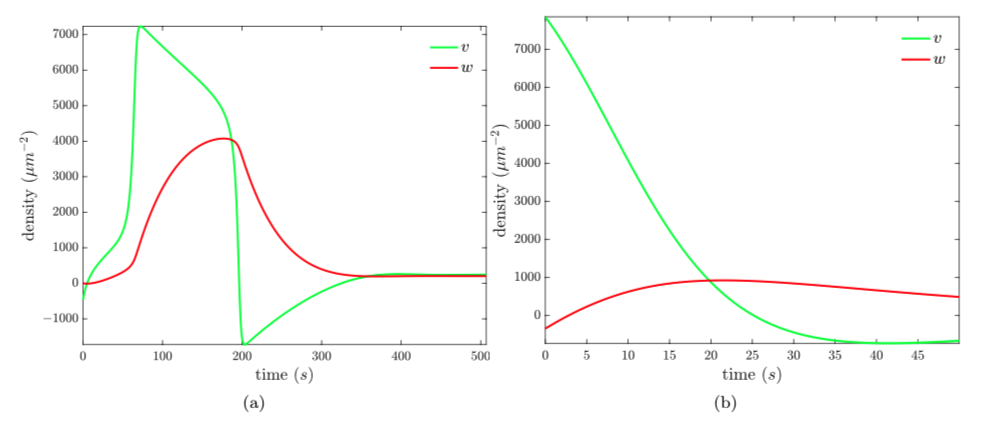

Supplement: S9 Fig — For the FHNM, state values from the fit to the oscillation data are shown in (a), and state values from the fit to the MinD dissociation data with MinE in the flowed buffer are shown in (b). (PNG) [file pcbi.1011615.s010.png]

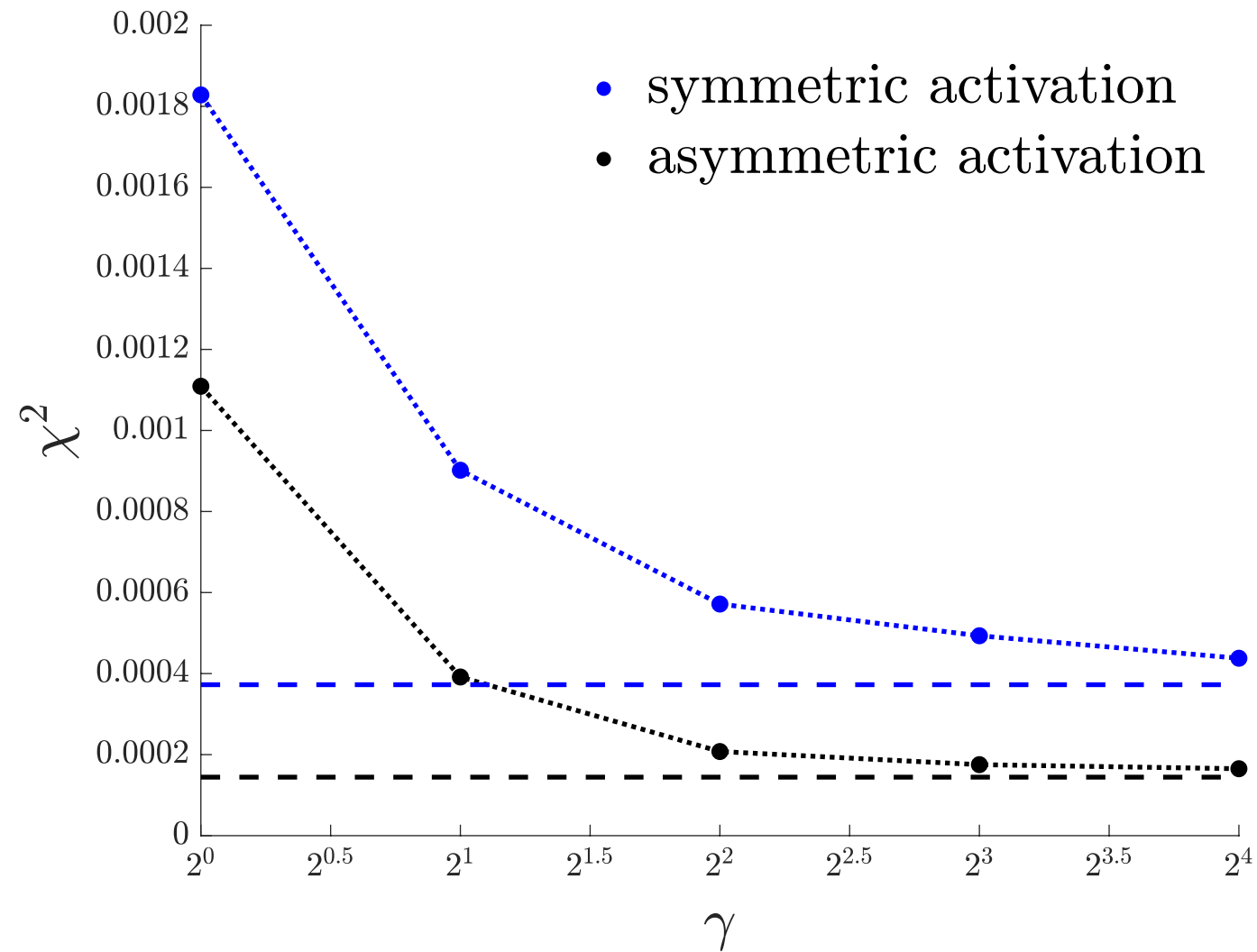

Supplement: S10 Fig — For each parameter p that is nontrivial in both the model of the oscillation data and the model of the MinD dissociation data with MinE in the flowed buffer, except for constant-concentration parameters Cd, Ce, and cd¯, we constrain p in the fit of the oscillation data to be less than or equal to γp in the fit of the MinD dissociation data, and we constrain p in the fit of the MinD dissociation data to be less than or equal to γp in the fit of the oscillation data, for γ = 1, 2, 4, 8, 16. The resulting χ2 values from the fits, the weighted sums of squared residuals, are shown as dots. The χ2 values from unconstrained optimizations, in which γ = ∞, are shown as flat dashed lines. When parameters across the data sets are constrained to be within roughly an order of magnitude of each other, the models, most notably the AABSM, can still recapitulate both data sets almost as well as without constraints. In fitting, we take into account differences in buffer MinE concentrations—1.36μM for the oscillation data and 2.5μM for the MinD dissociation data with MinE in the flowed buffer—by multiplying rate parameters of bulk MinE binding reactions, ωE,d→dez and ωE,de→edez for z ∈ {∅, de, ede, e} in the SAM and ωE,d→dez and ωE,ded→de,dez for z ∈ {∅, de, ded, e} in the AABSM, which otherwise have a multiplicative factor of cE built into them, by 1.36μM or 2.5μM. This removes the multiplicative factor of cE from the rate parameters of bulk MinE binding reactions and instead incorporates a conversion factor from bulk MinE monomers to dimers in them, under the assumption that all bulk MinE is stable in the dimer state. Parameter estimates for the AABSM with γ = 16 are shown in S8 Table. (PDF) [file pcbi.1011615.s011.pdf]

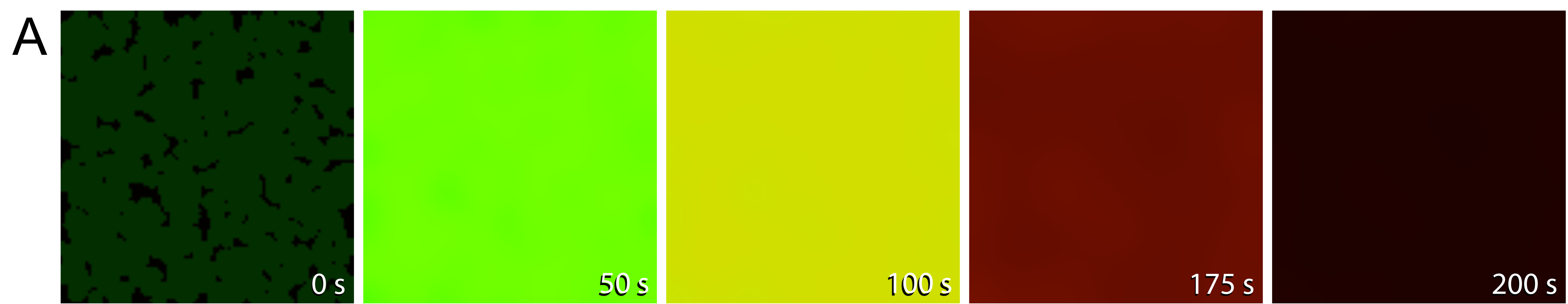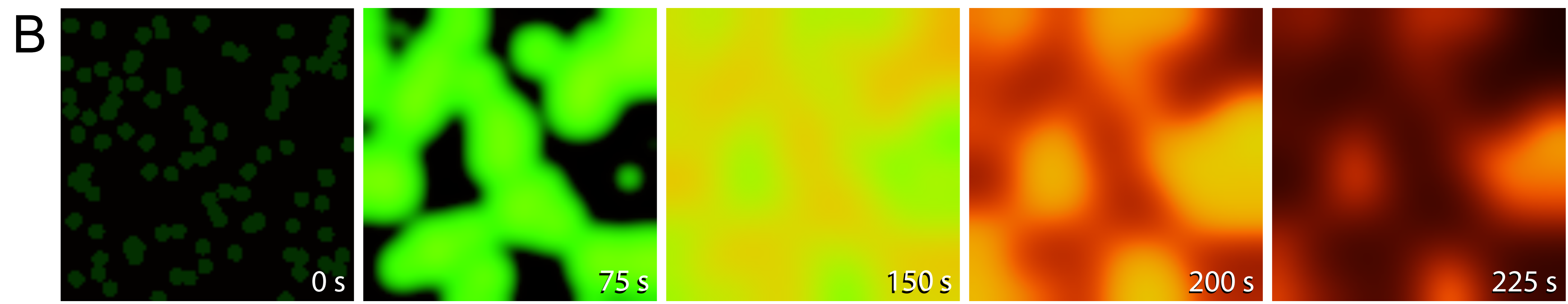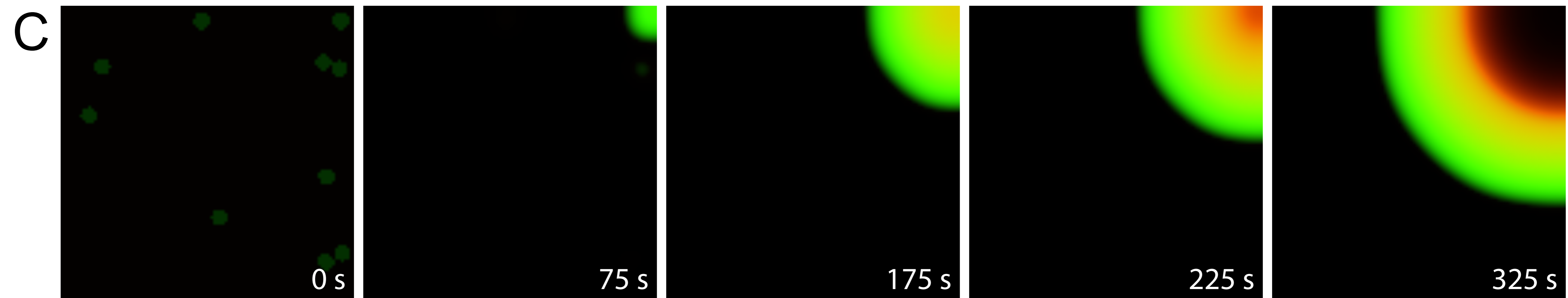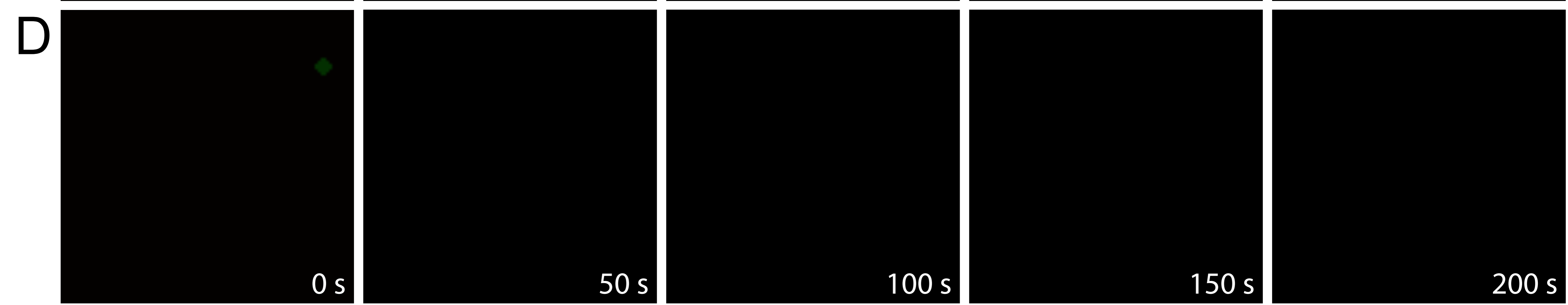

Supplement: S11 Fig — Solutions to the RD-AAM with Ni = 1000, 100, 10, and 1 initiation zones at time t = 0 s are shown in Panels A, B, C, and D at the displayed times. The solution to the RD-AAM with Ni = 1000 at t = 0 s, Ni = 100 at t = 500 s, Ni = 10 at t = 1000 s, and Ni = 1 at t = 1500 s is essentially the same as the concatenation of Panels A-D through time but differs from that shown in Panel D in that the traveling wave from the final frame of Panel C continues to propagate (overriding the initiation zone in the first frame of Panel D). MinD and MinE are shown in green and red on the same scale in all frames except for the first frame of each panel. The first frame of each panel is shown with green and red intensity scales that are amplified by a factor of three so that initiation zones are visible. Each square shown is 8513μm×8513μm, with a height of the microscopy images in Ivanov and Mizuuchi’s experiments. (PDF) [file pcbi.1011615.s012.pdf]

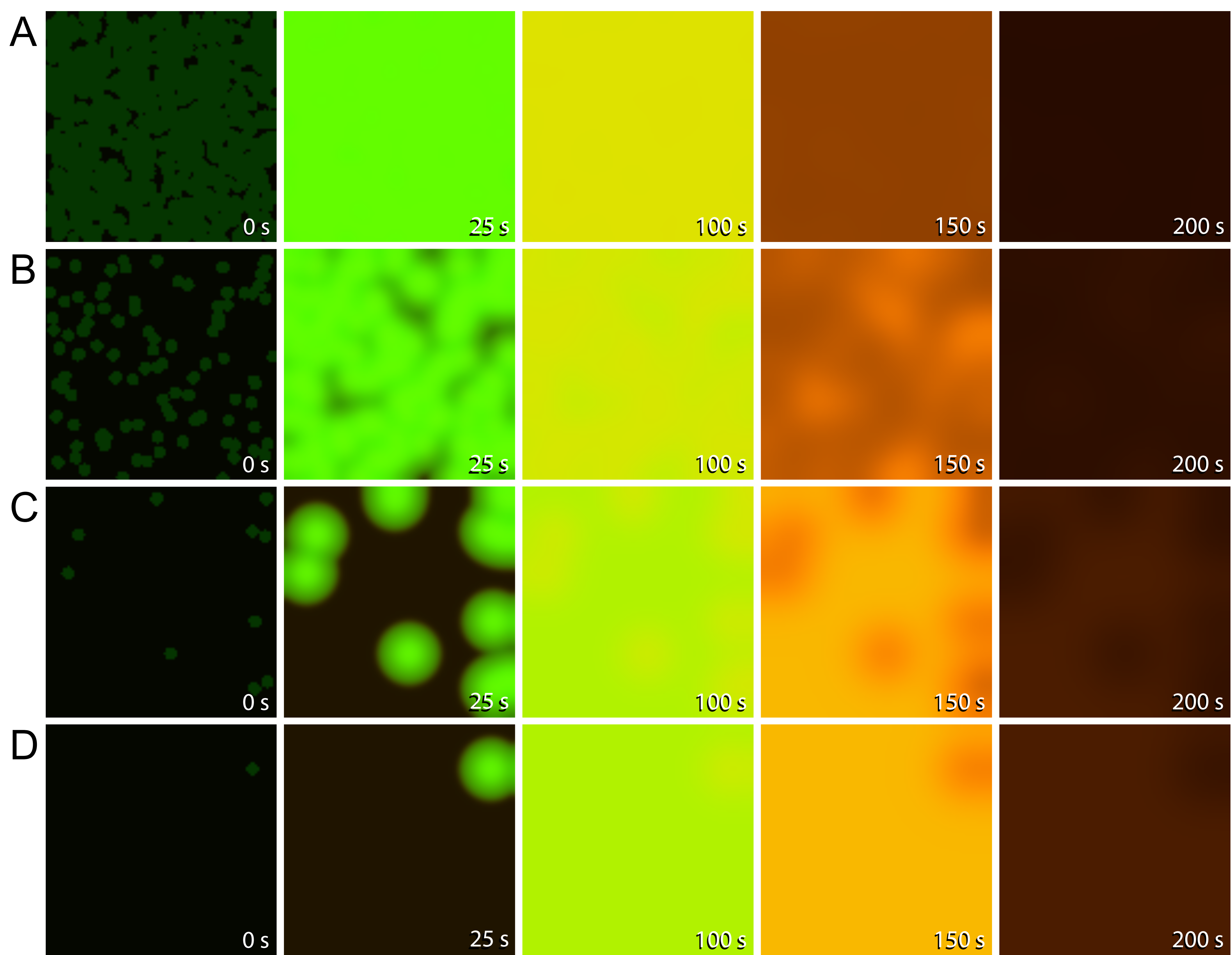

Supplement: S12 Fig — Solutions to the RD-CAAM with Ni = 1000, 100, 10, and 1 initiation zones at time t = 0 s are shown in Panels A, B, C, and D at the displayed times. Between the second and third frames of Panels C and D, innate oscillations cause excitation outside of expanding initiation zones, suppressing the formation of outward growing traveling waves. Color and spatial scales are as described in S11 Fig. (PDF) [file pcbi.1011615.s013.pdf]

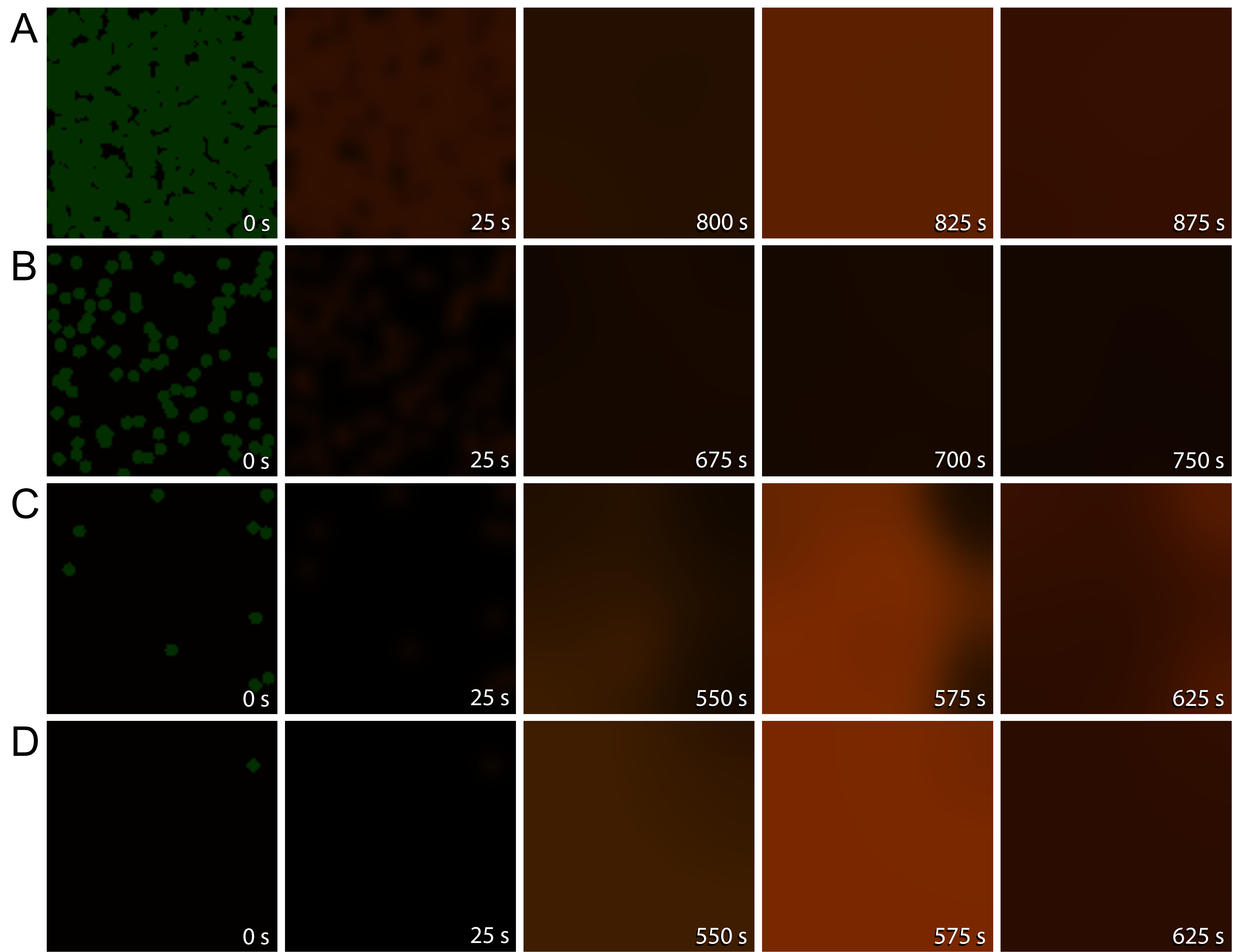

Supplement: S13 Fig — Solutions to the RD-SAM with Ni = 1000, 100, 10, and 1 initiation zones at time t = 0 s are shown in Panels A, B, C, and D at the displayed times. Initiation zones as implemented do not drive spatially near-homogeneous oscillations nor traveling-wave formation. Although it is hardly perceptible in Panel B, a low-amplitude oscillation occurs, with a peak around 700 s. Color and spatial scales are as described in S11 Fig. (PDF) [file pcbi.1011615.s014.pdf]

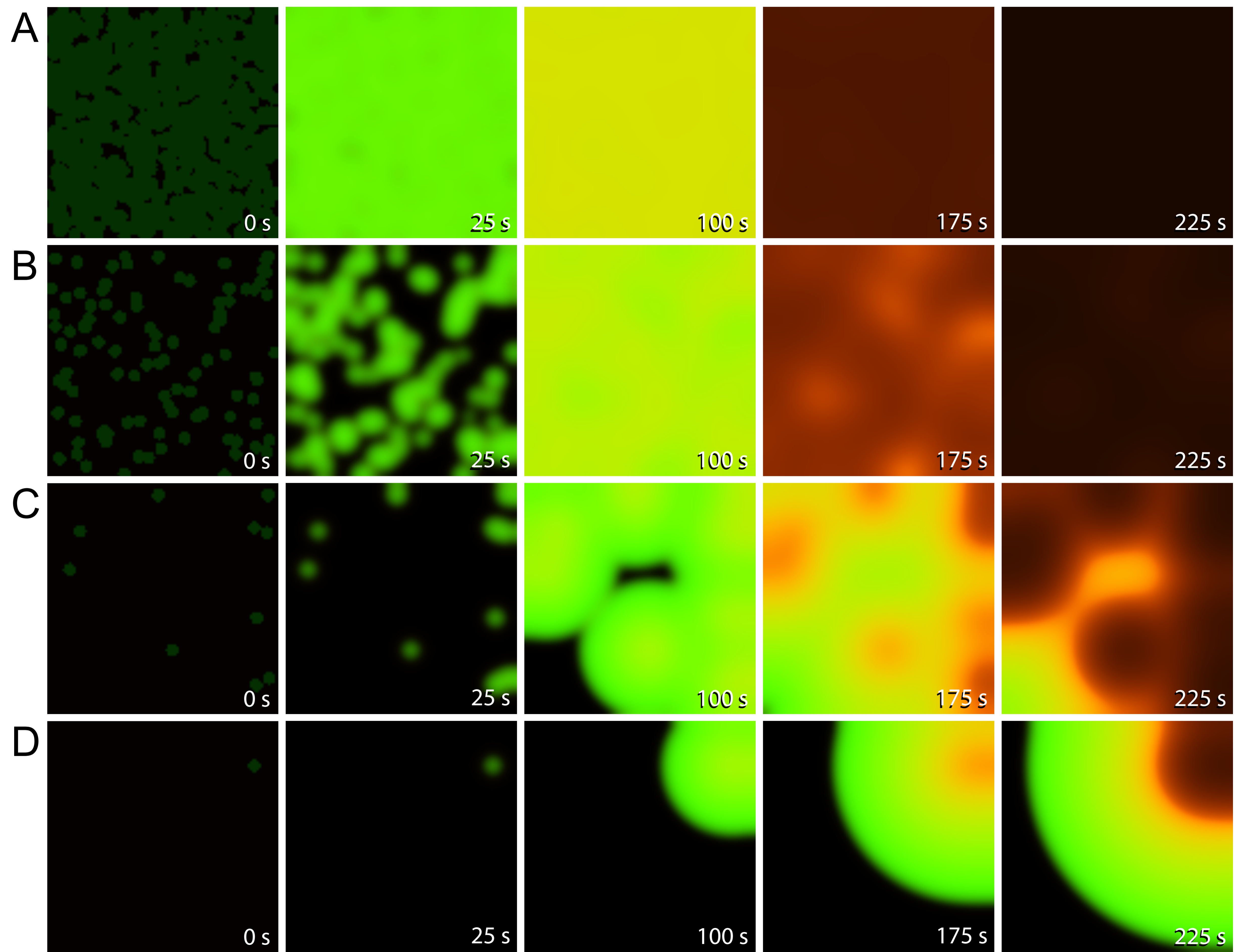

Supplement: S14 Fig — Solutions to the RD-AABSM with Ni = 1000, 100, 10, and 1 initiation zones at time t = 0 s are shown in Panels A, B, C, and D at the displayed times. The solution to the RD-AABSM with Ni = 1000 at t = 0 s, Ni = 100 at t = 500 s, Ni = 10 at t = 1000 s, and Ni = 1 at t = 1500 s is essentially the same as shown in Panels A, B, C, and D. Color and spatial scales are as described in S11 Fig. (PDF) [file pcbi.1011615.s015.pdf]

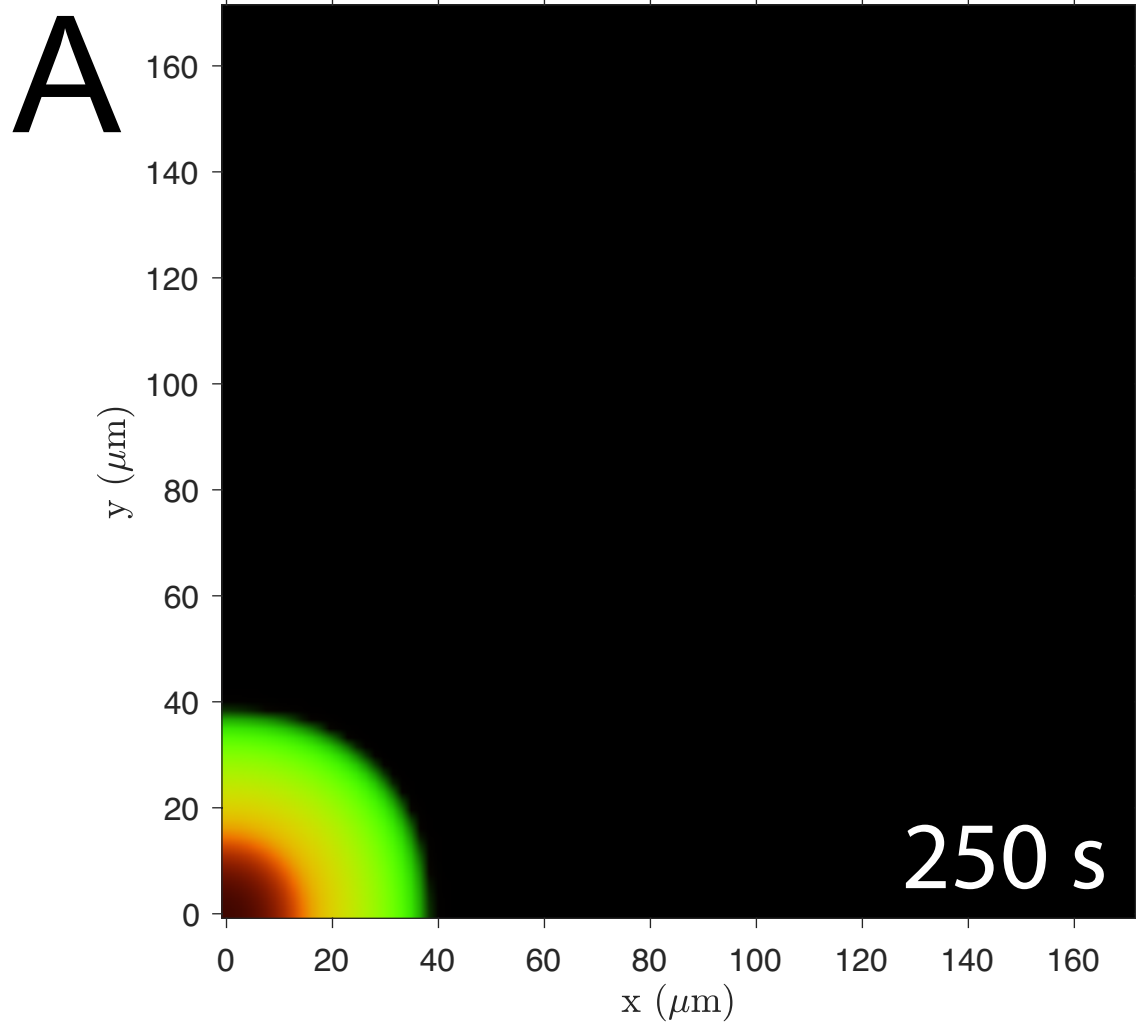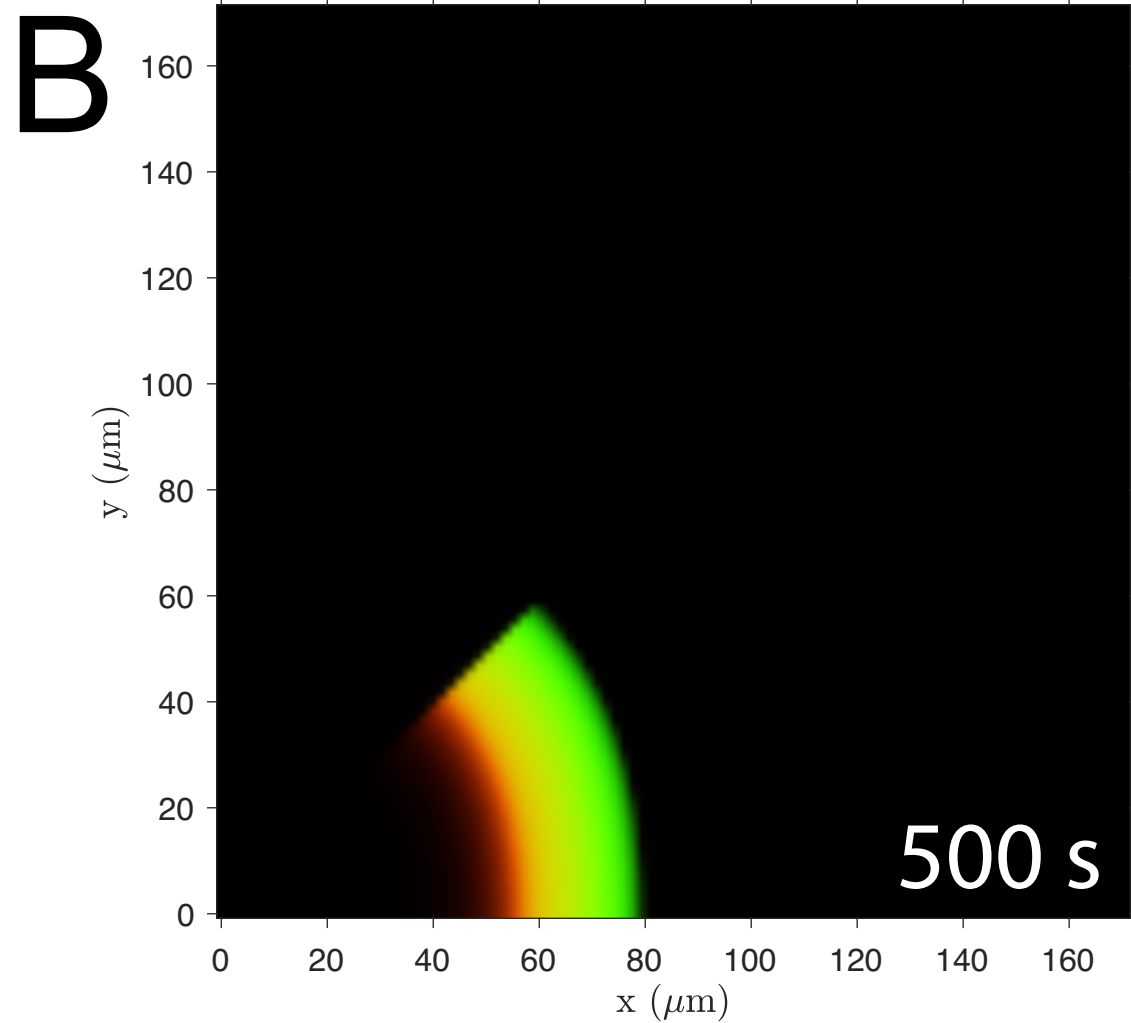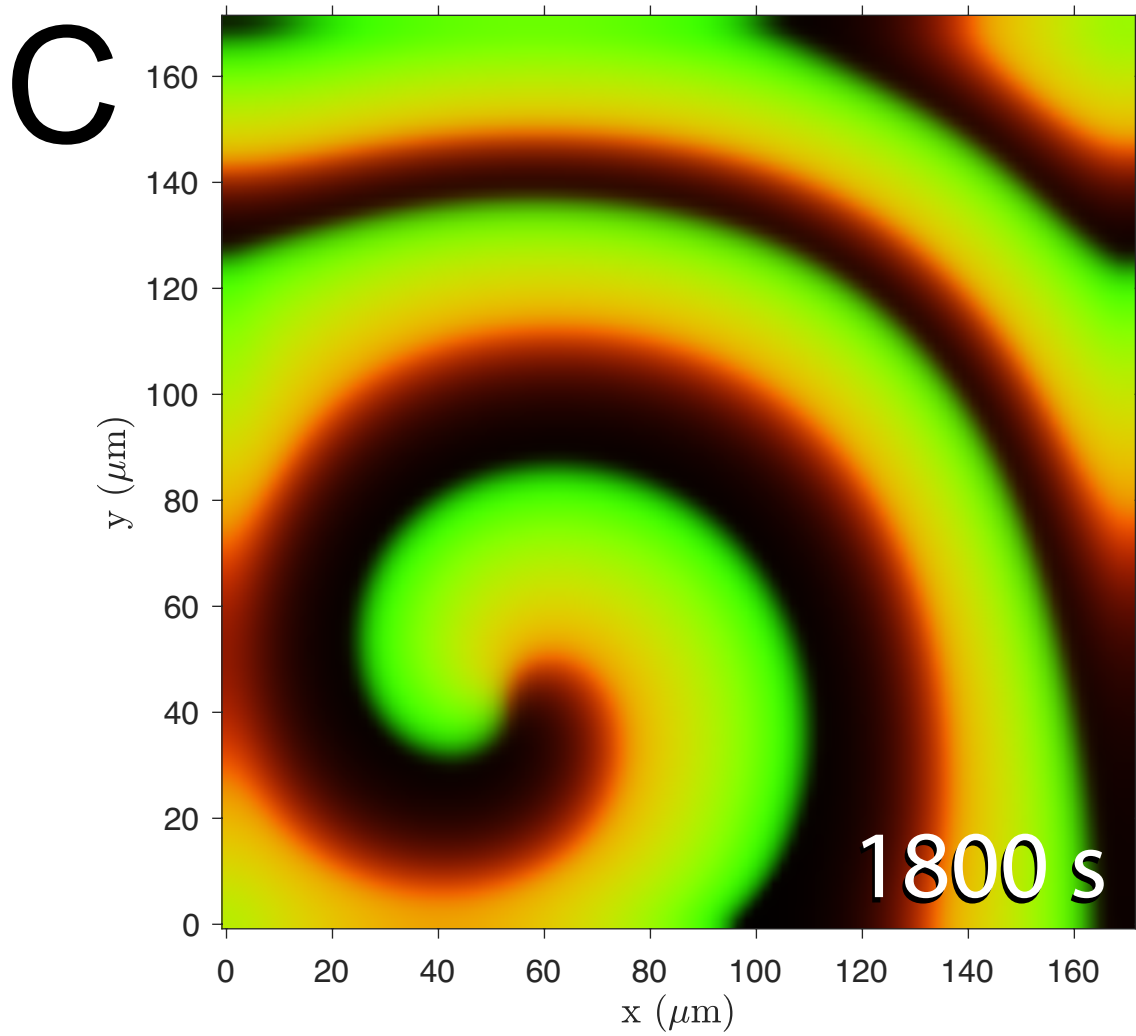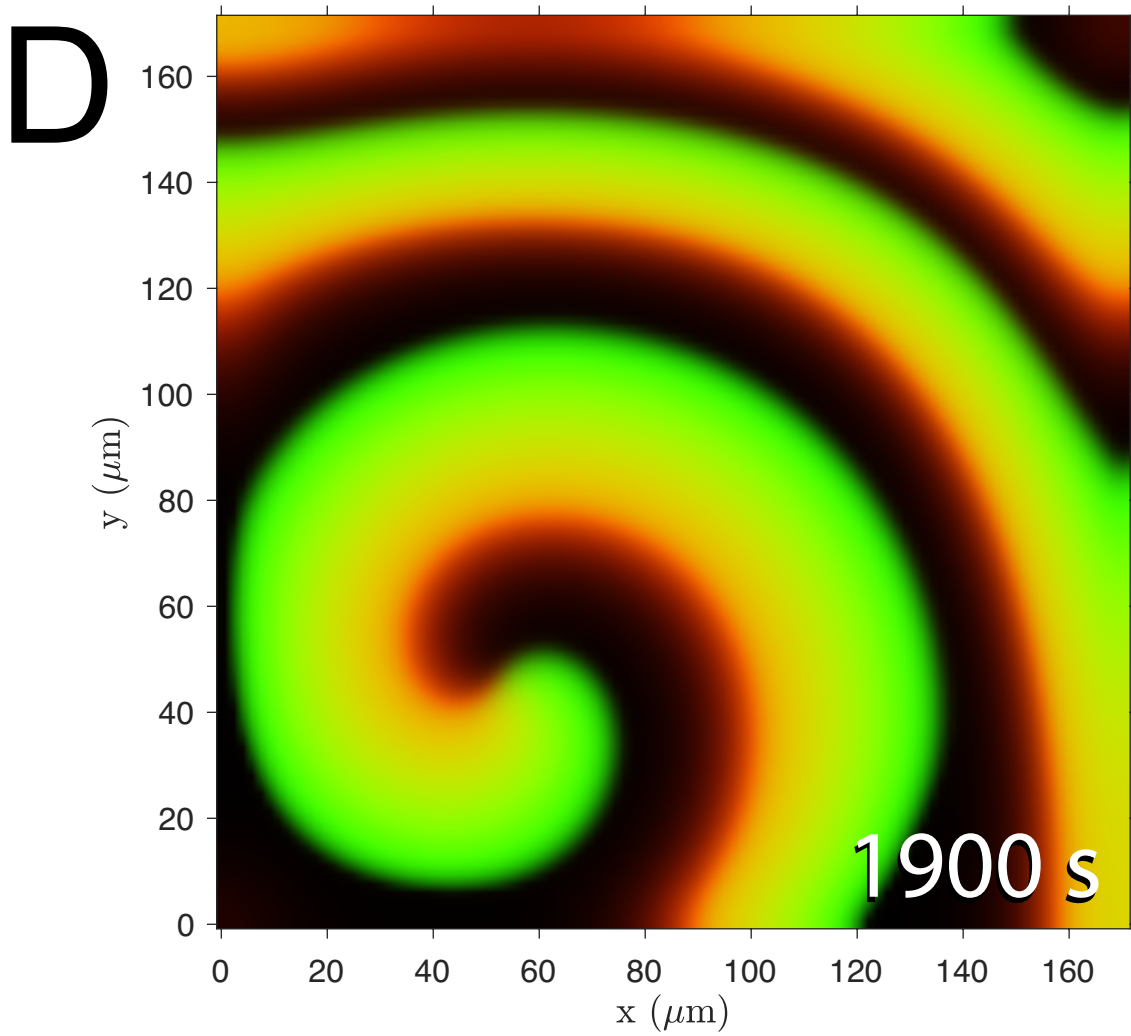

Supplement: S15 Fig — Starting from a single initiation point at (x, y) = (0, 0) μm at time t = 0 s with a radius of 2 ⋅ 2.40 μm, a traveling wave emerges (Panel A). Breaking the symmetry in the traveling wave (Panel B), a spiral wave emerges and persists, as shown in Panel C and roughly half a rotation later in Panel D. Simulation times are displayed, MinD and MinE are shown in green and red on the scale of MinD and MinE in the oscillation data, and the height of each square shown is twice that of the microscopy images in Ivanov and Mizuuchi’s experiments. (PDF) [file pcbi.1011615.s016.pdf]

**A**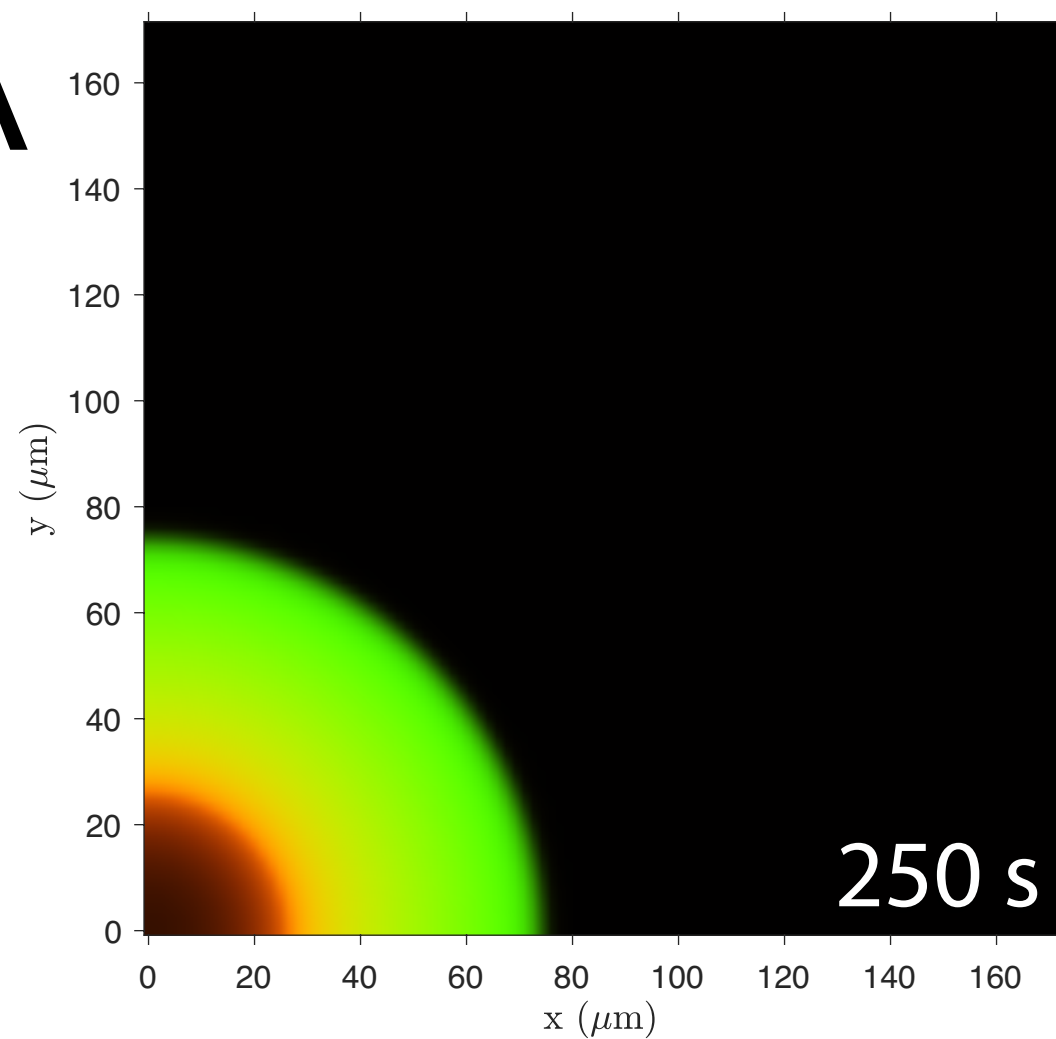**B**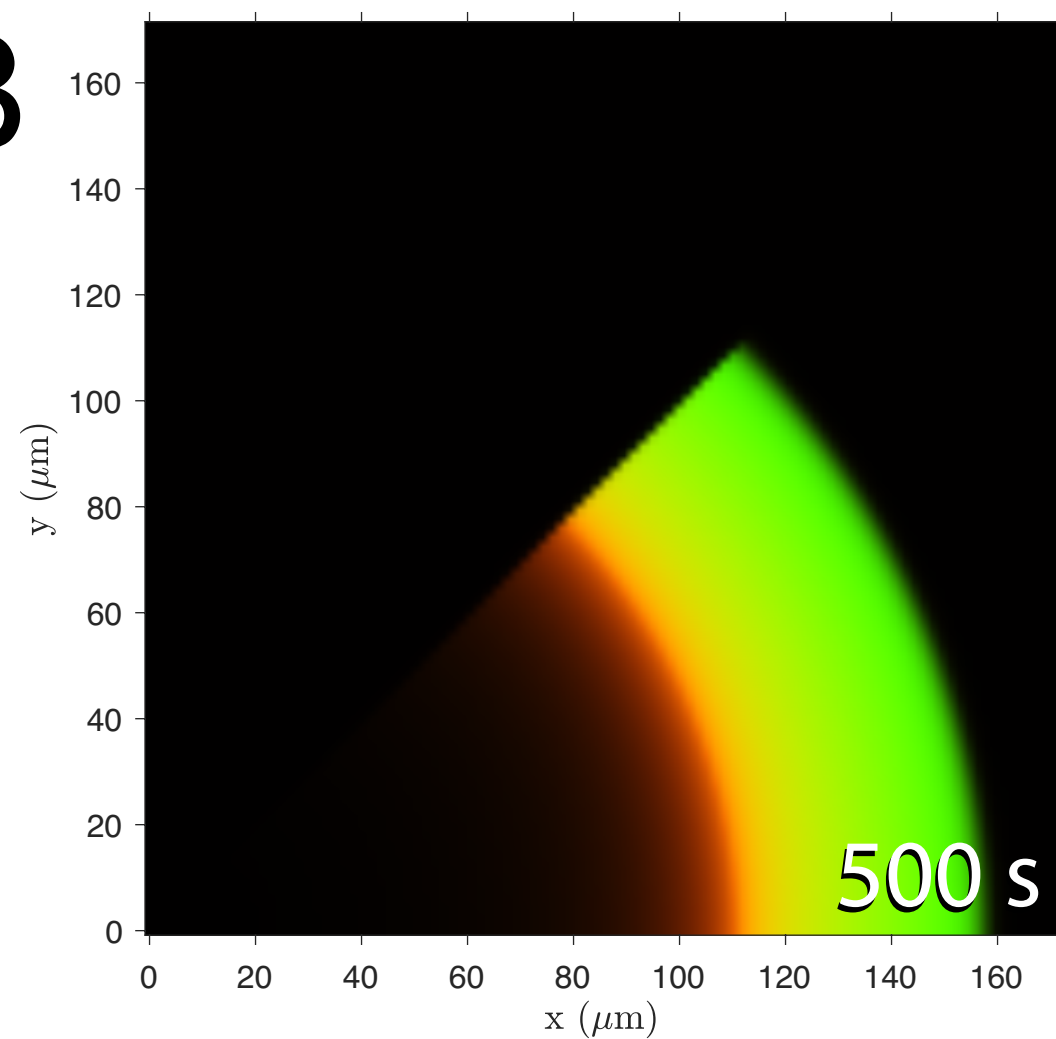**C**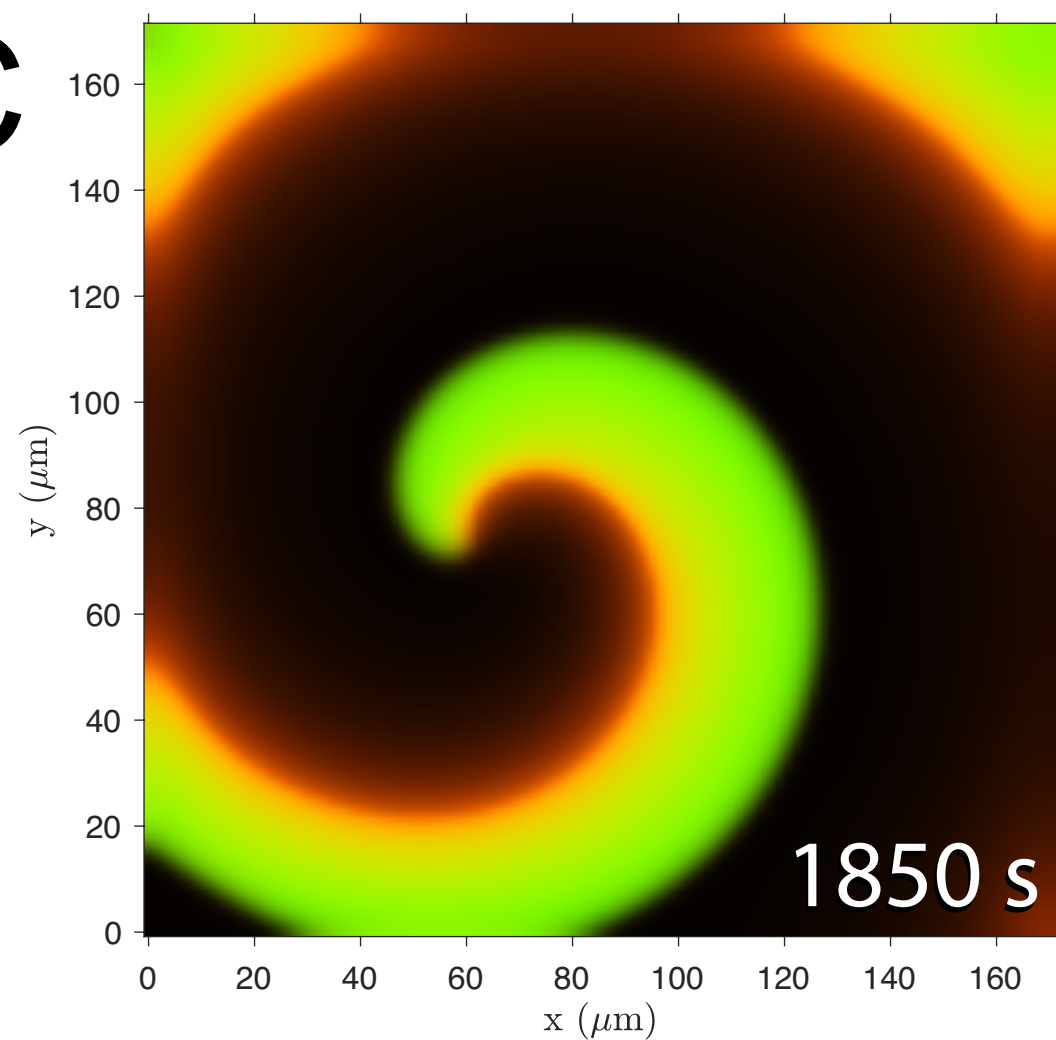**D**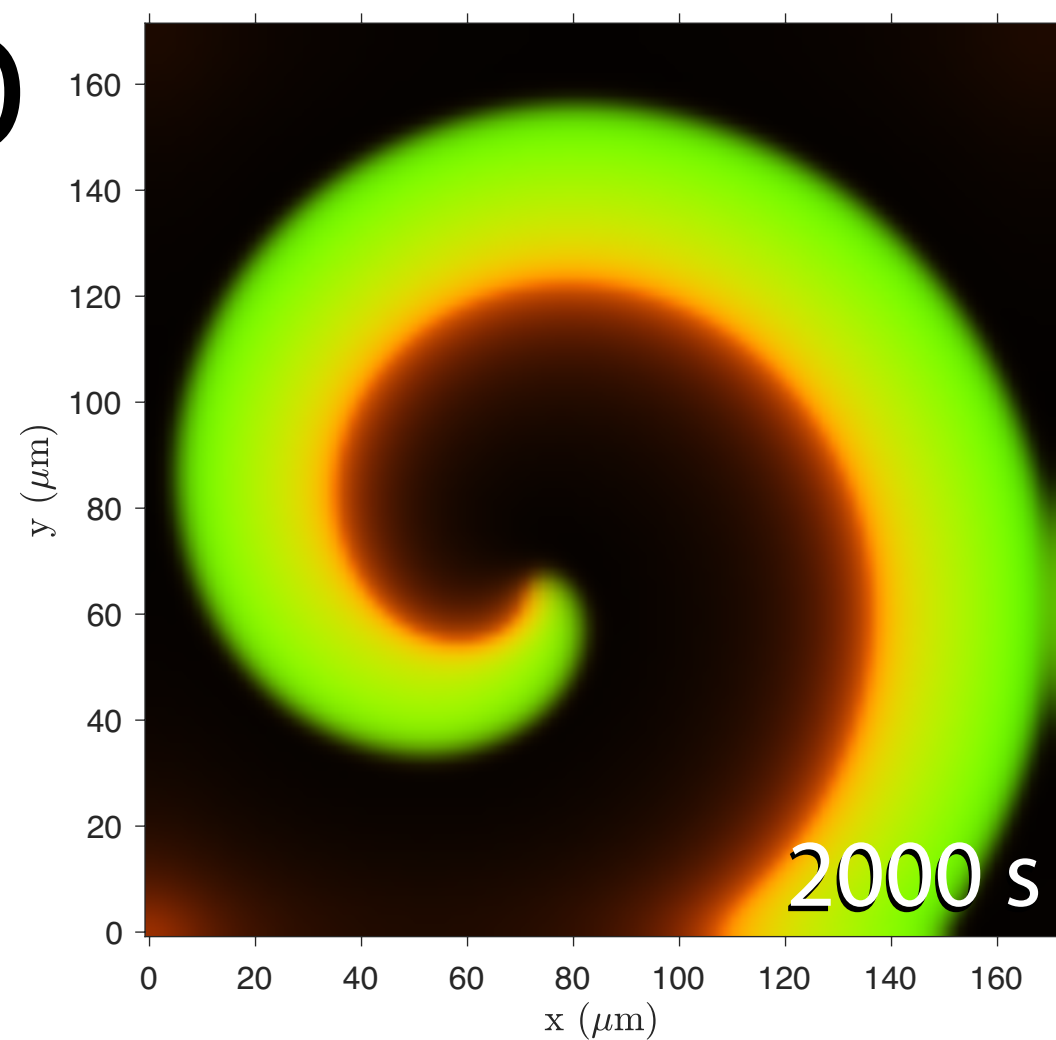

Supplement: S16 Fig — Starting from a single initiation point at (x, y) = (0, 0) μm at time t = 0 s with a radius of 2.40 μm, a traveling wave emerges (Panel A). As in S15 Fig, breaking the symmetry in the traveling wave (Panel B), a spiral wave emerges and persists, as shown in Panel C and roughly half a rotation later in Panel D. Simulation times are displayed, and the color and spatial scales are as described in S15 Fig. (PDF) [file pcbi.1011615.s017.pdf]
