# Supplementary material for: The mechanism of MinD stability modulation by MinE in Min protein dynamics
Source: PLoS Comput Biol. 2023 Nov 17;19(11):e1011615. doi: 10.1371/journal.pcbi.1011615 (PMC10691731; doi:10.1371/journal.pcbi.1011615)
Supplement: S1 Table — An asterisk (*) indicates that the model includes MinD-membrane-dissociation reactions of the membrane-stable MinD state, the reactions de → D+E and de → D+e for the SAM and the reactions ded → D+E+D, ded → D+E+d, ded → D+de, ded → D+e+D, and ded → D+e+d for the AABSM. χ2 is the weighted sum of squared residuals, and AIC is the Akaike information criterion. χmin2 is the minimum χ2 value amongst the models, and AICmin is the minimum AIC value amongst the models. The AABSM without the inclusion of MinD-membrane-dissociation reactions of the membrane-stable MinD state fits the oscillation data and the MinD dissociation data better than the SAM, even with the inclusion of MinD-membrane-dissociation reactions of the membrane-stable MinD state in the SAM. Including MinD-membrane-dissociation reactions of the membrane-stable MinD state in the AABSM does not appreciably improve its fit to the oscillation data and does not improve its fit to the MinD dissociation data. We include this table as justification for the omission of the membrane-stable MinD dissociation reactions from the SAM and AABSM as mentioned in the Materials and Methods. (PDF) [file pcbi.1011615.s018.pdf]

	Oscillation Data		MinD Dissociation Data	
	$\chi^2/\chi^2_{\mathrm{min}}$	$\mathrm{AIC} - \mathrm{AIC}_{\mathrm{min}}$	$\chi^2/\chi^2_{\mathrm{min}}$	$\mathrm{AIC} - \mathrm{AIC}_{\mathrm{min}}$
SAM	1.9	$2.1 \cdot 10^2$	3.2	$3.6 \cdot 10^2$
SAM*	1.5	$1.3 \cdot 10^2$	3.2	$3.5 \cdot 10^2$
AABSM	1.1	$1.2 \cdot 10^1$	1.0	0
AABSM*	1	0	1	9.3

Table S1: Model Comparison with and without MinD-membrane-dissociation reactions of membrane-stable MinD states. An asterisk (*) indicates that the model includes MinD-membrane-dissociation reactions of the membrane-stable MinD state, the reactions de → D+E and de → D+e for the SAM and the reactions ded → D+E+D, ded → D+E+d, ded → D+de, ded → D+e+D, and ded → D+e+d for the AABSM. χ^2 is the weighted sum of squared residuals, and AIC is the Akaike information criterion. χ^2_{min} is the minimum χ^2 value amongst the models, and $\mathrm{AIC}_{\mathrm{min}}$ is the minimum AIC value amongst the models. The AABSM without the inclusion of MinD-membrane-dissociation reactions of the membrane-stable MinD state fits the oscillation data and the MinD dissociation data better than the SAM, even with the inclusion of MinD-membrane-dissociation reactions of the membrane-stable MinD state in the SAM. Including MinD-membrane-dissociation reactions of the membrane-stable MinD state in the AABSM does not appreciably improve its fit to the oscillation data and does not improve its fit to the MinD dissociation data. We include this table as justification for the omission of the membrane-stable MinD dissociation reactions from the SAM and AABSM as mentioned in the Materials and Methods.
