# Supplementary material for: The mechanism of MinD stability modulation by MinE in Min protein dynamics
Source: PLoS Comput Biol. 2023 Nov 17;19(11):e1011615. doi: 10.1371/journal.pcbi.1011615 (PMC10691731; doi:10.1371/journal.pcbi.1011615)
Supplement: S2 Table — The time course data is subject to inherent experimental and methodological error in density calibration. As such, we perturb the time course data by a range of scaling factors and fit the SAM and the AABSM to the perturbed data sets to compare how well the models can fit the time course data over a range of density calibration values. When and only when MinD and MinE are rescaled in tandem, both the SAM and the AABSM (as well as the nested CAAM and AAM) can be rescaled to accommodate the change without affecting the quality of the fits to the data. I.e., the quality of the fits of the SAM and the AABSM to the time course data depend only on the relative density calibration of MinD and MinE, not the density calibration value of each. In accordance, we scale MinE time course data by ϵ = 0.8, 0.9, 1, 1.1, 1, 2, leave MinD time course data unchanged, and fit the SAM and the AABSM to the perturbed time course data—a fit of the models to the time course data with ±0, 10, 20% MinE-to-MinD relative density calibration error of that measured experimentally. Results are shown above, with χ2 and χmin2 as defined in S1 Table. For both the oscillation data and the MinD dissociation data, the AABSM outperforms the SAM in fitting (as measured by χ2) for all density calibration perturbations (values of ϵ). A larger perturbation in density calibration from that measured experimentally (a larger deviation from ϵ = 1) results in a decrease in the quality of the fit of the AABSM to the time course data (a larger value of χ2), apart from a minor exception at ϵ = 1.2 for the MinD dissociation data. (PDF) [file pcbi.1011615.s019.pdf]

	Oscillation Data					MinD Dissociation Data				
	$\epsilon = 0.8$	$\epsilon = 0.9$	$\epsilon = 1.0$	$\epsilon = 1.1$	$\epsilon = 1.2$	$\epsilon = 0.8$	$\epsilon = 0.9$	$\epsilon = 1.0$	$\epsilon = 1.1$	$\epsilon = 1.2$
SAM $(\chi^2/\chi^2_{\min})$	1.36	1.82	1.83	1.26	1.03	3.24	3.19	3.23	2.86	2.74
AABSM $(\chi^2/\chi^2_{\min})$	1	1	1	1	1	1	1	1	1	1
$\chi^2_{\min}/10^{-4}$	1.93	1.34	1.27	1.82	2.39	2.18	1.90	1.64	1.67	1.59

Table S2: Comparing the fits of the SAM and the AABSM to the time course data with perturbations. The time course data is subject to inherent experimental and methodological error in density calibration. As such, we perturb the time course data by a range of scaling factors and fit the SAM and the AABSM to the perturbed data sets to compare how well the models can fit the time course data over a range of density calibration values. When and only when MinD and MinE are rescaled in tandem, both the SAM and the AABSM (as well as the nested CAAM and AAM) can be rescaled to accommodate the change without affecting the quality of the fits to the data. I.e., the quality of the fits of the SAM and the AABSM to the time course data depend only on the relative density calibration of MinD and MinE, not the density calibration value of each. In accordance, we scale MinE time course data by $\epsilon = 0.8, 0.9, 1, 1.1, 1, 2$, leave MinD time course data unchanged, and fit the SAM and the AABSM to the perturbed time course data – a fit of the models to the time course data with $\pm 0, 10, 20\%$ MinE-to-MinD relative density calibration error of that measured experimentally. Results are shown above, with χ^2 and $\chi^2_{\min}$ as defined in Table **??**. For both the oscillation data and the MinD dissociation data, the AABSM outperforms the SAM in fitting (as measured by χ^2) for all density calibration perturbations (values of ϵ). A larger perturbation in density calibration from that measured experimentally (a larger deviation from $\epsilon = 1$) results in a decrease in the quality of the fit of the AABSM to the time course data (a larger value of χ^2), apart from a minor exception at $\epsilon = 1.2$ for the MinD dissociation data.
