# Supplementary material for: The mechanism of MinD stability modulation by MinE in Min protein dynamics
Source: PLoS Comput Biol. 2023 Nov 17;19(11):e1011615. doi: 10.1371/journal.pcbi.1011615 (PMC10691731; doi:10.1371/journal.pcbi.1011615)
Supplement: S3 Table — (PDF) [file pcbi.1011615.s020.pdf]

	Oscillation Data		MinD Dissociation Data		
Parameter	Value	95% Confidence Interval	Value	95% Confidence Interval	Units
C_d	$3.0 \cdot 10^2$	$[2.8 \cdot 10^2, 3.0 \cdot 10^2]$	0	$[0, 0]$	μm^{-2}
C_d (w/o MinE)			$5.1 \cdot 10^1$	$[0, 5.1 \cdot 10^1]$	μm^{-2}
C_e	$2.3 \cdot 10^2$	$[1.6 \cdot 10^2, 2.3 \cdot 10^2]$	0	$[0, 3.9 \cdot 10^1]$	μm^{-2}
$c_{\bar{d}}$	0	$[0, 2.4 \cdot 10^1]$	0	$[0, 0]$	μm^{-2}
$c_{\bar{d}}$ (w/o MinE)			0	$[0, 2.5 \cdot 10^1]$	μm^{-2}
$c_{\max}$	$5.5 \cdot 10^3$	$[5.4 \cdot 10^3, 5.6 \cdot 10^3]$			μm^{-2}
c_s	$9.4 \cdot 10^1$	$[5.8 \cdot 10^1, 1.6 \cdot 10^2]$	$1.5 \cdot 10^3$	$[1.3 \cdot 10^3, 1.9 \cdot 10^3]$	μm^{-2}
n_s	7.6	$[2.9, 1.0 \cdot 10^1]$	2.3	$[1.9, 3.2]$	
$\omega_{D \to d}$	$1.0 \cdot 10^1$	$[8.5, 1.0 \cdot 10^1]$			$\mu m^{-2}\ s^{-1}$
$\omega^d_{D \to d}$	$2.4 \cdot 10^{-1}$	$[2.2 \cdot 10^{-1}, 2.5 \cdot 10^{-1}]$			s^{-1}
$\omega_{E,d \to de}$	$6.1 \cdot 10^{-3}$	$[5.7 \cdot 10^{-3}, 6.5 \cdot 10^{-3}]$	$1.0 \cdot 10^{-2}$	$[1.0 \cdot 10^{-2}, 1.1 \cdot 10^{-2}]$	s^{-1}
$\omega_{d,e \to de}$	$3.2 \cdot 10^{-1}$	$[3.1 \cdot 10^{-1}, 3.3 \cdot 10^{-1}]$	9.9	$[9.7, 1.0 \cdot 10^1]$	$\mu m^2\ s^{-1}$
$\omega_{d \to D}$	$1.0 \cdot 10^1$	$[1.5, 1.0 \cdot 10^1]$	$2.1 \cdot 10^{-1}$	$[1.7 \cdot 10^{-1}, 2.5 \cdot 10^{-1}]$	s^{-1}
$\omega_{de \to D,E}$	$1.7 \cdot 10^{-3}$	$[1.0 \cdot 10^{-3}, 2.4 \cdot 10^{-3}]$	0	$[0, 3.1 \cdot 10^{-3}]$	s^{-1}
$\omega_{de \to D,e}$	$7.5 \cdot 10^{-2}$	$[6.9 \cdot 10^{-2}, 7.7 \cdot 10^{-2}]$	$6.5 \cdot 10^{-2}$	$[6.0 \cdot 10^{-2}, 7.0 \cdot 10^{-2}]$	s^{-1}
$\omega_{e \to E}$	$6.2 \cdot 10^{-2}$	$[5.5 \cdot 10^{-2}, 7.0 \cdot 10^{-2}]$	$4.3 \cdot 10^{-1}$	$[3.8 \cdot 10^{-1}, 4.5 \cdot 10^{-1}]$	s^{-1}

Table S3: Parameters from the fits of the AAM to the oscillation data and the MinD dissociation data.
