# Supplementary material for: The mechanism of MinD stability modulation by MinE in Min protein dynamics
Source: PLoS Comput Biol. 2023 Nov 17;19(11):e1011615. doi: 10.1371/journal.pcbi.1011615 (PMC10691731; doi:10.1371/journal.pcbi.1011615)
Supplement: S4 Table — (PDF) [file pcbi.1011615.s021.pdf]

	Oscillation Data		MinD Dissociation Data		
Parameter	Value	95% Confidence Interval	Value	95% Confidence Interval	Units
C_d	$2.6 \cdot 10^2$	$[2.3 \cdot 10^2, 3.0 \cdot 10^2]$	$1.2 \cdot 10^1$	$[0, 2.7 \cdot 10^1]$	μm^{-2}
C_d (w/o MinE)			$5.1 \cdot 10^1$	$[1.7 \cdot 10^{-1}, 5.1 \cdot 10^1]$	μm^{-2}
C_e	0	$[0, 2.0 \cdot 10^1]$	$3.8 \cdot 10^1$	$[1.4 \cdot 10^1, 2.4 \cdot 10^1]$	μm^{-2}
$c_{\bar{d}}$	$5.3 \cdot 10^1$	$[5.7, 1.1 \cdot 10^2]$	6.0	$[0, 1.2 \cdot 10^1]$	μm^{-2}
$c_{\bar{d}}$ (w/o MinE)			0	$[0, 1.7 \cdot 10^1]$	μm^{-2}
$c_{\max}$	$6.7 \cdot 10^3$	$[6.7 \cdot 10^3, 6.7 \cdot 10^3]$			μm^{-2}
c_s	$4.5 \cdot 10^3$	$[4.5 \cdot 10^3, 4.5 \cdot 10^3]$	$1.7 \cdot 10^3$	$[1.4 \cdot 10^3, 1.9 \cdot 10^3]$	μm^{-2}
n_s	4.0	$[4.0, 4.1]$	1.7	$[1.5, 2.1]$	
$\omega_{D \to d}$	0	$[0, 6.9 \cdot 10^{-2}]$			$\mu m^{-2}\ s^{-1}$
$\omega^d_{D \to d}$	4.2	$[4.2, 4.3]$			s^{-1}
$\omega^{de}_{D \to d}$	$7.0 \cdot 10^{-2}$	$[6.3 \cdot 10^{-2}, 7.5 \cdot 10^{-2}]$			s^{-1}
$\omega_{E,d \to de}$	$3.2 \cdot 10^{-3}$	$[2.5 \cdot 10^{-3}, 3.5 \cdot 10^{-3}]$	$3.8 \cdot 10^{-3}$	$[3.1 \cdot 10^{-3}, 4.0 \cdot 10^{-3}]$	s^{-1}
$\omega^{de}_{E,d \to de}$	0	$[0, 8.5 \cdot 10^{-7}]$	$3.8 \cdot 10^{-6}$	$[5.7 \cdot 10^{-7}, 8.7 \cdot 10^{-6}]$	$\mu m^2\ s^{-1}$
$\omega^e_{E,d \to de}$	$3.1 \cdot 10^{-2}$	$[2.9 \cdot 10^{-2}, 3.2 \cdot 10^{-2}]$	$5.9 \cdot 10^{-2}$	$[5.8 \cdot 10^{-2}, 6.4 \cdot 10^{-2}]$	$\mu m^2\ s^{-1}$
$\omega_{d,e \to de}$	$4.1 \cdot 10^{-3}$	$[3.8 \cdot 10^{-3}, 4.4 \cdot 10^{-3}]$	6.4	$[6.4, 6.4]$	$\mu m^2\ s^{-1}$
$\omega_{d \to D}$	2.6	$[2.6, 2.6]$	$2.0 \cdot 10^{-1}$	$[1.8 \cdot 10^{-1}, 2.4 \cdot 10^{-1}]$	s^{-1}
$\omega_{de \to D,E}$	$9.6 \cdot 10^{-2}$	$[9.1 \cdot 10^{-2}, 1.0 \cdot 10^{-1}]$	$2.9 \cdot 10^{-7}$	$[0, 5.4 \cdot 10^{-3}]$	s^{-1}
$\omega_{de \to D,e}$	$1.3 \cdot 10^{-2}$	$[1.2 \cdot 10^{-2}, 1.4 \cdot 10^{-2}]$	$7.7 \cdot 10^{-2}$	$[7.2 \cdot 10^{-2}, 7.7 \cdot 10^{-2}]$	s^{-1}
$\omega_{de \to d,e}$	0	$[0, 3.3 \cdot 10^{-4}]$	7.1	$[7.1, 7.1]$	s^{-1}
$\omega_{e \to E}$	$4.7 \cdot 10^{-3}$	$[4.3 \cdot 10^{-3}, 5.0 \cdot 10^{-3}]$	$3.3 \cdot 10^{-1}$	$[3.2 \cdot 10^{-1}, 3.3 \cdot 10^{-1}]$	s^{-1}

Table S4: Parameters from the fits of the CAAM to the oscillation data and the MinD dissociation data.
