# Supplementary material for: The mechanism of MinD stability modulation by MinE in Min protein dynamics
Source: PLoS Comput Biol. 2023 Nov 17;19(11):e1011615. doi: 10.1371/journal.pcbi.1011615 (PMC10691731; doi:10.1371/journal.pcbi.1011615)
Supplement: S5 Table — (PDF) [file pcbi.1011615.s022.pdf]

	Oscillation Data		MinD Dissociation Data		
Parameter	Value	95% Confidence Interval	Value	95% Confidence Interval	Units
C_d	$3.0 \cdot 10^2$	$[2.8 \cdot 10^2, 3.0 \cdot 10^2]$	$1.7 \cdot 10^2$	$[6.6 \cdot 10^1, 1.3 \cdot 10^2]$	μm^{-2}
C_d (w/o MinE)			$5.1 \cdot 10^1$	$[1.2 \cdot 10^1, 5.0 \cdot 10^1]$	μm^{-2}
C_e	$2.3 \cdot 10^2$	$[2.1 \cdot 10^2, 2.3 \cdot 10^2]$	1.1	$[0, 4.6]$	μm^{-2}
$c_{\bar{d}}$	$1.5 \cdot 10^2$	$[1.3 \cdot 10^2, 1.5 \cdot 10^2]$	$1.0 \cdot 10^2$	$[3.2 \cdot 10^1, 6.5 \cdot 10^1]$	μm^{-2}
$c_{\bar{d}}$ (w/o MinE)			0	$[0, 0]$	μm^{-2}
$c_{\max}$	$5.2 \cdot 10^3$	$[5.2 \cdot 10^3, 5.2 \cdot 10^3]$			μm^{-2}
c_s	$4.5 \cdot 10^2$	$[4.2 \cdot 10^2, 4.7 \cdot 10^2]$	$1.8 \cdot 10^3$	$[1.5 \cdot 10^3, 2.0 \cdot 10^3]$	μm^{-2}
n_s	3.9	$[3.2, 4.4]$	1.6	$[1.4, 1.9]$	
$\omega_{D\to d}$	0	$[0, 1.8 \cdot 10^{-1}]$			$\mu m^{-2}\ s^{-1}$
$\omega^{d}_{D\to d}$	$2.5 \cdot 10^{-1}$	$[2.3 \cdot 10^{-1}, 2.6 \cdot 10^{-1}]$			s^{-1}
$\omega^{de}_{D\to d}$	$7.8 \cdot 10^{-1}$	$[7.3 \cdot 10^{-1}, 8.5 \cdot 10^{-1}]$			s^{-1}
$\omega^{ede}_{D\to d}$	$7.9 \cdot 10^{-1}$	$[7.8 \cdot 10^{-1}, 8.0 \cdot 10^{-1}]$			s^{-1}
$\omega_{E,d\to de}$	$1.2 \cdot 10^{-3}$	$[2.2 \cdot 10^{-4}, 1.5 \cdot 10^{-3}]$	$3.6 \cdot 10^{-5}$	$[0, 1.4 \cdot 10^{-3}]$	s^{-1}
$\omega^{de}_{E,d\to de}$	0	$[0, 7.5 \cdot 10^{-7}]$	$5.3 \cdot 10^{-5}$	$[3.1 \cdot 10^{-5}, 5.5 \cdot 10^{-5}]$	$\mu m^2\ s^{-1}$
$\omega^{e}_{E,d\to de}$	$9.3 \cdot 10^{-1}$	$[9.0 \cdot 10^{-1}, 9.5 \cdot 10^{-1}]$	$1.7 \cdot 10^{-3}$	$[8.4 \cdot 10^{-4}, 2.4 \cdot 10^{-3}]$	$\mu m^2\ s^{-1}$
$\omega^{ede}_{E,d\to de}$	0	$[0, 3.2 \cdot 10^{-6}]$	$1.7 \cdot 10^{-5}$	$[0, 5.8 \cdot 10^{-3}]$	$\mu m^2\ s^{-1}$
$\omega_{E,de\to ede}$	0	$[0, 4.7 \cdot 10^{-5}]$	$7.5 \cdot 10^{-6}$	$[0, 5.0 \cdot 10^{-4}]$	s^{-1}
$\omega^{de}_{E,de\to ede}$	0	$[0, 4.4 \cdot 10^{-7}]$	$9.3 \cdot 10^{-6}$	$[0, 1.8 \cdot 10^{-5}]$	$\mu m^2\ s^{-1}$
$\omega^{e}_{E,de\to ede}$	9.9	$[9.9, 1.0 \cdot 10^1]$	$1.8 \cdot 10^{-12}$	$[0, 1.5 \cdot 10^{-4}]$	$\mu m^2\ s^{-1}$
$\omega^{ede}_{E,de\to ede}$	$5.3 \cdot 10^{-9}$	$[0, 7.0 \cdot 10^{-6}]$	$3.1 \cdot 10^{-3}$	$[2.3 \cdot 10^{-3}, 3.4 \cdot 10^{-3}]$	$\mu m^2\ s^{-1}$
$\omega_{d,e\to de}$	$1.4 \cdot 10^{-4}$	$[1.1 \cdot 10^{-4}, 1.6 \cdot 10^{-4}]$	$1.9 \cdot 10^{-4}$	$[1.1 \cdot 10^{-4}, 4.6 \cdot 10^{-4}]$	$\mu m^2\ s^{-1}$
$\omega_{d,ede\to de,de}$	$2.3 \cdot 10^{-4}$	$[2.3 \cdot 10^{-4}, 2.3 \cdot 10^{-4}]$	$3.9 \cdot 10^{-4}$	$[0, 2.0 \cdot 10^{-3}]$	$\mu m^2\ s^{-1}$
$\omega_{d\to D}$	$2.8 \cdot 10^{-1}$	$[2.7 \cdot 10^{-1}, 3.2 \cdot 10^{-1}]$	$2.1 \cdot 10^{-1}$	$[1.9 \cdot 10^{-1}, 2.3 \cdot 10^{-1}]$	s^{-1}
$\omega_{de,de\to d,ede}$	$3.1 \cdot 10^{-5}$	$[2.9 \cdot 10^{-5}, 3.2 \cdot 10^{-5}]$	$6.1 \cdot 10^{-8}$	$[0, 3.3 \cdot 10^{-6}]$	$\mu m^2\ s^{-1}$
$\omega_{de,e\to ede}$	$3.7 \cdot 10^{-3}$	$[3.5 \cdot 10^{-3}, 4.0 \cdot 10^{-3}]$	$1.2 \cdot 10^{-3}$	$[1.1 \cdot 10^{-3}, 1.2 \cdot 10^{-3}]$	$\mu m^2\ s^{-1}$
$\omega_{de\to d,e}$	0	$[0, 3.2 \cdot 10^{-7}]$	$4.9 \cdot 10^{-4}$	$[0, 1.0 \cdot 10^{-2}]$	s^{-1}
$\omega_{e\to E}$	$4.1 \cdot 10^{-3}$	$[4.1 \cdot 10^{-3}, 4.4 \cdot 10^{-3}]$	$6.2 \cdot 10^{-2}$	$[6.0 \cdot 10^{-2}, 6.4 \cdot 10^{-2}]$	s^{-1}
$\omega_{ede\to D,e,e}$	$8.7 \cdot 10^{-5}$	$[8.1 \cdot 10^{-5}, 9.4 \cdot 10^{-5}]$	1.0	$[9.2 \cdot 10^{-1}, 1.2]$	s^{-1}
$\omega_{ede\to E,D,E}$	$7.9 \cdot 10^{-1}$	$[7.8 \cdot 10^{-1}, 8.0 \cdot 10^{-1}]$	$2.5 \cdot 10^{-1}$	$[2.0 \cdot 10^{-1}, 3.0 \cdot 10^{-1}]$	s^{-1}
$\omega_{ede\to E,D,e}$	$2.0 \cdot 10^{-4}$	$[1.8 \cdot 10^{-4}, 2.1 \cdot 10^{-4}]$	$7.2 \cdot 10^{-1}$	$[6.8 \cdot 10^{-1}, 8.0 \cdot 10^{-1}]$	s^{-1}
$\omega_{ede\to de,e}$	$1.7 \cdot 10^{-4}$	$[1.6 \cdot 10^{-4}, 1.8 \cdot 10^{-4}]$	1.1	$[1.0, 1.2]$	s^{-1}

Table S5: Parameters from the fits of the SAM to the oscillation data and the MinD dissociation data.
