# Supplementary material for: The mechanism of MinD stability modulation by MinE in Min protein dynamics
Source: PLoS Comput Biol. 2023 Nov 17;19(11):e1011615. doi: 10.1371/journal.pcbi.1011615 (PMC10691731; doi:10.1371/journal.pcbi.1011615)
Supplement: S6 Table — (PDF) [file pcbi.1011615.s023.pdf]

	Oscillation Data		MinD Dissociation Data		
Parameter	Value	95% Confidence Interval	Value	95% Confidence Interval	Units
C_d	$6.5 \cdot 10^1$	$[5.6 \cdot 10^1, 7.4 \cdot 10^1]$	$1.7 \cdot 10^2$	$[6.3 \cdot 10^1, 1.1 \cdot 10^2]$	μm^{-2}
C_d (w/o MinE)			$5.1 \cdot 10^1$	$[1.8 \cdot 10^1, 4.5 \cdot 10^1]$	μm^{-2}
C_e	5.4	$[1.4, 9.7]$	0	$[0, 7.3]$	μm^{-2}
$c_{\bar{d}}$	$1.9 \cdot 10^1$	$[1.5, 2.6 \cdot 10^1]$	$8.3 \cdot 10^1$	$[3.1 \cdot 10^1, 5.4 \cdot 10^1]$	μm^{-2}
$c_{\bar{d}}$ (w/o MinE)			0	$[0, 1.1]$	μm^{-2}
$c_{\max}$	$5.3 \cdot 10^3$	$[5.2 \cdot 10^3, 5.3 \cdot 10^3]$			μm^{-2}
c_s	$4.9 \cdot 10^2$	$[4.6 \cdot 10^2, 5.3 \cdot 10^2]$	$2.2 \cdot 10^3$	$[2.0 \cdot 10^3, 2.3 \cdot 10^3]$	μm^{-2}
n_s	9.1	$[5.4, 1.0 \cdot 10^1]$	1.9	$[1.7, 2.1]$	
$\omega_{D\to d}$	0	$[0, 7.3 \cdot 10^{-2}]$			$\mu m^{-2}\ s^{-1}$
$\omega^{d}_{D\to d}$	$3.5 \cdot 10^{-1}$	$[3.4 \cdot 10^{-1}, 3.5 \cdot 10^{-1}]$			s^{-1}
$\omega^{de}_{D\to d}$	$2.0 \cdot 10^{-1}$	$[2.0 \cdot 10^{-1}, 2.0 \cdot 10^{-1}]$			s^{-1}
$\omega^{ded}_{D\to d}$	$4.0 \cdot 10^{-1}$	$[3.9 \cdot 10^{-1}, 4.0 \cdot 10^{-1}]$			s^{-1}
$\omega_{E,d\to de}$	$4.0 \cdot 10^{-3}$	$[3.5 \cdot 10^{-3}, 4.1 \cdot 10^{-3}]$	$3.6 \cdot 10^{-3}$	$[3.1 \cdot 10^{-3}, 4.1 \cdot 10^{-3}]$	s^{-1}
$\omega^{de}_{E,d\to de}$	$1.3 \cdot 10^{-10}$	$[0, 1.2 \cdot 10^{-6}]$	$7.5 \cdot 10^{-5}$	$[0, 2.2 \cdot 10^{-4}]$	$\mu m^2\ s^{-1}$
$\omega^{ded}_{E,d\to de}$	0	$[0, 5.0 \cdot 10^{-7}]$	$3.6 \cdot 10^{-6}$	$[0, 1.3 \cdot 10^{-5}]$	$\mu m^2\ s^{-1}$
$\omega^{e}_{E,d\to de}$	1.0	$[1.0, 1.0]$	$4.0 \cdot 10^{-1}$	$[3.6 \cdot 10^{-1}, 4.4 \cdot 10^{-1}]$	$\mu m^2\ s^{-1}$
$\omega_{E,ded\to de,de}$	$2.3 \cdot 10^{-6}$	$[0, 4.6 \cdot 10^{-4}]$	$7.7 \cdot 10^{-2}$	$[6.4 \cdot 10^{-2}, 8.0 \cdot 10^{-2}]$	s^{-1}
$\omega^{de}_{E,ded\to de,de}$	$1.4 \cdot 10^{-5}$	$[1.4 \cdot 10^{-5}, 1.5 \cdot 10^{-5}]$	$6.6 \cdot 10^{-4}$	$[5.6 \cdot 10^{-4}, 7.4 \cdot 10^{-4}]$	$\mu m^2\ s^{-1}$
$\omega^{ded}_{E,ded\to de,de}$	$1.6 \cdot 10^{-5}$	$[1.5 \cdot 10^{-5}, 1.6 \cdot 10^{-5}]$	$6.4 \cdot 10^{-9}$	$[0, 3.7 \cdot 10^{-5}]$	$\mu m^2\ s^{-1}$
$\omega^{e}_{E,ded\to de,de}$	$8.5 \cdot 10^{-2}$	$[8.5 \cdot 10^{-2}, 8.6 \cdot 10^{-2}]$	$4.5 \cdot 10^{-3}$	$[3.8 \cdot 10^{-3}, 5.3 \cdot 10^{-3}]$	$\mu m^2\ s^{-1}$
$\omega_{d,de\to ded}$	$7.2 \cdot 10^{-5}$	$[7.0 \cdot 10^{-5}, 7.3 \cdot 10^{-5}]$	$1.1 \cdot 10^{-1}$	$[1.1 \cdot 10^{-1}, 1.1 \cdot 10^{-1}]$	$\mu m^2\ s^{-1}$
$\omega_{d,e\to de}$	$3.0 \cdot 10^{-1}$	$[3.0 \cdot 10^{-1}, 3.1 \cdot 10^{-1}]$	3.4	$[3.4, 3.4]$	$\mu m^2\ s^{-1}$
$\omega_{d\to D}$	$2.5 \cdot 10^{-1}$	$[2.4 \cdot 10^{-1}, 2.6 \cdot 10^{-1}]$	$1.9 \cdot 10^{-1}$	$[1.8 \cdot 10^{-1}, 2.0 \cdot 10^{-1}]$	s^{-1}
$\omega_{de,de\to ded,e}$	$1.5 \cdot 10^{-3}$	$[1.5 \cdot 10^{-3}, 1.5 \cdot 10^{-3}]$	$4.3 \cdot 10^{-3}$	$[4.3 \cdot 10^{-3}, 4.4 \cdot 10^{-3}]$	$\mu m^2\ s^{-1}$
$\omega_{de\to D,E}$	$2.0 \cdot 10^{-1}$	$[2.0 \cdot 10^{-1}, 2.0 \cdot 10^{-1}]$	$2.9 \cdot 10^{-1}$	$[2.8 \cdot 10^{-1}, 3.0 \cdot 10^{-1}]$	s^{-1}
$\omega_{de\to D,e}$	$2.2 \cdot 10^{-4}$	$[0, 5.4 \cdot 10^{-4}]$	$2.7 \cdot 10^{-7}$	$[0, 1.7 \cdot 10^{-2}]$	s^{-1}
$\omega_{de\to d,e}$	$6.0 \cdot 10^{-2}$	$[6.0 \cdot 10^{-2}, 6.0 \cdot 10^{-2}]$	$5.9 \cdot 10^{-2}$	$[0, 7.3 \cdot 10^{-2}]$	s^{-1}
$\omega_{ded,e\to de,de}$	9.9	$[9.9, 9.9]$	$2.1 \cdot 10^{-14}$	$[0, 2.0 \cdot 10^{-3}]$	$\mu m^2\ s^{-1}$
$\omega_{ded\to d,de}$	$2.5 \cdot 10^{-2}$	$[2.4 \cdot 10^{-2}, 2.7 \cdot 10^{-2}]$	8.6	$[8.5, 8.6]$	s^{-1}
$\omega_{e\to E}$	$5.6 \cdot 10^{-2}$	$[5.6 \cdot 10^{-2}, 5.6 \cdot 10^{-2}]$	$5.5 \cdot 10^{-2}$	$[5.3 \cdot 10^{-2}, 5.6 \cdot 10^{-2}]$	s^{-1}

Table S6: Parameters from the fits of the AABSM to the oscillation data and the MinD dissociation data.
