# Supplementary material for: The mechanism of MinD stability modulation by MinE in Min protein dynamics
Source: PLoS Comput Biol. 2023 Nov 17;19(11):e1011615. doi: 10.1371/journal.pcbi.1011615 (PMC10691731; doi:10.1371/journal.pcbi.1011615)
Supplement: S7 Table — (PDF) [file pcbi.1011615.s024.pdf]

	Oscillation Data		MinD Dissociation Data		
Parameter	Value	95% Confidence Interval	Value	95% Confidence Interval	Units
C_d	$1.0 \cdot 10^2$	$[6.4, 1.9 \cdot 10^2]$	$1.7 \cdot 10^2$	$[0, 1.7 \cdot 10^2]$	μm^{-2}
C_d (w/o MinE)			0	$[0, 0]$	μm^{-2}
C_e	$9.6 \cdot 10^1$	$[6.2 \cdot 10^1, 1.3 \cdot 10^2]$	0	$[0, 2.8 \cdot 10^1]$	μm^{-2}
a	$1.4 \cdot 10^{-8}$	$[1.1 \cdot 10^{-8}, 1.9 \cdot 10^{-8}]$	$1.1 \cdot 10^{-11}$	$[1.1 \cdot 10^{-11}, 1.3 \cdot 10^{-11}]$	$\mu m^4\ s^{-1}$
b	$2.0 \cdot 10^{-1}$	$[1.7 \cdot 10^{-1}, 2.6 \cdot 10^{-1}]$	$1.4 \cdot 10^{-1}$	$[1.2 \cdot 10^{-1}, 1.5 \cdot 10^{-1}]$	s^{-1}
c	$1.2 \cdot 10^{-2}$	$[1.1 \cdot 10^{-2}, 1.2 \cdot 10^{-2}]$	$1.6 \cdot 10^{-2}$	$[1.5 \cdot 10^{-2}, 1.8 \cdot 10^{-2}]$	s^{-1}
d	$1.4 \cdot 10^{-2}$	$[1.3 \cdot 10^{-2}, 1.5 \cdot 10^{-2}]$	$1.0 \cdot 10^{-2}$	$[7.4 \cdot 10^{-3}, 1.4 \cdot 10^{-2}]$	s^{-1}
I	$6.0 \cdot 10^1$	$[4.6 \cdot 10^1, 7.8 \cdot 10^1]$	0	$[0, 3.3]$	$\mu m^{-2}\ s^{-1}$
v_l	$9.7 \cdot 10^2$	$[7.3 \cdot 10^2, 1.2 \cdot 10^3]$	$1.3 \cdot 10^4$	$[1.1 \cdot 10^4, 1.4 \cdot 10^4]$	μm^{-2}
v_u	$7.5 \cdot 10^3$	$[7.3 \cdot 10^3, 7.7 \cdot 10^3]$	$7.8 \cdot 10^5$	$[7.2 \cdot 10^5, 7.9 \cdot 10^5]$	μm^{-2}

Table S7: Parameters from the fits of the FHNM to the oscillation data and the MinD dissociation data.
