# Supplementary material for: The mechanism of MinD stability modulation by MinE in Min protein dynamics
Source: PLoS Comput Biol. 2023 Nov 17;19(11):e1011615. doi: 10.1371/journal.pcbi.1011615 (PMC10691731; doi:10.1371/journal.pcbi.1011615)
Supplement: S8 Table — γ = 16 in the constrained optimization. We presume that these are the most biologically relevant parameter estimates we have. See the caption of S10 Fig for details of the constrained fit, notably regarding changes to the AABSM involving rate parameters ωE,d→dez and ωE,ded→de,dez for z ∈ {∅, de, ded, e}. (PDF) [file pcbi.1011615.s025.pdf]

	Oscillation Data		MinD Dissociation Data		
Parameter	Value	95% Confidence Interval	Value	95% Confidence Interval	Units
C_d	$6.3 \cdot 10^1$	$[1.8 \cdot 10^1, 3.8 \cdot 10^1]$	$1.7 \cdot 10^2$	$[3.0 \cdot 10^1, 7.3 \cdot 10^1]$	μm^{-2}
C_d (w/o MinE)			$5.1 \cdot 10^1$	$[5.6, 2.8 \cdot 10^1]$	μm^{-2}
C_e	$2.0 \cdot 10^1$	$[4.8, 1.3 \cdot 10^1]$	0	$[0, 2.6]$	μm^{-2}
$c_{\bar{d}}$	$3.1 \cdot 10^1$	$[0, 1.7 \cdot 10^1]$	$8.3 \cdot 10^1$	$[1.4 \cdot 10^1, 3.6 \cdot 10^1]$	μm^{-2}
$c_{\bar{d}}$ (w/o MinE)			0	$[0, 3.4]$	μm^{-2}
$c_{\max}$	$5.3 \cdot 10^3$	$[5.2 \cdot 10^3, 5.3 \cdot 10^3]$			μm^{-2}
c_s	$4.4 \cdot 10^2$	$[3.9 \cdot 10^2, 4.6 \cdot 10^2]$	$2.1 \cdot 10^3$	$[2.0 \cdot 10^3, 2.3 \cdot 10^3]$	μm^{-2}
n_s	5.0	$[3.6, 6.4]$	1.9	$[1.8, 2.1]$	
$\omega_{D\to d}$	0	$[0, 1.7 \cdot 10^{-1}]$			$\mu m^{-2}\ s^{-1}$
$\omega_{D\to d}^{d}$	$3.5 \cdot 10^{-1}$	$[3.4 \cdot 10^{-1}, 3.6 \cdot 10^{-1}]$			s^{-1}
$\omega_{D\to d}^{de}$	$1.9 \cdot 10^{-1}$	$[1.9 \cdot 10^{-1}, 1.9 \cdot 10^{-1}]$			s^{-1}
$\omega_{D\to d}^{ded}$	$3.5 \cdot 10^{-1}$	$[3.4 \cdot 10^{-1}, 3.6 \cdot 10^{-1}]$			s^{-1}
$\omega_{E,d\to de}$	$2.1 \cdot 10^{-3}$	$[1.7 \cdot 10^{-3}, 2.2 \cdot 10^{-3}]$	$5.8 \cdot 10^{-4}$	$[4.4 \cdot 10^{-4}, 8.2 \cdot 10^{-4}]$	$\mu M^{-1}\ s^{-1}$
$\omega_{E,d\to de}^{de}$	$1.1 \cdot 10^{-7}$	$[0, 1.4 \cdot 10^{-6}]$	$1.8 \cdot 10^{-6}$	$[0, 2.1 \cdot 10^{-5}]$	$\mu M^{-1}\ \mu m^2\ s^{-1}$
$\omega_{E,d\to de}^{ded}$	$7.6 \cdot 10^{-7}$	$[6.8 \cdot 10^{-7}, 1.2 \cdot 10^{-6}]$	$1.2 \cdot 10^{-5}$	$[1.0 \cdot 10^{-5}, 1.3 \cdot 10^{-5}]$	$\mu M^{-1}\ \mu m^2\ s^{-1}$
$\omega_{E,d\to de}^{e}$	$1.8 \cdot 10^{-1}$	$[1.8 \cdot 10^{-1}, 1.8 \cdot 10^{-1}]$	$1.4 \cdot 10^{-2}$	$[1.3 \cdot 10^{-2}, 1.7 \cdot 10^{-2}]$	$\mu M^{-1}\ \mu m^2\ s^{-1}$
$\omega_{E,ded\to de,de}$	$2.7 \cdot 10^{-6}$	$[0, 2.5 \cdot 10^{-4}]$	$1.7 \cdot 10^{-7}$	$[0, 2.9 \cdot 10^{-3}]$	$\mu M^{-1}\ s^{-1}$
$\omega_{E,ded\to de,de}^{de}$	$9.5 \cdot 10^{-6}$	$[9.1 \cdot 10^{-6}, 9.9 \cdot 10^{-6}]$	$9.4 \cdot 10^{-6}$	$[1.1 \cdot 10^{-6}, 3.9 \cdot 10^{-5}]$	$\mu M^{-1}\ \mu m^2\ s^{-1}$
$\omega_{E,ded\to de,de}^{ded}$	$9.6 \cdot 10^{-6}$	$[9.0 \cdot 10^{-6}, 9.6 \cdot 10^{-6}]$	$3.4 \cdot 10^{-5}$	$[2.2 \cdot 10^{-5}, 4.6 \cdot 10^{-5}]$	$\mu M^{-1}\ \mu m^2\ s^{-1}$
$\omega_{E,ded\to de,de}^{e}$	$1.9 \cdot 10^{-2}$	$[1.9 \cdot 10^{-2}, 1.9 \cdot 10^{-2}]$	$2.9 \cdot 10^{-3}$	$[2.6 \cdot 10^{-3}, 3.0 \cdot 10^{-3}]$	$\mu M^{-1}\ \mu m^2\ s^{-1}$
$\omega_{d,de\to ded}$	$7.5 \cdot 10^{-5}$	$[7.4 \cdot 10^{-5}, 7.6 \cdot 10^{-5}]$	$9.7 \cdot 10^{-4}$	$[9.7 \cdot 10^{-4}, 1.2 \cdot 10^{-3}]$	$\mu m^2\ s^{-1}$
$\omega_{d,e\to de}$	$4.9 \cdot 10^{-2}$	$[4.8 \cdot 10^{-2}, 4.9 \cdot 10^{-2}]$	$3.1 \cdot 10^{-3}$	$[3.0 \cdot 10^{-3}, 3.1 \cdot 10^{-3}]$	$\mu m^2\ s^{-1}$
$\omega_{d\to D}$	$3.2 \cdot 10^{-1}$	$[3.1 \cdot 10^{-1}, 3.3 \cdot 10^{-1}]$	$1.9 \cdot 10^{-1}$	$[1.8 \cdot 10^{-1}, 2.0 \cdot 10^{-1}]$	s^{-1}
$\omega_{de,de\to ded,e}$	$5.3 \cdot 10^{-4}$	$[5.3 \cdot 10^{-4}, 5.3 \cdot 10^{-4}]$	$8.1 \cdot 10^{-3}$	$[8.1 \cdot 10^{-3}, 8.1 \cdot 10^{-3}]$	$\mu m^2\ s^{-1}$
$\omega_{de\to D,E}$	$1.7 \cdot 10^{-1}$	$[1.7 \cdot 10^{-1}, 1.7 \cdot 10^{-1}]$	$3.1 \cdot 10^{-1}$	$[2.9 \cdot 10^{-1}, 3.2 \cdot 10^{-1}]$	s^{-1}
$\omega_{de\to D,e}$	$1.7 \cdot 10^{-2}$	$[1.6 \cdot 10^{-2}, 1.7 \cdot 10^{-2}]$	$1.0 \cdot 10^{-3}$	$[1.0 \cdot 10^{-3}, 2.2 \cdot 10^{-2}]$	s^{-1}
$\omega_{de\to d,e}$	$2.0 \cdot 10^{-2}$	$[2.0 \cdot 10^{-2}, 2.0 \cdot 10^{-2}]$	$1.1 \cdot 10^{-1}$	$[8.5 \cdot 10^{-2}, 1.3 \cdot 10^{-1}]$	s^{-1}
$\omega_{ded,e\to de,de}$	1.0	$[1.0, 1.0]$	$6.6 \cdot 10^{-2}$	$[6.6 \cdot 10^{-2}, 6.6 \cdot 10^{-2}]$	$\mu m^2\ s^{-1}$
$\omega_{ded\to d,de}$	$3.7 \cdot 10^{-2}$	$[3.6 \cdot 10^{-2}, 3.8 \cdot 10^{-2}]$	$2.3 \cdot 10^{-3}$	$[2.3 \cdot 10^{-3}, 1.4 \cdot 10^{-2}]$	s^{-1}
$\omega_{e\to E}$	$5.7 \cdot 10^{-2}$	$[5.7 \cdot 10^{-2}, 5.8 \cdot 10^{-2}]$	$6.0 \cdot 10^{-2}$	$[5.9 \cdot 10^{-2}, 6.1 \cdot 10^{-2}]$	s^{-1}

Table S8: Parameters from the simultaneous fitting of the AABSM to the oscillation data and the MinD dissociation data with parameter constraints. $\gamma = 16$ in the constrained optimization. We presume that these are the most biologically relevant parameter estimates we have. See the caption of Fig. S10 for details of the constrained fit, notably regarding changes to the AABSM involving rate parameters $\omega_{E,d\to de}^{z}$ and $\omega_{E,ded\to de,de}^{z}$ for $z \in \{\emptyset, de, ded, e\}$.
